# Probing the prostate tumour microenvironment I: impact of glucose deprivation on a cell model of prostate cancer progression

**DOI:** 10.18632/oncotarget.14605

**Published:** 2017-01-12

**Authors:** Claire Tonry, John Armstrong, Stephen R. Pennington

**Affiliations:** ^1^ Conway Institute of Biomolecular and Biomedical Research, University College Dublin, Belfield, Dublin, Co. Dublin; ^2^ St. Lukes Hospital, Rathgar, Dublin, Co. Dublin

**Keywords:** prostate cancer, tumour microenvironment, biomarkers, proteomics, mass spectrometry

## Abstract

In the developed world, prostate cancer is the most common cancer diagnosis in men. Although prostate cancer initially presents as a non life-threatening disease, 90% of patients will develop castration resistant prostate cancer (CRPC), which preludes distant metastasis and is largely accountable for prostate cancer associated deaths. This is because as yet, there are no viable molecular therapeutic targets for effective treatment of CRPC. It is now widely accepted that cancer cells can alter their metabolic profile during the course of tumourgenesis and metastasis such that they are able to survive in oxygen and nutrient-poor environments. This work was aimed towards gaining greater mechanistic understanding of how such stresses in the tumour microenvironment impact on both androgen sensitive (LNCaP) and androgen independent (LNCaP-abl and LNCaP-abl-Hof) prostate cancer cell lines. Here we have applied technically robust and reproducible label-free liquid chromatography mass spectrometry analysis for comprehensive proteomic profiling of prostate cancer cell lines under nutrient deficient (low glucose) conditions. This led to the identification of approximately 4,000 proteins - one of the largest protein datasets for prostate cancer cell lines established to date. The biological and clinical significance of proteins showing a significant change in expression as result of low glucose conditions was established. Novel, intuitive workflows were subsequently implemented to ensure the verification of selected proteins of interest in a robust, reproducible and high throughput manner. Overall, these data suggest that this strategy supports identification of protein biomarkers of prostate cancer progression and potential therapeutic targets for CRPC.

## INTRODUCTION

Prostate cancer (PCa) is the second most common cancer-related cause of death in men worldwide [1–3]. In many cases, patients will present with an indolent, non-aggressive form of disease, which can be effectively managed by radical prostatectomy, radiotherapy, hormone therapy and/or active surveillance. Unfortunately, over time many men cease to respond to these treatments and progress to a more aggressive form of PCa, referred to as castration resistant prostate cancer (CRPC) [4]. Currently, there are no effective molecularly targeted treatment strategies for metastatic CRPC [5]. Identification of suitable molecular targets for management of CRPC requires greater mechanistic insight into PCa progression and the PCa tumour microenvironment.

As with all solid tumours, the host microenvironment is greatly transformed during prostate tumor growth and tumours will be exposed to some degree of hypoxia and nutrient deprivation as they out-grow their blood supply and their metabolic activity is increased [6–8]. It is therefore widely accepted that the tumour microenvironment plays an influential role in survival, angiogenesis, inflammation and metastatic dissemination of cancer cells. Previous studies have shown that cancer cell lines are able to survive longer when exposed to extremely low nutrient supply [6]. Another study has demonstrated that glucose deprivation actually increases resistance of cancer cells to radiation treatment [8]. To date, however, the role of nutrient (glucose deprivation) in cancer cell metabolism and PCa progression has not been fully elucidated [9].

Proteome scale technology has improved significantly in recent years and mass spectrometry (MS) has emerged as a key enabling technology for biomarker discovery and the identification of novel therapeutic targets [10]. Proteomic analysis by MS provides a comprehensive picture of changes in protein expression and the vast amount of information obtained can facilitate greater understanding of cancer cells’ adaption and survival in the tumour microenvironment [11, 12]. However, the success of any MS-based proteomics investigation is highly dependent upon the rigour in which the entire workflow - sample preparation, MS analysis, data analysis and biological interpretation of the data - is undertaken.

In this study we have used the androgen sensitive LNCaP cell line and its androgen independent progeny, LNCaP-abl (Abl) and LNCaP-abl-Hof (Hof), to investigate the effects of glucose deprivation in a cell line model of progression to CRPC. Specific aims of this study were (i) to attempt to identify potential therapeutic targets for treatment of CRPC and (ii) to identify potential protein biomarkers which may be indicative of important changes within the tumour microenvironment that drive progression of CRPC. Each stage of the investigative process was carefully executed, with the implementation of novel workflows, to ensure that (i) MS analysis was performed on samples of high quality, (ii) observed changes in protein expression were not influenced by any experimental or technical bias (iii) potential biological and/or clinical significance was established for any identified proteins of interest and (iv) further verification of selected proteins of interest could be performed in a robust, reproducible and high throughput manner.

## RESULTS

### Proteomic analysis of the effect of low glucose conditions

The androgen sensitive LNCaP cell line, as well as its androgen independent progeny, LNCaP-abl (Abl) and LNCaP-abl-Hof (Hof), were used for this study as they represent a good model for progression to CRPC in a consistent genetic background. To induce “low glucose” conditions, PCa cell lines were cultured in RPMI (-Glucose) media supplemented with 5mM Glucose. This glucose concentration was chosen so as to not evoke a general stress response within the cells, which may occur by using concentrations of glucose lower than 5mM [9, 13–15]. Hence, the carefully selected five fold reduction in the concentration of glucose used in these *in vitro* experiments can be considered reflective of changes encountered by the prostate tumour cells *in vivo* (13). Similarly, FCS was not omitted from the culture media when generating a low glucose microenvironment so as to avoid changes induced by restricting the proliferative capacity of the cells and potentially inducing apoptosis in a manner that varied between the cell lines [16]. Therefore, by undertaking the experiments in the presence of FCS, the observed proteome-wide alterations in protein expression are more likely to be solely a consequence of the change in glucose concentration.

**Figure 1:**
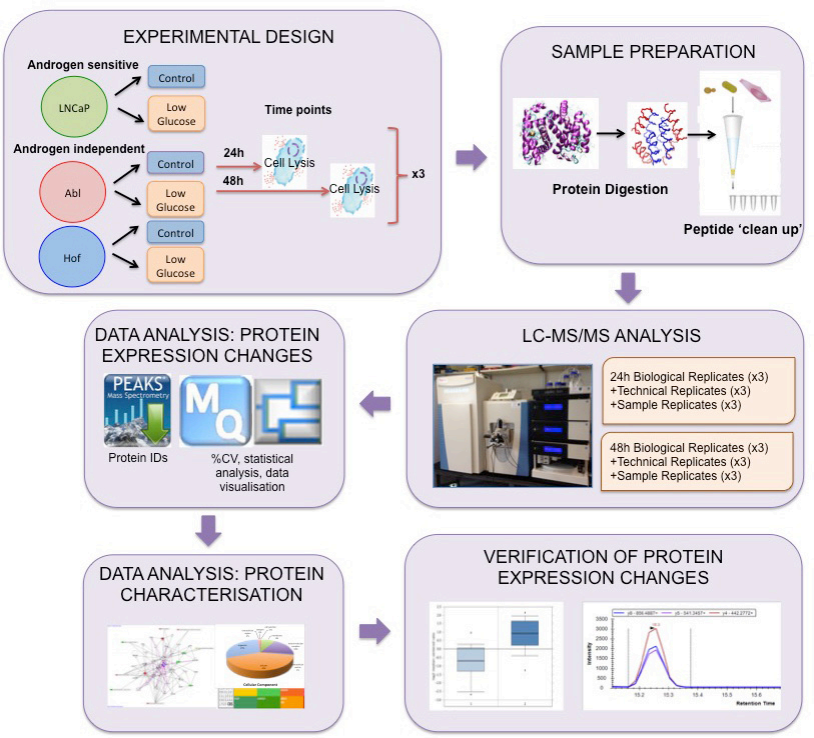
Experimental Workflow for Proteome scale analysis of the impact of glucose deprivation in prostate cancer cells Androgen sensitive (LNCaP) and androgen independent (Abl and Hof) cell lines were incubated under standard (control) cell growth media and low glucose (LG) media for 24 h and 48 h. Lysed cells were digested with trypsin and peptides were purified using C-18 stage tips. Samples prepared from both time points were analysed *via* LC-MS/MS on a Q-Exactive mass spectrometer with technical replicates (TR) and sample replicates (SR) analysed throughout each run. Data analysis was performed using PEAKS, MaxQuant and Perseus software. Subsequent in silico biological interrogation and validation of protein expression changes was performed using PANTHER, IPA and SurvExpress software. MRM assays were designed to further evaluate prioritized proteins of interest.

**Figure 2:**
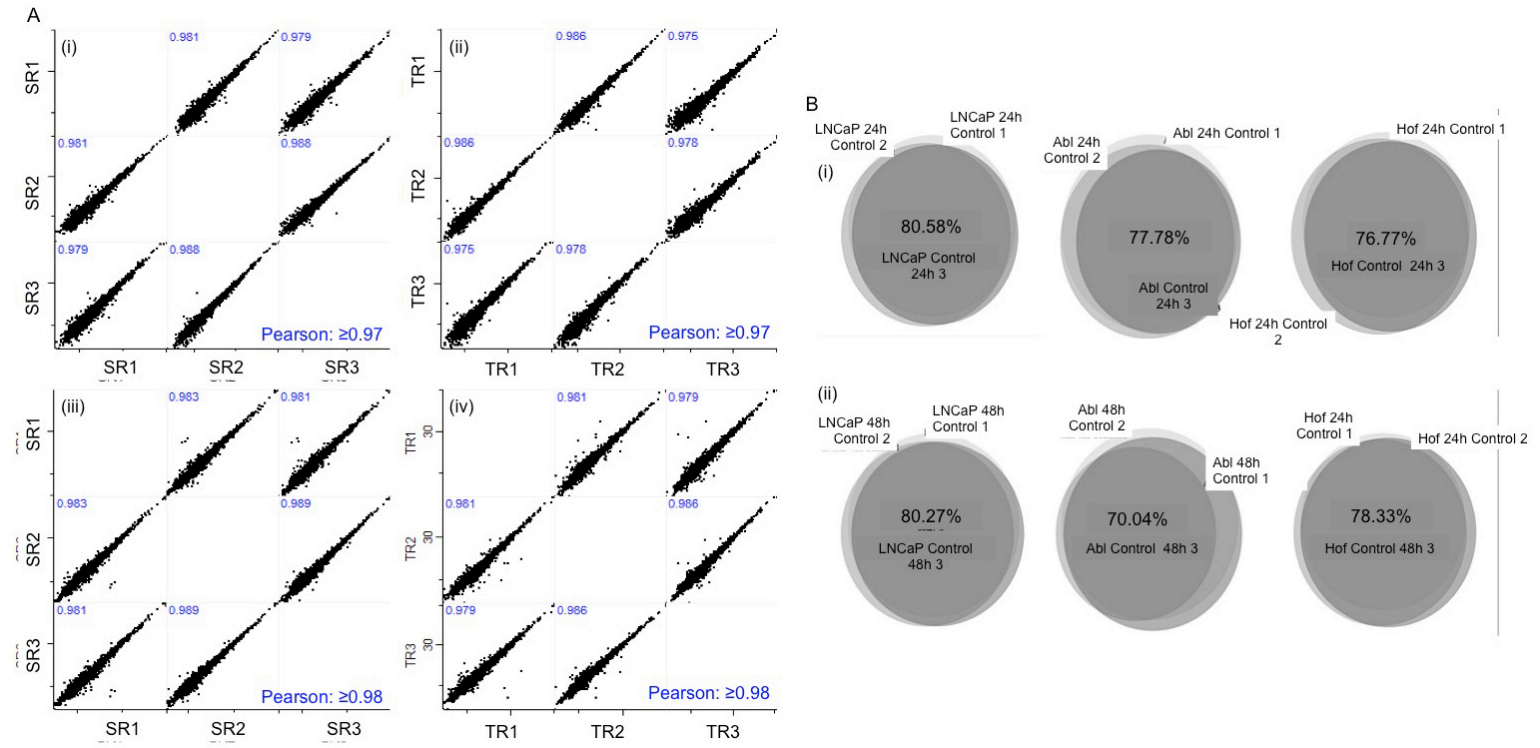
Validation of experimental design and analytical robustness Biologcal replicates (x3) were generated for all cell lines incubated in low glucose and standard media for 24 and 48 hours. Sample and Technical replicates were analysed at the beginning, middle and end of LC-MS analysis of samples from both time points. Experimental reproducibility was confirmed with scatter plots showing Pearson Correlation values ≥ 0.97 at both 24 hours (A(i)-(ii)) and 48 hours (A(iii)-(iv)). Biological reproducibility was established with at least 70% overlap in the proteins identified in replicate samples for each control cell line at 24 (B(i)) hour and 48 hour time points (B(ii)).

It has previously been observed that no significant changes in cellular metabolism are observed before 48 hours [17], however, we hypothesise that global proteomic changes occurring at this early stage in tumor growth may be of biological significance. As such, proteomic analysis of the PCa cell lines was undertaken after 24 and 48-hour incubation under low glucose conditions. Three independent biological replicates were prepared for LC-MS/MS analysis as outlined in Figure 1. The resulting raw LC-MS/MS data was analysed though XCalibar and Peaks (version 7) software to assess the quality of chromatograms and number of protein identifications acquired for each sample. Over 3,000 proteins were identified across all samples at both time points and were found to belong to the following subcellular regions; cell part (42%), organelle (30%), macromolecular complex (18%) and membrane (8%). The raw data was then processed through the Andromeda search engine of MaxQuant (version 1.4.1.2) software in preparation for further statistical characterization using Perseus (version 1.5.0.15) software. Analysis of sample replicate (SR), technical replicate (TR) and biological replicate data demonstrated excellent reproducibility throughout the entire experimental workflow, thereby giving high confidence to the subsequent observations made on protein expression changes as result of low glucose conditions (Figure 2).

### Statistical characterization of proteomic changes induced by low glucose conditions

**Figure 3:**
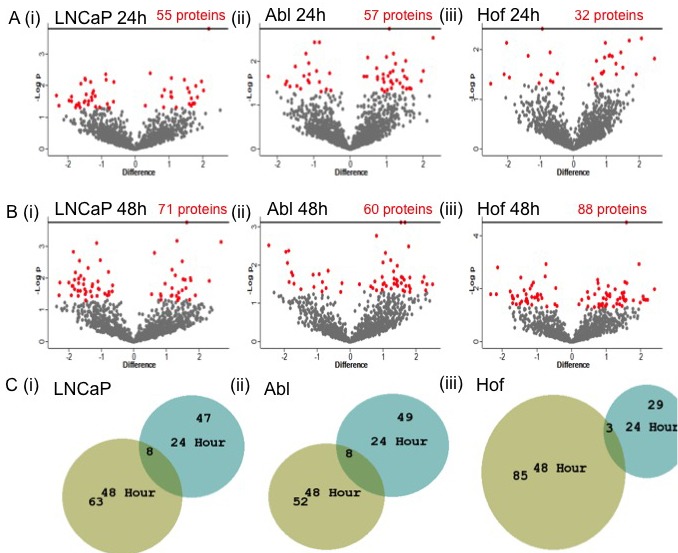
Significantly changing proteins in response to low glucose conditions in PCa cell lines Student's *t*-test analysis (*p* < 0.05) was performed on each cell line to determine the effects of incubation in low glucose on protein expression. The volcano plots reflect the statistically significantly changed proteins (red) identified from Student's *t*-test analysis of each cell line after 24h **A**. and 48h **B**. culturing in low glucose conditions. A small number of proteins were found to be commonly significantly changed within each cell line at both time points **C**.

To further characterize low-glucose related proteomic changes, student's *t*-test analysis was carried out to identify proteins showing a significant change in expression (*p* ≤ 0.05) between cells incubated in low glucose media and their respective controls, at both time points. At 24 hours 55, 57 and 32 proteins were significantly up or down regulated as result of low glucose conditions in the LNCaP, Abl and Hof cell lines, respectfully (Figure 3A). At 24 hours the protein ‘serotransferrin’ was significantly up regulated in each of the cell lines that were incubated under low glucose conditions. The protein ‘DnaJ homolog subfamily C member 7’ was significantly up regulated in only the androgen independent Abl and Hof cell lines after 24-hour incubation in low glucose. At 48 hours there was, as expected, an increase in the numbers of proteins showing a significant change in expression after cell growth under low glucose conditions. At this time point 71, 60 and 80 proteins were significantly up or down regulated in the LNCaP, Abl and Hof cell lines, respectfully (Figure 3B). Of these a significant change in expression was commonly observed for 2 proteins across all cell lines following incubation with low glucose, that being an up-regulation in ‘serotransferrin’ and down regulation in ‘proactivator polypeptide’. There were 9 proteins commonly significantly up regulated in the androgen independent cell lines after 48 hour incubation in low glucose conditions - ‘l-lactate dehydrogenase B chain’; ‘proliferating cell nuclear antigen’; ‘UDP-N-acetylhexosamine’; ‘heat shock protein 105 kDa’; ‘cAMP-dependent protein kinase type I-alpha regulatory subunit’; ‘Tryptophan—tRNA ligase, cytoplasmic’; ‘Threonine—tRNA ligase, cytoplasmic’; ‘Ran-specific GTPase-activating protein’ and ‘Rho GDP-dissociation inhibitor 1’. Two proteins were commonly significantly down regulated in the androgen independent cell lines following 48-hour incubation in low glucose conditions - ‘Polypyrimidine tract-binding protein 1’ and ‘Very long-chain specific acyl-CoA dehydrogenase, mitochondrial’. It is worth noting that serotransferrin - the only protein that was commonly up regulated in all cell lines in response to low glucose conditions at both time points - has previously been identified in biomarker studies related to pancreatic cancer, stomach cancer and colorectal cancer [18–20]. However, it is doubted that serotransferrin would ever be of great use as a biomarker due to it's association with other inflammatory diseases including coronary disease and bacterial infection [18].

Additional notable changes in protein expression as result of low glucose conditions were observed for proteins associated with the Warburg effect, namely ‘L-lactate dehydrogenase A chain’, ‘L-lactate dehydrogenase B chain’ and ‘fatty acid synthase’. The protein ‘cathepsin D’ was also up regulated as result of low glucose conditions in the three PCa cell lines. This protein is known to be associated with autophagy. The connection between autophagy and cancer cell metabolism is considered to be of great clinical relevance in cancer research as metabolically stressed tumour cells rely on autophagy for reprogramming of their metabolism to accommodate their rapid growth and proliferation [21]. Indeed, for all of the cell lines the significantly changed proteins identified at both time points map onto metabolic processes and other biological processes associated with deregulated metabolism. Significantly up or down-regulated proteins that were commonly identified within each cell line at both the 24 and 48 hour time point are listed in Table 1. The full list of significantly up or down regulated proteins identified within each cell line at both time points are listed in Supplementary Data Table S1. Overall, this analysis has led to the identification of a number of proteins that are differentially regulated in response to low glucose conditions and appear to be indicative of altered metabolism in prostate cancer cells, even after just 24 hours. These proteins may therefore be of potential clinical significance to PCa progression.

**Table 1:** Common Significant Protein Changes as Result of Low Glucose Conditions

Protein names	Protein IDs	Gene names	24 Hours	48 hours
**LNCaP**				
Secretory carrier-associated membrane protein 3	O14828	SCAMP3	-1.68	-1.73
Transferrin receptor protein 1	P02786	TFRC	-1.45	-1.91
**Serotransferrin**	**P02787**	**TF**	**+2.18**	**+2.68**
Myosin-10	P35580	MYH10	-0.64	+0.54
Hepatoma-derived growth factor	P51858	HDGF	+1.07	+1.74
Probable ATP-dependent RNA helicase DDX17	Q92841	DDX17	+1.08	-1.33
RNA-binding protein 14	Q96PK6	RBM14	+1.67	-1.15
Extended synaptotagmin-1	Q9BSJ8	ESYT1	-1.77	-1.87
**Abl**				
**Serotransferrin**	**P02787**	**TF**	**+2.01**	**+1.33**
Glucose-6-phosphate isomerase	P06744	GPI	+1.45	+0.97
T-complex protein 1 subunit alpha	P17987	TCP1	+1.18	+0.80
Polypyrimidine tract-binding protein 1	P26599	PTBP1	-0.98	-0.94
Threonine--tRNA ligase, cytoplasmic	P26639	TARS	+0.90	+1.32
Stress-induced-phosphoprotein 1	P31948	STIP1	+1.12	+1.33
ATPase inhibitor, mitochondrial	Q9UII2	ATPIF1	+1.02	+1.78
**Hof**				
Eukaryotic translation initiation factor 3 subunit H	O15372	EIF3H	+0.70	+1.99
**Serotransferrin**	**P02787**	**TF**	**+2.08**	**+1.55**
cAMP-dependent protein kinase type I-alpha regulatory subunit	P10644	PRKAR1A	-2.04	+1.51

### Network and pathway analysis of the molecular changes occurring as result of low glucose conditions

In-depth analysis of the molecular changes occurring as result of low glucose conditions for both the androgen sensitive (LNCaP) and androgen independent (Abl and Hof) cell lines was conducted using Ingenuity Pathway Analysis (IPA) software. A search of the proteins that were either significantly up or down regulated as result of low glucose conditions revealed that low glucose treatment appeared to activate molecules that map to pathways associated with cell death and apoptosis - with a more extreme effect observed after 48 hours. The IPA software also indicated which of the deregulated proteins have previously been associated as a disease biomarker and/or can be targeted by known therapeutic agents.

### The loss of androgen sensitivity measured by proteomic analysis

**Figure 4:**
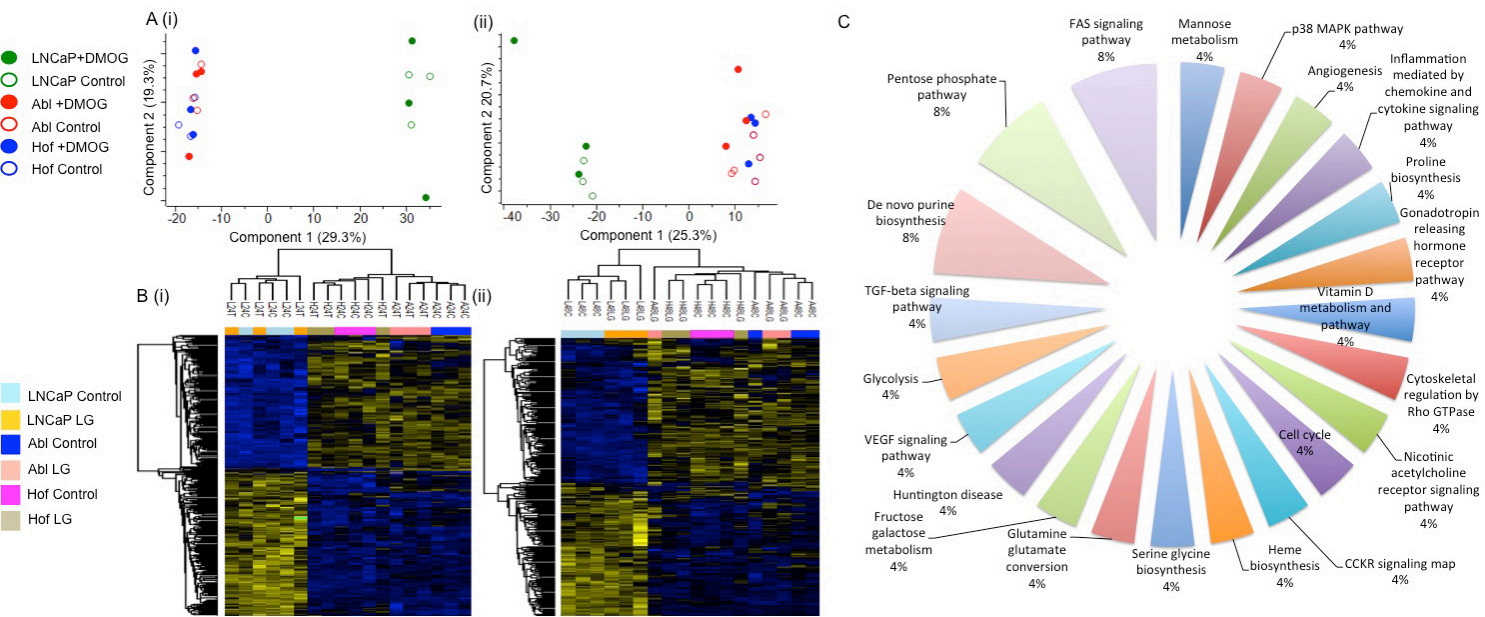
Proteomic Characterisation of Androgen Sensitive and Androgen Independent PCa cell lines The androgen sensitive (LNCaP) and androgen independent (Abl and Hof) show clear differences in protien epression, irrespective of low glucose conditions. Principal Component Analysis revealed clear separation between Androgen Sensitive and Androgen Independent PCa cell lines at both 24 hour (A(i)) and 48 hour (A(ii)) time points. ANOVA (*p* ≤ 0.05, Benjamani-Hochberg FDR) analysis revealed 324 and 259 significantly changing proteins at 24 hours (B(i)) and 48 hours (B(ii)) respectively. Pathway analysis was conducted on the 133 proteins which show a significant change in expression at both time points. The most highly represented pathways are the FAS signaling, Pentose Phosphate and De novo purine biosynthesis pathways. The remaining equally represented pathways, represented by significantly changing protiens between androgen sensitive and androgen independent cell lines, are highly associated with cancer progression (C).

The cell lines used for this study - LNCaP, Abl and Hof - represent a model of PCa progression from a less aggressive androgen sensitive phenotype (LNCaP) to a more aggressive androgen independent (CRPC) phenotype (Abl and Hof) [22–24]. Therefore, unbiased analysis of these cell lines following incubation in both low glucose and nutrient rich conditions by LC-MS/MS also allowed us to make observations on the molecular impact of androgen sensitivity. Principal component analysis of the data demonstrated a clear separation between the androgen sensitive (LNCaP) and androgen independent (Abl and Hof) cell lines at both time points (Figure 4A). An analysis of variance (ANOVA) test was carried out to identify significantly changing proteins across all samples at both time points with Benjamani Hochberg (*p* ≤ 0.05) false discovery rate applied for truncation. At the 24-hour time point, 324 significantly changing proteins were identified across all samples and at 48 hours 259 significantly changing proteins were identified across all samples. Again, these proteomic changes indicated a clear separation between the androgen sensitive (LNCaP) and androgen independent (Abl and Hof) PCa cell lines (Figure 4B). Across both the 24-hour and 48-hour time points 133 proteins were commonly identified as significantly changed with Benjamani-Hochberg FDR (*p* ≤ 0.05) applied. These proteins map to a number of ‘cancer-associated’ pathways such as the FAS signaling pathway, TGF-beta signaling pathway, angiogenesis and VEGF signaling pathways (Figure 4C). Notably, a number of proteins that play a role in the adaptive metabolic response of cancer cells to the tumour microenvironment were among those with a significant change in expression between androgen independent and androgen sensitive cell lines. The protein ‘L-lactate dehydrogenase A chain’ was found to be up regulated in the androgen independent (Abl and Hof) cell lines while contrastingly, the protein ‘L-lactate dehydrogenase B chain’ was up regulated in the androgen sensitive (LNCaP) cell line. The protein ‘nicotinamide phosphoribosyltransferase’, which plays a fundamental role in providing an energy source for cancer cells through the biosynthesis of NAD+, was also among the identified proteins found to be significantly up regulated in the androgen independent cell lines. The lysosmal enzyme ‘cathepsin D’, as well as a number of proteins involved in glutamine metabolism (‘bifunctional glutamate’, ‘glutamate dehydrogenase 1’ and ‘isocitrate dehydrogenase [NADP] cytoplasmic’) were also up regulated in the androgen independent cell lines. The complete list of proteins commonly identified at both time points with a significant change in expression, along with their associated KEGG pathways are listed in Supplementary Data .2. Of the complete list of proteins which show a statistically significant change in expression between androgen independent and androgen sensitive PCa cell lines at both time points, the majority have also been identified as being significantly deregulated between androgen sensitive and androgen independent PCa cell lines in a separate study investigating the effects of hypoxia on PCa progression, using the same cell lines (manuscript under review). As such, there is evidence to suggest that the observations made here on protein expression changes can reliably be attributed to loss of androgen sensitivity in PCa cell lines and are likely to be of biological relevance to the development of CRPC.

Based on the proteins identified through ANOVA analysis of the LC-MS/MS data, the top up and down regulated diseases and biological functions associated with loss of androgen sensitivity were identified for both time points through IPA analysis. Those that have previous association as a biomarker and/or therapeutic target were also identified in this analysis (Table 2).

**Table 2:** Drug Targets Identified Through IPA Analysis of Significantly Changing Proteins Between Androgen Sensitive and Androgen Independent Cell Lines

Symbol	Entrez Gene Name	Location	Type(s)	Biomarker Application(s)	Drug(s)
**24 Hours (AS v AI)**					
**ABCC4**	ATP-binding cassette, sub-family C (CFTR/MRP), member 4	Plasma Membrane	transporter	prognosis	
**AIM1**	absent in melanoma 1	Extracellular Space	other	diagnosis,disease progression,prognosis	
**ANXA5**	annexin A5	Plasma Membrane	transporter	diagnosis,efficacy	
**CDH1**	cadherin 1, type 1	Plasma Membrane	other	diagnosis,disease progression,efficacy,prognosis	
**CSTB**	cystatin B (stefin B)	Cytoplasm	peptidase	diagnosis	
**CTSD**	cathepsin D	Cytoplasm	peptidase	diagnosis,unspecified application	
**FN1**	fibronectin 1	Extracellular Space	enzyme	diagnosis,efficacy,prognosis,unspecified application	ocriplasmin, L19-IL2 monoclonal antibody-cytokine fusion protein
**HSPB1**	heat shock 27kDa protein 1	Cytoplasm	other	diagnosis,efficacy	
**KRT18**	keratin 18, type I	Cytoplasm	other	efficacy	
**MME**	membrane metallo-endopeptidase	Plasma Membrane	peptidase	diagnosis,efficacy,unspecified application	
**NAMPT**	nicotinamide phosphoribosyltransferase	Extracellular Space	cytokine	diagnosis,prognosis	
**NUCB1**	nucleobindin 1	Cytoplasm	other	diagnosis	
**PARP1**	poly (ADP-ribose) polymerase 1	Nucleus	enzyme	diagnosis,efficacy,prognosis	poly ADP ribose polymerase 1 inhibitor, veliparib, rucaparib, olaparib, E7449, ABT-767, CEP-9722, INO-1001
**PDIA3**	protein disulfide isomerase family A, member 3	Cytoplasm	peptidase	diagnosis	
**PHB**	prohibitin	Nucleus	transcription regulator	diagnosis	
**STAT3**	signal transducer and activator of transcription 3 (acute-phase response factor)	Nucleus	transcription regulator	diagnosis,efficacy,prognosis,response to therapy	
**TF**	transferrin	Extracellular Space	transporter	efficacy,prognosis,unspecified application	ferric carboxymaltose
**TFRC**	transferrin receptor	Plasma Membrane	transporter	diagnosis,efficacy	
**48 Hours (AS v AI)**					
**ANXA5**	annexin A5	Plasma Membrane	transporter	diagnosis,efficacy	
**CTSD**	cathepsin D	Cytoplasm	peptidase	diagnosis,unspecified application	
**EEF1A2**	eukaryotic translation elongation factor 1 alpha 2	Cytoplasm	translation regulator	prognosis,unspecified application	
**FN1**	fibronectin 1	Extracellular Space	enzyme	diagnosis,efficacy,prognosis,unspecified application	ocriplasmin, L19-IL2 monoclonal antibody-cytokine fusion protein
**HSPB1**	heat shock 27kDa protein 1	Cytoplasm	other	diagnosis,efficacy	
**HSPD1**	heat shock 60kDa protein 1 (chaperonin)	Cytoplasm	enzyme	diagnosis,prognosis	
**MME**	membrane metallo-endopeptidase	Plasma Membrane	peptidase	diagnosis,efficacy,unspecified application	
**NAMPT**	nicotinamide phosphoribosyltransferase	Extracellular Space	cytokine	diagnosis,prognosis	
**PARP1**	poly (ADP-ribose) polymerase 1	Nucleus	enzyme	diagnosis,efficacy,prognosis	poly ADP ribose polymerase 1 inhibitor, veliparib, rucaparib, olaparib, E7449, ABT-767, CEP-9722, INO-1001
**PCNA**	proliferating cell nuclear antigen	Nucleus	enzyme	efficacy,prognosis,response to therapy	
**PDIA3**	protein disulfide isomerase family A, member 3	Cytoplasm	peptidase	diagnosis	
**PHB**	prohibitin	Nucleus	transcription regulator	diagnosis	
**PRDX1**	peroxiredoxin 1	Cytoplasm	enzyme	diagnosis	
**STOML2**	stomatin (EPB72)-like 2	Plasma Membrane	other	prognosis	
**TF**	transferrin	Extracellular Space	transporter	efficacy,prognosis,unspecified application	ferric carboxymaltose
**TFRC**	transferrin receptor	Plasma Membrane	transporter	diagnosis,efficacy	

AS = Androgen Sensitive; AI = Androgen Independent

### Prioritisation of proteins for further evaluation

. Instead of arbitrarily selecting a handful of these proteins to further evaluate by traditional antibody-based techniques, we sought to establish an approach for evaluation of as many proteins as possible by multiple reaction monitoring (MRM), a more high-throughput and cost-effective approach that does not rely on the availability of antibodies. To prioritise those that should be brought forward for further verification, we selected proteins that had been measured reproducibly in the LC-MS/MS analysis with coefficient of variance (CV) values ≤ 20%. Although recent reports suggest that MRM technology is certainly capable of analyzing over 100 proteins in one experiment, this is dependent on the use of internal labeling strategies [25]. So, we sought to minimize this selection further by prioritizing those proteins that, as indicated by IPA analysis, have previous association as a biomarker and/or drug target. In total this left a final list of 31 proteins representing a ‘signature’ of low glucose (LG)-mediated molecular changes. This panel of proteins was therefore annotated as the LG panel (Table 3).

**Table 3:** Proteins Selected for Low Glucose (LG) Panel

Low Glucose Panel											
Accession	Protein Name	Gene Name	LNCaP 24h	Abl 24h	Hof 24h	LNCaP 48h	Abl 48h	Hof 48h	BM/DT	Secreted	Exocarta/ Vesiclepedia
**P38606**	ATPase, H+ transporting, lysosomal 70kDa, V1 subunit A	ATP6V1A						-1.715	DT		✓
**P04040**	catalase	CAT				-1.804			DT		✓
**P12830**	cadherin 1, type 1	CDH1	-1.671						DT	✓	✓
**P33316-2**	deoxyuridine triphosphatase	DUT		+1.190					DT		✓
**P23588**	eukaryotic translation initiation factor 4B	EIF4B						+1.305	DT		✓
**P49327**	fatty acid synthase	FASN						+1.007	DT		✓
**P02794**	ferritin, heavy polypeptide 1	FTH1		+0.909					DT		✓
**P40939**	hydroxyacyl-CoA dehydrogenase	HADHA				-1.277			DT		✓
**P08238**	heat shock protein 90kDa alpha (cytosolic), class B member 1	HSP90AB1		+1.040					DT		✓
**P11021**	heat shock 70kDa protein 5 (glucose-regulated protein, 78kDa)	HSPA5						+1.495	DT	✓	✓
**P10809**	heat shock 60kDa protein 1 (chaperonin)	HSPD1				-1.492			DT		✓
**P14735**	insulin-degrading enzyme	IDE				+1.180			DT	✓	✓
**P52292**	karyopherin alpha 2 (RAG cohort 1, importin alpha 1)	KPNA2			+1.516				BM, DT		✓
**P28838-2**	leucine aminopeptidase 3	LAP3	-1.889						BM, DT		✓
**P33991**	minichromosome maintenance complex component 4	MCM4			+1.095				BM, DT		✓
**P55786**	aminopeptidase puromycin sensitive	NPEPPS			-1.048				BM, DT		✓
**P06748-3**	nucleophosmin (nucleolar phosphoprotein B23, numatrin)	NPM1				-1.547			BM, DT		✓
**P12004**	proliferating cell nuclear antigen	PCNA					+1.445	+1.454	BM, DT		✓
**P30086**	phosphatidylethanolamine binding protein 1	PEBP1				+1.348			BM, DT		✓
**P35232**	prohibitin	PHB		-1.127					BM	✓	✓
**P00491**	purine nucleoside phosphorylase	PNP				+1.625		+1.213	BM		✓
**Q99460-2**	proteasome 26S subunit, non-ATPase 1	PSMD1	-1.485						BM		✓
**P43487**	RAN binding protein 1	RANBP1					+1.434	+2.145	BM		✓
**P42677**	ribosomal protein S27	RPS27			+1.705				BM		✓
**P82979**	SAP domain containing ribonucleoprotein	SARNP			+1.447				BM		✓
**P05141**	solute carrier family 25 (mitochondrial carrier; adenine nucleotide translocator), member 5	SLC25A5						-2.331	BM		✓
**P31948**	stress-induced phosphoprotein 1	STIP1		+1.119			+1.329		BM		✓
**P02787**	transferrin	TF	+2.184	+2.006	+2.076	+2.682	+1.330	+1.545	BM	✓	✓
**P02786**	transferrin receptor	TFRC	-1.451	-1.466		-1.906		-1.312	BM		✓
**P06753-2**	tropomyosin 3	TPM3						+1.789	BM		✓
**P30536**	translocator protein (18kDa)	TSPO						-1.557	BM		✓

TR = Technical replicate; AS = Androgen sensitive; AI – Androgen independent

Oncomine gene summary: Green = down regulated, red = up regulated, 1-6 = number of studies reporting association

Due to the reported associations between loss of androgen sensitivity and development of CRPC, we also sought to verify protein changes observed between the androgen sensitive (LNCaP) and androgen independent (Abl and Hof) cell lines irrespective of low glucose or control conditions. Based on %CV values and previous association as a biomarker and/or drug target, this resulted in a final list of 35 proteins for evaluation as a ‘signature’ of androgen sensitivity (AS) in PCa cell lines (Table 4). Both panels include proteins (named previously), which are known to play a role in the altered metabolic activity of cancer cells.

**Table 4:** Proteins selected for Androgen Sensitivity (AS) Panel

AS Panel							
Protein IDs	Protein names	Gene names	%CV TR	Biomarker/Drug Target	AS v AI	Secreted	Exocarta/Vesiclpedia
**P04792**	Heat shock protein beta-1	HSPB1	0.4	BM	Up		✓
**P07099**	Epoxide hydrolase 1	EPHX1	4.5	BM	Up	✓	✓
**P07339**	Cathepsin D;Cathepsin D light chain;Cathepsin D heavy chain	CTSD	5.0	BM	Up	✓	✓
**O75369-2**	Filamin-B	FLNB	5.4	BM	Up		✓
**P02794**	Ferritin heavy chain	FTH1	7.7	BM	Up		✓
**P04080**	Cystatin-B	CSTB	8.0	BM	Up		✓
**Q7KZF4**	Staphylococcal nuclease domain-containing protein 1	SND1	9.8	BM	Up		✓
**P13667**	Protein disulfide-isomerase A4	PDIA3	5.20	BM	Up	✓	✓
**O75369-2**	Filamin-B	PRDX1	6.35	BM	Up		✓
**O75874**	Isocitrate dehydrogenase [NADP] cytoplasmic	CTSD	16.44	BM	Up	✓	✓
**P07195**	L-lactate dehydrogenase B chain	LDHB	5.0	BM	Down		✓
**P13010**	X-ray repair cross-complementing protein 5	XRCC5	5.4	BM	Down		✓
**P21333-2**	Filamin-A	FLNA	7.5	BM	Down		✓
**P35232**	Prohibitin	PHB	10.5	BM	Down	✓	✓
**P02786**	Transferrin receptor protein 1;Transferrin receptor protein 1, serum form	TFRC	11.0	BM	Down		✓
**Q8NCW5-2**	NAD(P)H-hydrate epimerase	APOA1BP	11.8	BM	Down	✓	✓
**O15439-2**	Multidrug resistance-associated protein 4	ABCC4	13.3	BM	Down		✓
**Q01105**	Protein SET	SET	14.6	BM	Down		✓
**Q9Y4K1**	Absent in melanoma 1 protein	AIM1	26.2	BM	Down		✓
**Q02818**	Nucleobindin-1	NUCB1	38.7	BM	Down	✓	✓
**P04843**	Dolichyl-diphosphooligosaccharide--protein glycosyltransferase subunit 1	XRCC6	4.89	BM	Down	✓	✓
**P49755**	Transmembrane emp24 domain-containing protein 10	HSPD1	5.78	BM	Down	✓	✓
**Q16891-2**	Mitochondrial inner membrane protein	EEF1A2	7.20	BM	Down	✓	✓
**Q13011**	Delta(3,5)-Delta(2,4)-dienoyl-CoA isomerase, mitochondrial	STOML2	7.34	BM	Down		✓
**Q5JPE7-2**	Nodal modulator 3;Nodal modulator 2	XRCC5	7.84	BM	Down	✓	✓
**Q99832-3**	T-complex protein 1 subunit eta	SET	9.73	BM	Down		✓
**P27797**	Calreticulin	PHB	18.21	BM	Down		✓
**P33316-2**	Deoxyuridine 5-triphosphate nucleotidohydrolase, mitochondrial	DUT	1.5	BM, DT	Down		✓
**P23786**	Carnitine O-palmitoyltransferase 2, mitochondrial	CPT2	6.1	BM, DT	Down		✓
**P12268**	Inosine-5-monophosphate dehydrogenase 2	IMPDH2	11.1	BM, DT	Down		✓
**P02751-17**	Fibronectin;Anastellin;Ugl-Y1;Ugl-Y2;Ugl-Y3	FN1	15.2	BM, DT	Down	✓	✓
**Q14938-5**	Nuclear factor 1 X-type	PARP1	3.71	BM, DT	Down		✓
**P80303-2**	Nucleobindin-2;Nesfatin-1	FN1	34.12	BM, DT	Down	✓	✓
**P17987**	T-complex protein 1 subunit alpha	ATP1A1	2.53	DT	Down		✓
**Q92598-2**	Heat shock protein 105 kDa	IMPDH2	6.74	DT	Down		✓
**O15355**	Protein phosphatase 1G	GMPS	7.82	DT	Down		✓

TR = Technical replicate; BM/DT = Biomarker/Drug Target

### Biological interrogation of selected proteins of interest

The data acquired in this study have potential to be highly informative about the molecular mechanisms of metabolic reprogramming, particularly the signaling events which may facilitate aberrant metabolic fluxes. As such, the prioritized panels of proteins were further interrogated to assess their biological relevance to the development of aggressive PCa. Proteins were assessed for their signaling activity, their sub cellular location and whether they are catagorised as ‘secreted’ proteins. To assess signaling activity proteins were searched through the ExoCarta and Vesiclepedia databases, both of which provide lists of proteins that have previously been identified in exosome-based studies. Cellular communication through exosomes or other extracellular vesicles is thought to play a key role in cancer [26]. It was found that all proteins in both the LG and AS panels have previously been identified in exosome proteomes of human-origin samples, analysed by various proteomic methods including mass spectrometry. PANTHER software was used to assess the subcellular localization of the proteins. Proteins in the LG panel were found to be distributed among cell components as follows: cell part (57%), macromolecular complex (28.6%) and membrane (14.3%), while proteins in the AS panel are distributed among the cell part (50%), macromolecular complex (25%) and organelle (25%). Phobius and SignalIP software were used to identify which proteins are classified as ‘secreted’ proteins. It is likely that such proteins would have a more prominent role in signaling activity and, importantly, would also be likely to be secreted into the blood or urine meaning that they could be measurable in patient biofluids. This is an important consideration if planning to further verify potentially important protein expression changes in actual human samples. Five proteins in the LG panel were identified as secreted, while 13 secreted proteins were identified in the AS panel (Table 3 and Table 4).

**Figure 5:**
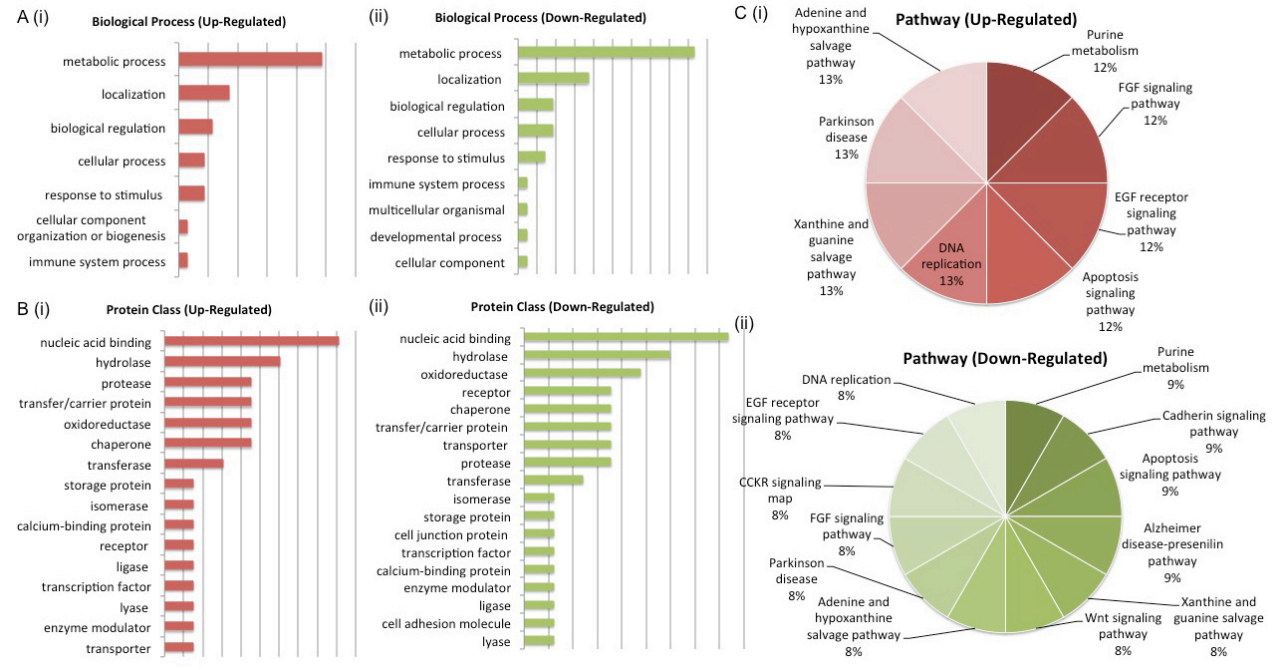
Biological Interrogation of Low Glucose-Associated Proteins of Interest PANTHER anther analysis was performed on proteins selected based on their association with androgen sensitivity in this study. The most de-regulated biological process was that of metabolism (A (i) - (ii)). Protein classes associated with cell signaling activity were represented by both up and down-regulated proteins of interest (B (i) - (ii)). Pathways associated with cancer progression were represented by both up and down-regulated protiens of interest (C (i) - (ii)).

**Figure 6:**
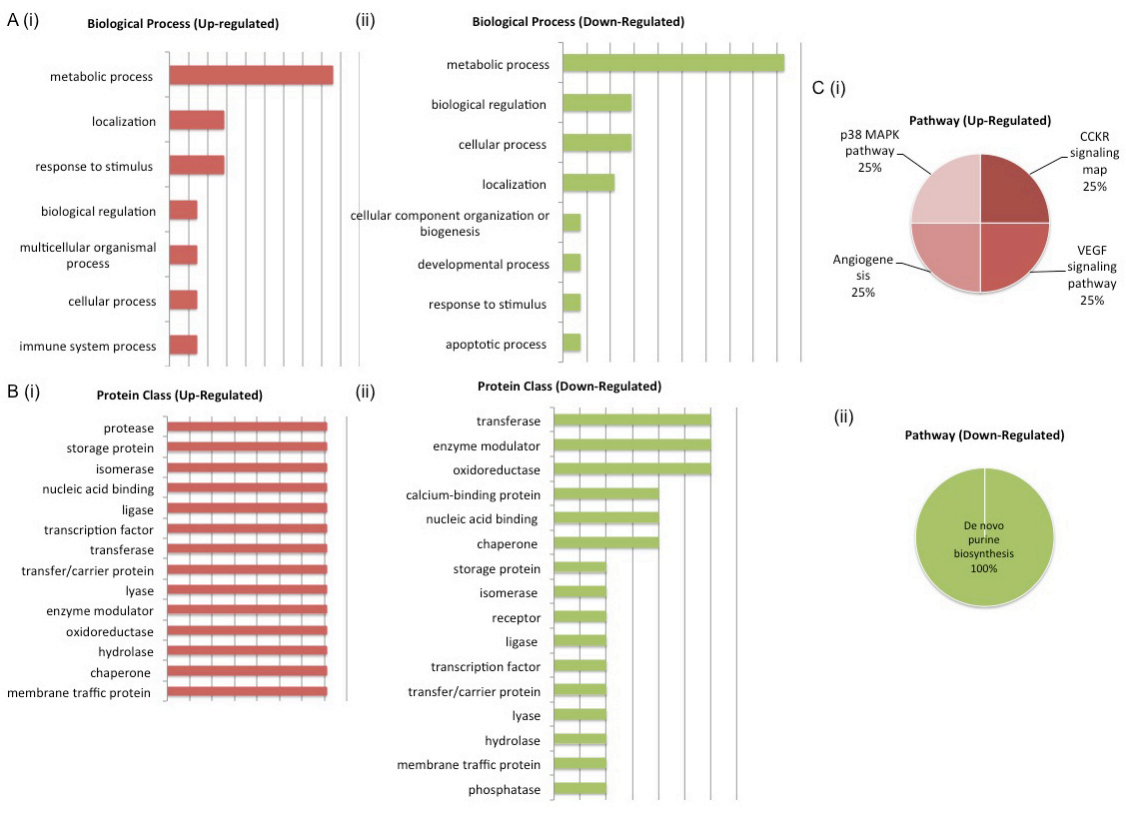
Biological Interrogation of Proteins of Interest Associated with Androgen Sensitivity PANTHER analysis was performed on proteins selected based on their association with androgen sensitivity in this study. Both up and down-regulated protiens mapped to similar biological processes, protein classes and pathways. The most de-regulated biological process was that of metabolism (A (i) - (ii)). Protein classes associated with cell signaling activity were represented by both up and down-regulated proteins of interest (B (i) - (ii)). Pathways associated with cancer progression - p38 MAPK, CCKR signaling, Angiogenesis and VEGF signaling - were most upregulated (C (i) - (ii)).

PANTHER analysis of the two protein panels also highlighted the pathways and biological processes associated with both up-regulated and down-regulated proteins in each panel. For the LG panel, up-regulated proteins mapped to the FGF signaling, EGF receptor signaling, apoptosis signaling, DNA replication, xanthine and guanine salvage, Parkinson disease, adenine and hypoxanthine salvage and purine metabolism pathways. Down regulated proteins mapped to a greater number of pathways, some of which include wnt signaling, CCKR signaling and DNA replication. Again, the biological processes associated with deregulated proteins include metabolic process, localization and biological regulation (Figure 5). For the AS panel up-regulated proteins mapped to the p38 MAPK, CCKR, angiogenesis and VEGF signaling pathways while down-regulated proteins mapped to the de novo purine biosynthesis pathway. De-regulated biological processes for both up and down regulated pathways included the metabolic process, biological regulation, localization and cellular process (Figure 6). Biological interrogation of proteomic data in this way means that efforts to verify observations made from LC-MS/MS analysis can be inclusive of all proteins that may have true biological and/or clinical significance.

### External validation of selected proteins of interest

**Figure 7:**
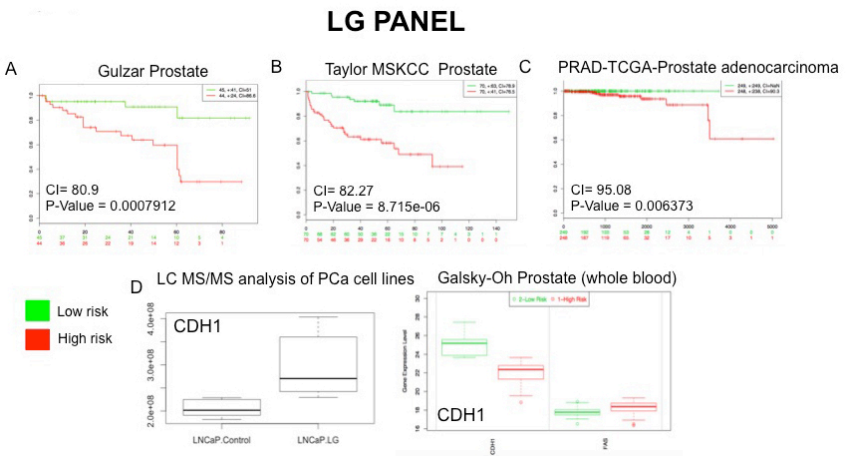
External Validation of LG Panel The SurvExpress bioinformatics resrouce was used to assess the potential clinical utility of proteins in the LG panel. Data from prostate cancer databases which contained data on the full panel of LG proteins was used to assess prognostic value of associated gene expression patterns between high and low risk PCa patients **A**.-**C**. Whole blood gene sequencing data from the Galsky-Oh database validated expression changes observed for the protein CDH1 following unbiased LC-MS/MS analysis of PCa cell lines **D**.

**Figure 8:**
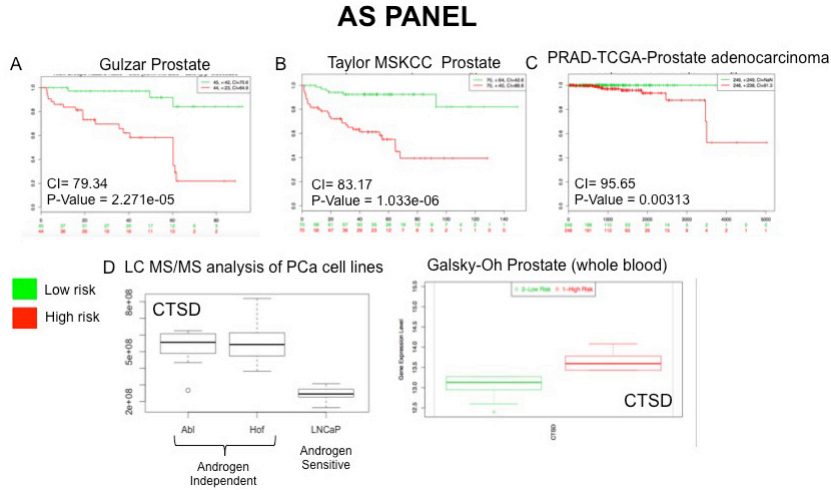
External Validation of AS Panel The SurvExpress bioinformatics resrouce was used to assess the potential clinical utility of proteins in the AS panel. Data from prostate cancer databases which contained data on the full panel of AS proteins was used to assess prognostic value of associated gene expression patterns between high and low risk PCa patients **A**.-**C**. Whole blood gene sequencing data from the Galsky-Oh database validated expression changes observed for the protein CTSD following unbiased LC-MS/MS analysis of PCa cell lines **D**.

To evaluate the potential prognostic capabilities of the LG and AS protein panels for PCa progression the proteins were further analysed using SurvExpress [27]. The SurvExpress bioinformatics resource includes data from 8 prostate cancer datasets containing a total of 1723 samples inclusive of both blood and tissue samples. For this study we availed of the datasets that contained a minimum of 30 samples, allowing validation of the protein panels in 6 clinical datasets with 1,673 samples (Table 5). For both the LG and AS panels, all proteins matched to genes measured in 3 of the 6 PCa datasets - Taylor MSKCC prostate (140 samples) [28], Gulzar Prostate (98 samples) [29] and PRAD-TCGA-Prostate adenocarcinoma (497 samples) [30]. When searched against these databases, the combined list of selected proteins identified in this study gave an average concordance index (CI) of 86.08 and 86.05 for the LG and AS panels, respectfully (Table 5). It is notable that across all 6 datasets, the highest CI and AUC values were obtained with the combination of all proteins in each panel as opposed to a subset of proteins (Table 5). The Galsky-Oh prostate cancer dataset was of special interest as the data was acquired from profiling of whole blood samples from patients with CRPC. Analysis of selected proteins identified in our study indicates a strong association between the proteins ‘cadherin 1’ (LG panel) and ‘isocitrate dehydrogenase’ (AS panel) with CRPC based on data acquired from the study by Galsky and colleagues [31]. Moreover, the expression changes observed are consistent with that observed from analysis of the LC-MS/MS data acquired in this study (Figures 7 and 8). This preliminary validation has therefore shown that, although a cell line model was used for this study, the proteins identified as significant are indeed of clinical relevance and, importantly, are measurable in both tissue and blood samples from patients. Moreover, observations on the CI and AUC (based on SurvExpress analysis, Table 5) indicate that multiplexed measurement of panels of proteins offers much greater predictive power for disease risk, as opposed to individual protein measurements.

**Table 5:** Prosate Cancer Databases for external validation of selected proteins

**Database**	**Samples**	**Clinical Data**	**Details**	**Matching Genes (LG)**	**CI (LG)**	**Survival ROC (LG)**	**Matching Genes (AS)**	**CI (AS)**	**Survival ROC (AS)**
**Taylor MSKCC Prostate**	140	Recurrence, Gleason, Stage	Concordant assessment of DNA copy number, mRNA expression, and focused exon resequencing in 218 prostate cancer tumors	31/31	82.27	0.85	36/36	83.17	0.82
**Galsky Oh - Prostate - GSE45705**	61	Survival	qPCR profiling of whole blood from patients with castration-resistant prostate cancer	2/31	52.92	0.44	1/36	53.86	0.65
**Sboner Rubin Prostate GSE16560**	281	Gleason	cDNA-mediated gene expression profiling on formalin-fixed paraffin-embedded transurethral resection of prostate (TURP) samples	22/31	67.49	0.78	31/36	63.39	0.67
**Gulzar-Prostate-GSE40272**	98	Recurrence	Gene-expression profiling of prostate tumors	31/31	80.90	0.93	36/36	79.34	0.82
**Kollmeyer-Jenkins Prostate GSE10645-GPL5858**	596	Survival, Age, PSA, Stage, Grade	Gene expression microarray of tumors using RNA from archival FFPE tissue	2/31	55.89		7/36	69.92	0.76
**PRAD - TCGA - Prostate adenocarcinoma June 2016**	497	Survival	Gene-expression profiling of prostate tumors	31/31	95.08	0.87	36/36	95.65	0.88

LG = Low Glucose; CI = Concordance index; ROC = Receiver operating characteristic; AS = Androgen Sensitivity

### MRM design for evaluation of selected proteins of interest

MRM assays for all selected proteins (69 in total) were designed using Skyline (version 3.5) software. SpectrumMill (version) software was used to process the raw LC-MS/MS data and generate spectral libraries to support selection of peptides and transitions in the Skyline software. Appropriate proteotypic peptide selection is crucial to the MRM assay development process. The mass spectrometry signal response of different tryptic peptides from the same protein can vary by as much as 100-fold in intensity so it is therefore imperative to select peptides with favorable mass spectrometry properties for optimal assay sensitivity and reproducibility [32, 33]. Proteotypic peptides deemed to have favourable mass spectrometry properties were selected based on criteria outlined in the materials and methods section. Online tools such as Peptide Selector, SRM Atlas and Panorama were also used to prioritise peptide selection towards peptides for which MRM assay parameters had previously been optimized. It was important to verify protein changes based on the measurement of peptides that would have led to the identification of target proteins in the first place. Outputs from Andromeda processing of the raw LC-MS/MS data gave information on the label-free quantification (LFQ) intensity of all measured peptides. One-way ANOVA analysis of the peptide dataset provided a critical value of 1.82. A fold change greater or less than 1.82 could therefore be considered a statistically significant change in expression. Peptides showing a fold change greater than 1.82 or less than -1.82 between androgen independent and androgen sensitive cell lines, or within cell lines as result of treatment with low glucose were deemed ‘significant’ and therefore prioritized for MRM assay development. As a means of predicting which peptides would be detectable in the triple quadrupole mass spectrometer (QqQ) (Agilent 6490), peptides that had previously been identified in the similarly designed Q-Tof mass spectrometer (6550) were given priority. Implementation of such criteria ensures that efforts to verify changes in protein expression by MRM are based on peptide measurements that actually reflect protein changes observed in the discovery phase and are likely to provide high quality, reproducible data. Resulting MRM transition lists for the LG and AS panels are shown in Supplementary data .3 and .4, respectfully.

In order to confirm that changes in protein expression between samples are as result of experimental conditions it is important to normalize the data based on total protein concentration of each analytical sample. As such MRM assays were also designed for measurement of ‘house-keeping’ proteins that should be consistently expressed across all cell lines irrespective of treatment conditions. The LC-MS/MS data from analysis of the PCa cell lines was used to identify proteins identified in all cell lines under both control and low glucose conditions that showed no change in expression (CV ≤ 20%) across all individual samples. These were cross-referenced against a publicly available house-keeping gene database [34] leading to the identification of 12 ‘house-keeping’ proteins - THRAP3, RPL10A, SNRNP200, TOP1, APEH, PCB1, CBX3, TIMM44, DHX15, SLC25A5, PARK7 and HSPA4. MRM assays have been designed for these house-keeping proteins in the same manner as described for proteins in the LG and AS panels.

## DISCUSSION

Thoughtful and well-performed proteomic analysis studies have a very important role in this technology-driven discipline. In the past, progress of significant findings has been limited by the fact that such analysis is difficult to perform such that the findings are robust and generally applicable [35]. In this study, which was strengthened greatly through the use of biological, sample and technical replicates, a comprehensive dataset ( > 3,000 confidently identified proteins) was acquired for androgen sensitive and androgen independent cell lines under ‘normal’ and ‘low glucose’ conditions. As well as profiling the effects of low glucose conditions on protein expression, we were also able to identify discriminatory protein features of androgen sensitive and androgen independent PCa.

It was observed from these data that low glucose conditions had a profound effect on protein expression in both the androgen sensitive and androgen independent cell lines, with between 30-50 and 60-80 significantly differentially expressed proteins identified in each cell line at the 24 hour and 48 hour time points, respectfully (Figure 3). The protein serotransferrin (TF) was consistently significantly up regulated in all cell lines at both time points as result of low glucose conditions, however, is considered to be of limited use as a cancer specific biomarker. Other notable proteins that were found to be significantly up regulated following growth under low glucose conditions were lactate dehydrogenase A and B (LDHA and LDHB). Lactose dehydrogenase (LDH) is known to play a key role in aerobic glycolysis in cancer cells since it can provide an alternative source of NAD+ in the absence of mitochondrial oxidation. This modification of the tumor microenvornment confers several advantages to tumours including favouring invasion and suppressing anti-cancer immune effectors [36]. LDHA has been implicated in the pathogenesis and progression of many cancer types and recent proteomic studies have also shown LDHB to be strikingly increased in cancer cells [37]. Already a number of therapeutic drugs have been developed to target LDH-A in order to interfere with tumour growth and invasiveness [38, 39] while more recent research also suggests that LDH-B would be a viable target to attenuate cancer progression [40]. In addition to the lactate dehyrogenases, the protein fatty acid synthase (FASN) - a key contributor to the Warburg effect - was significantly up-regulated as result of low glucose conditions in the LNCaP-abl cell line. FASN is one of the key enzymes involved in de novo long-chain fatty acid synthesis, which cancer cells rely on in order to meet their markedly increased demands for membrane and energy production and protein synthesis [41]. Early up regulation of FASN in precursor lesions may therefore represent an obligatory metabolic acquisition in response to the microenvironment of premalignant lesions. As such, the role of FASN in cancer pathogenesis is of significant interest and there are numerous reports which attest to its utility as a therapeutic target [41–44]. Indeed 3-V Biosciences will soon have a phase II clinical trial underway to investigate the use of a FASN targeting agent (TVB-2640) in treatment of ovarian, breast and lung cancer [45].

**Figure 9:**
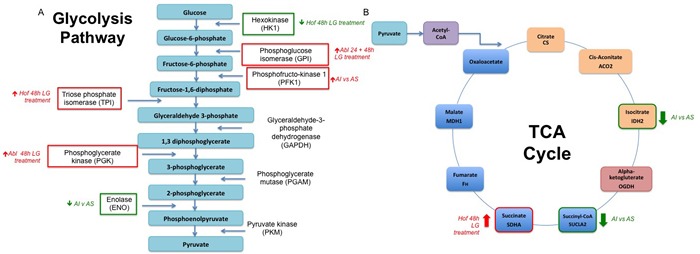
Alterations in Cellular Metabolism in Response to Low Glucose Conditions and Loss in Androgen Sensititivity A number of proteins identified and included as ‘signatures’ for low glucose (LG) conditions or androgen sensitivity (AI v AS) have known roles in regulation of the glycolysis pathway **A**. and TCA cycle **B**. As indicated in figure 9, a number of key enzymes within both pathways are either up or down-regulated in response to low glucose treatment (LG treatment) or between androgen independent (AI) and androgen sensitive (AS) PCa cell lines.

The most discriminatory protein expression changes were observed when comparing the androgen sensitive and androgen independent PCa cell lines (Figure 4). Here it was observed that the lacto dehydrogenase proteins LDHA and LDHB were decreased in the androgen independent cell lines as opposed to the androgen sensitive cell line. On the other hand, a number of proteins associated with glutamine metabolism - GPRS, GDH1 and IDH1 were significantly up regulated in the androgen independent cell line as opposed to the androgen sensitive cell line. The importance of glutamine metabolism for cancer growth and viability has previously been highlighted and the possibility of developing therapies that can exploit glutamine metabolism for therapeutic gain has been explored [46, 47]. The protein nicotinamide phosphoribosyltrensferase (NAMPT) was also up regulated in the androgen independent cell lines. NAMPT is the rate-limiting enzyme in the biosynthesis of NAD+ from nicotinamide, thereby providing cancer cells with one of the key metabolites essential for sustaining energy metabolism [48]. Increased expression of NAMPT has been observed in many cancer types and previous studies have shown that inhibition of NAMPT leads to the attenuation of tumour growth and induction of apoptosis due to NAD+ depletion [49]. In PCa it has been shown that NAMPT knockdown sensitizes PCa cells to oxidative stress caused by chemotherapeutic treatment. Taken together, these studies demonstrate the potential clinical benefit of further exploring the NAMPT pathway for PCa prevention and treatment [49, 50]. Another notable protein found to be increased in expression in the androgen independent cell lines as opposed to the androgen sensitive cell line was the lysosomal enzyme cathepsin D. Secreted cathepsins are suggested to have a role in promoting cellular motility, invasion and angiogenesis by degrading the extracellular matrix [51]. It has indeed been shown that blocking cathepsins by small molecule inhibitors can significantly delay cancer progression in a number of mouse models as well as sensitizing tumours to chemotherapeutic intervention [52]. Overall, a number of proteins with known links to altered metabolism in the tumour microenvironment were found here to be de-regulated as result of low glucose and androgen sensitivity (Figure 9). This is in line with our current understanding of the tumour microenvironment and also contributes further evidence to the role of androgen signaling in promoting the Warburg effect in CRPC. From a clinical perspective, the role for androgen in altering cancer cell metabolism could have implications for the development of therapeutic agents.

In the last number of years the focus of biomarker research has shifted from trying to identify a single analyte to consolidating multiple analytes into biomarker panels/signatures that can be used for clinical diagnostics and patient stratification [53]. The use of MRM is advantageous in this respect as it allows for rapid, high throughput measurement of panels of proteins. Successful studies have previously been undertaken by our group that were focused on the design of MRM assays for the measurement of protein panels with potential prognostic value for PCa and other inflammatory diseases [54–56] Within this dataset there were a number of significantly changing proteins associated with low glucose conditions and/or androgen sensitivity. These were short-listed into panels for subsequent MRM development with priority given to those proteins with previous associations as drug targets or biomarkers. These panels incorporated those proteins that have relevant links to altered metabolism in cancer, indeed PANTHER analysis of all short-listed proteins revealed them to predominantly have roles in the metabolic processes. All proteins in both panels have previously been identified in exosome-based studies, thereby highlighting their potential role as signaling proteins. The clinical significance of selected proteins was verified through meta-analysis of gene array data acquired from studies involving various clinical cohorts. Selected proteins from both the AS and LG panel, which have previously been described as disease biomarkers, were shown to have a significant association with ‘high risk’ PCa in various large-scale clinical studies (Table 5 Figures 7 & 8). Furthermore, a number of proteins from both panels are classified as ‘secreted’ proteins and therefore have potential to be measured non-invasively in patient biofluids. MRM assyas have been designed to measure these proteins (Supplementary data Table S3 and S4) and further experiements to optimize these assays and apply them for verification of protein expression changes are ongoing.

A significant limitation to this study is the difficulty in accurately re-capitulating the tumour microenvironment in a 2D cell culture system. For this study analysis of androgen sensitivity, as well as the effects of low glucose conditions, was undertaken on a consistent genetic background using the androgen sesnsitve LNCaP cell line and two of its androgen independent derivative cell lines - LNCaP-abl (Abl) and LNCaP-abl-Hof (Hof). The Abl and Hof cell lines were initially established as tumour models that bear close resemblance to the *in vivo* conditions in which patients who receive androgen ablation therapy subsequently develop resistance [22]. Thus we consider them a satisfactory model for development of CRPC. Our group has previously applied workflows similar to those described here for proteomic profiling of the PCa tumour microenvironment using laser capture microdissected regions of patient tumour tissue [57]. Reassuringly, there is significant overlap between changing proteins identified in this cell-based study and previous tissue-based studies (data not shown), thus providing confidence that our cell line system represents a faithful model of the *in vivo* tumour microenvironment. Indeed, our unbiased label-free approach highlighted a number of protein changes that are in keeping with what has previously been reported with regards to the Warburg effect, glutamine metabolism and increased expression/activity of lactose dehydrogenases and lysosomal enzymes in PCa [36, 37, 58]. There is currently a vast repertoire of well-annotated PCa cell lines available for PCa research and it would be interesting to compare the data described here with data from alternative androgen-insensitive cell lines - e.g. PC-3 or DU145 - which are also used to model aggressive PCa [23, 24].

The large dataset acquired in this study has so far allowed us to (i) gain further biological insight into the PCa tumor microenvironment, (ii) identify potential protein biomarkers that may be indicative of treatment resistant PCa (CPRC) and/or altered metabolism and (iii) identify potential drug targets for therapeutic intervention in PCa. Appropriate verification of the significance of these proteins will be important if we are to extract clinically meaningful benefit from this dataset. As such, we have described here a thorough strategy which ensures that future verification studies will be undertaken using MRM assays that (i) are based on the measurement of proteotypic peptides that accurately reflect the changes observed at the protein level and can be routinely detected on a QqQ and (ii) allow for appropriate normalization of the MRM data based on the total protein content of individual samples. Overall, we believe that these data suggest that this strategy supports identification of protein biomarkers of PCa progression under adverse conditions as well as the identification of novel therapeutic targets for aggressive PCa.

## MATERIALS AND METHODS

### Cell culture

The PCa cell lines, LNCaP, LNCaP-abl (abl) and LNCaP-abl-Hof (Hof) were gifted to the Irish Prostate Cancer Research Consortium, Dublin, Ireland from the laboratory of Professor Helmut Klocker (Department of Urology, University of Innsbruck, Austria). Culturing of the above cell lines was conducted in a class II laminar flow cabinet. Cells were maintained in T175cm2 flasks with ventilation (Starsted) in a 5% CO2 humidified atmosphere at 37oC. LNCaP cells were maintained in Advance RPMI 1640 media (GIBCO Life Technologies) and supplemented with 10% foetal calf serum (FCS) (Sigma-Aldrich), 2μM/ml L-Glutamine (GIBCO Life Technologies), 50 unit/ml Penicillin and 50μg/ml Streptomycin (GIBICO Life Technologies). Abl and Hof cells were maintained in Advance RPMI 1640 media supplemented with 10% charcoal stripped FCS (Sigma-Aldrich), 2μM/ml L-Glutamine (GIBCO Life Technologies), 50 unit/ml Penicillin and 50μg/ml Streptomycin (GIBICO Life Technologies). For the three cell lines media was changed every 3-4 days.

### Simulation of low glucose conditions in PCa cell lines

LNCaP, Abl and Hof cell lines were seeded into 10 cm^2^ culture dishes and grown to 70-80% confluence. To induce low glucose conditions, media was removed and replaced with RPMI (-Glucose) media supplemented with 10% FCS (LNCaP) or CSS (abl, Hof) and 1% Penicillin/Streptomycin. Cells were incubated in either low glucose or standard media for either 24 or 48 hours prior to cell lysis. For each cell line, 3 biological replicates were performed for each time point.

### Sample preparation for nLC-MS/MS analysis

Adherent cells were washed twice with ice-cold PBS and removed from cell culture plates by scraping. Cells were transferred into eppendorf tubes and spun for 5 min at 3,000 *g* at 4°C. PBS was removed and cell pellet was lysed by sonication in 100 μl 1% sodium dodecyl sulfate (SDS; Sigma Aldrich). Samples were heated at 95°C for 5 min to encourage denaturation, and subsequently centrifuged at 14,000 *g* for 10 min at 4°C to remove cell debris. Protein concentration of cell lystates was measured by BCA assay according to manufacturer's instructions (Pierce). Whole cell lysates were prepared for nLC-MS/MS analysis according to the filter aided sample preparation (FASP) method as described by Wisniewski et al (AK 49). Briefly, 50 μg cell lysate proteins were reduced through boiling (95°C for 5 min) with DTT in a final concentration of 0.1 M. 200 μl UA buffer (8 M Urea, 0.1 M Tris-HCL, pH 8.5) was added to each sample, and samples were transferred to 30,000 MQCO Vivacon 500 spin filters (Sartorious) and centrifuged at 14,000 *g* for 40 min, 21°C. Bound proteins were alkylated through 5 min incubation of spin filters in 0.05 M iodoacetamide (IAA) followed by centrifugation at 14,000 *g* for 30 min, 21°C. Spin filter membranes were then washed three times through addition of UB (8 M Urea, 0.1 M Tris/HCL, pH 8.0) and centrifugation at 14,000 *g* for 40 min, 21°C. For maximum protein identifications, sample protein was digested with both Lys-C (Wako Chemicals GmbH) and Trypsin (Promega) enzymes. Proteins were initially digested overnight with Lys-C (enzyme: substrate 1:50) in a wet chamber. Digestion was completed by a 3-hour incubation with Trypsin (enzyme: substrate 1:100) in a thermomixer set to 37°C, 600 rpm. Digestion was stopped by acidification of samples through addition of trifluoroacetic acid (TFA) to a final concentration of 1%. Peptide material from digested cell lysates were purified using C18 resin ZipTips^®^ (Millipore). Each ZipTip contains C18 resin packed into a 10 μl pipette tip with a loading capacity of 5 μg protein/peptide for tip. This allows for purification of peptide material of molecular weight between 0-50 kDa. For purification of cellular peptides, the C18 resin was activated with 10 μl acetonitrile (x10). The resin was then equilibrated by pipetting 10 μl 0.5% trifluoroacetic acid (TFA) (x10). Peptides were then bound to the resin by pipetting 15 μl of digested sample through the resin (x10). Bound peptides were eluted into fresh eppendorfs in 25 μl Elution Buffer [70% acetonitrile, 0.1% TFA] (x2). This process was repeated four times for each sample to ensure maximum yield of purified peptide for nLC-MS/MS analysis. Eluted peptides were dried down under vacuum for approximately 1 hour at 30 °C and re-suspended in 30 μl Buffer A [3% CAN, 0.1% formic acid] to allow for ≈3 μg peptide per 5 μl injection on the Q-Exactive mass spectrometer.

### nLC-MS/MS analysis

Samples were analysed by nano-flow reverse phase LC using a Q-Exactive mass spectrometer connected online to an Ultimate Ultra3000 chromatography system (both Thermo Fisher Scientific) as described [59]. Briefly, dried peptides were reconstituted in 0.01 % TFA and 5 µl of each sample were loaded onto an in house prepared analytical column (150 mm length, 75 µm inside diameter) packed in house with 1.9 µm ReprosilAQ C18 (Dr Maisch GmbH). Tryptic peptides were separated using a 130-minute linear gradient from 4 % to 32 % acetonitrile at a flow rate of 250 nl/min. The mass spectrometer was operated in data-dependent acquisition mode with a top-12 MS/MS scanning approach. For protein label-free identification and quantification, tandem mass spectra and peptide fragments of the 12 most abundant peaks were acquired in the linear ion trap by peptide fragmentation using higher energy collisional dissociation (HCD). A 2300 V potential was applied to column with a capillary temperature of 320 °C. Samples generated for each time point were analysed in two separate experimental runs in a randomized order. To monitor the technical reproducibility of the experiment, sample replicates (SR) and technical replicates (TR) - generated from pooled sample material post-digest and post-peptide ‘clean up’, respectfully - were analysed at the beginning, middle and end of the experimental run order for both the 24 and 48 hour time points.

### Data processing and statistical analysis

PEAKS (version 7) software was used to determine the number of peptides and proteins identified in each sample. Files generated from nLC-MS analysis (.d) were directly uploaded onto the PEAKS software and database searching was performed using ‘HumanUniprot’ database [39,704 protein sequences] (downloaded 01/11/2013) with the following search parameters applied: enzyme: trypsin, maximum missed cleavages: 2, species: Homo Sapiens, variable modifications: oxidation methionine, 4-hydroxynonenal (4-HNE), lysine acetylation at N and C termini, amidation, ammonia loss at N and C termini, precursor ion tolerance: 10 ppm, product ion tolerance: 0-3 and maximum variable modifciations per peptide: 3. The false discovery rate (FDR) was set to 0.1%. The raw LC-MS/MS data was then processed using MaxQuant computational proteomics platform version 1.4.1.2. Raw files were directly imported into the software and protein identifications were generated by processing the data through the in-built Andromeda search engine matched, against the Uniprot/Swissprot database [40.452 protein sequences] (downloaded 29/07/2014) with FDR set to 0.5%. Additional search parameters were as follows, enzyme: trypsin, allow up to two missed cleavages, species: Homo sapiens, fixed modification: carbimidomethylated cysteine, variable modification: oxidation methionine, minimum peptide length: 7 amino acids. The precursor mass tolerance window was set to 6 ppm and product mass tolerance was 20 ppm. A minimum of 2 peptides was required to confirm protein identification. This search provided a full list (.txt format) of peptide and protein identifications along with their respective label-free quantitation (LFQ) intensities. Further data processing and statistical analysis was performed by uploading this .txt file into Perseus (version 1.5.0.15), provided as part of the MaxQuant software solution package (www.maxquant.org). Here, the data were filtered to remove all protein contaminants, reverse-phase proteins, and those proteins only identified by site - an automated data processing feature of the Perseus software. The software was then used for imputation, normalization, PCA, hierarchical clustering and statistical analysis of the data. Briefly, data for analysis was transformed to a log2 scale and missing values were imputed with constant values to allow the assignment of the presence or absence of proteins between conditions. All statistical *t*-tests, to distinguish proteins differentially expressed between conditions, were performed with a p value threshold of 0.05. For hierarchical clustering, Euclidean distances were applied using logarithmised intensities after z-score normalisation of the statistically significant data. Principal component analysis was undertaken on logarithmised values only. Differentially expressed proteins were further analysed using Ingenuity Pathway Analysis Knowledge Database (Ingenuity Systems) to map statistically significant proteins to the pathways and biological processes in which they were enriched.

### MRM design and data analysis

Candidate proteins for MRM verification were selected from the full list of significantly changing proteins based on their %CV values. The %CVs were calculated using data from the SR and TR samples as these were analysed as a means of removing any technical bias from the LC-MS/MS data acquired of the biological replicates. In total, 157 of the proteins that were significantly changed in expression as result of low glucose conditions with CV ≤ 20% were identified. This list was further refined to include only those proteins with previous association as a biomarker or therapeutic target. MRM assay design was performed using Skyline software (MacCoss laboratory, Washington DC version 3.5). Raw LC-MS/MS data from Q-Exactive analysis of the experimental lystates was used to generate spectral librraies in Skyline. Proteotypic peptides with associated spectral library data were selected for all proteins of interest according to the following criteria: no missed cleavages or ‘ragged ends’, sequence length between 8-25 amino acids. Where possible, peptides sequences with reactive (C) or methionine (M) residues were avoided. Where possible, peptides that were identified in the discovery (nLC-MS/MS) analysis were prioritized. SRM atlas - which acts as a public repository of developed MRM assays - was also used to guide selection of proteotypic peptides. Between 2 and 3 peptides were selected for each protein initially with 4 - 5 transitions selected per peptide. For all transitions, precursor ions with a charge state of 2 were selected with product ions limited to singly charged y ions. In order to minimize potential interferences, ions with m/z close to the precursor ions or an m/z > 1,000 were excluded. A working MRM was determined based on the dot product ( ≥ 0.85), quality of peak shape (no fronting or tailing, minimum of 10 data points collected per peak) and coefficient of variance (CV < 20%). The MRM assays were developed using a pooled sample of all digested lysate from the three cell lines (low glucose and control) at both time points. MRM analysis was undertaken on an Agilent 6490 QqQ mass spectrometer, with 5 sample replicates analysed for each round of assay optimisation. Raw data (.d) was uploaded directly into Skyline for analysis. Visual inspection aided manual interrogation of peptide peaks to ensure that the correct peak was identified for each target peptide. Subsequently, a report was exported from Skyline containing information on the area, full width half maximum (FWHM), retention time and peak rank for all transitions.

## SUPPLEMENTARY MATERIALS FIGURES AND TABLES




