# Supplementary material for: Probing the prostate tumour microenvironment I: impact of glucose deprivation on a cell model of prostate cancer progression
Source: Oncotarget. 2017 Jan 12;8(9):14374–94. doi: 10.18632/oncotarget.14605 (PMC5362412; doi:10.18632/oncotarget.14605)
Supplement: Supplementary file 1 [file oncotarget-08-14374-s001.pdf]

Probing the prostate tumour microenvironment I: impact of glucose deprivation on a cell model of prostate cancer progression

Supplementary Material

Supplementary Data Table S1: Significantly Changing Proteins As Result of Low Glucose Conditions.
For Data Table S1, please see the attached file

Supplementary Data Table S2: Common Significantly Changing Proteins Between Androgen Sensitive and Androgen Independent Cell Lines.
For Data Table S2, please see the attached file

Supplementary Data Table S3: Transition List for Hx Panel.
For Data Table S3, please see the attached file

Supplementary Data Table S4: Transition List for AS Panel
For Data Table S4, please see the attached file
