# Supplementary material for: Probing the prostate tumour microenvironment I: impact of glucose deprivation on a cell model of prostate cancer progression
Source: Oncotarget. 2017 Jan 12;8(9):14374–94. doi: 10.18632/oncotarget.14605 (PMC5362412; doi:10.18632/oncotarget.14605)
Supplement: Supplementary file 2 [file oncotarget-08-14374-s002.docx]

**Table S.1. Significantly Changing Proteins As Result of Low Glucose Conditions**

| 24h | | | 48h | | |
| --- | --- | --- | --- | --- | --- |
| Majority protein IDs | Gene names | t-test Difference | Majority protein IDs | Gene names | t-test Difference |
| LNCaP | | | | | |
| O00161-2 | SNAP23 | -1.97 | O14745 | SLC9A3R1 | 1.16 |
| O00487 | PSMD14 | -1.26 | O14828 | SCAMP3 | -1.73 |
| O00754-2 | MAN2B1 | -1.25 | O43395 | PRPF3 | -1.64 |
| O14828 | SCAMP3 | -1.68 | O43396 | TXNL1 | 1.33 |
| O15347 | HMGB3 | -1.50 | O43707 | ACTN4 | 1.76 |
| O43678 | NDUFA2 | -0.88 | O75153 | CLUH | 1.35 |
| O43681 | ASNA1 | -1.33 | O75396 | SEC22B | -1.41 |
| O60664-4 | PLIN3 | -1.19 | O94905 | ERLIN2 | -2.28 |
| O60716-21 | CTNND1 | -1.89 | P00491 | PNP | 1.63 |
| P00338 | LDHA | 0.87 | P02786 | TFRC | -1.91 |
| P02786 | TFRC | -1.45 | P02787 | TF | 2.68 |
| P02787 | TF | 2.18 | P04040 | CAT | -1.80 |
| P12830 | CDH1 | -1.67 | P05026-2 | ATP1B1 | -1.49 |
| P23284 | PPIB | 1.06 | P06748-3 | NPM1 | -1.55 |
| P25205 | MCM3 | 1.22 | P07602 | PSAP | -1.30 |
| P26885 | FKBP2 | 0.84 | P07900 | HSP90AA1 | 1.52 |
| P28838-2 | LAP3 | -1.89 | P07910-2 | HNRNPC | -1.71 |
| P30101 | PDIA3 | 0.45 | P10515 | DLAT | -1.48 |
| P30153 | PPP2R1A | -0.83 | P10809 | HSPD1 | -1.49 |
| P35580 | MYH10 | -0.64 | P11171-6 | EPB41 | -0.77 |
| P38646 | HSPA9 | 0.80 | P11388 | TOP2A | -0.66 |
| P38919 | EIF4A3 | 1.96 | P11717 | IGF2R | -1.82 |
| P46777 | RPL5 | 1.85 | P12956 | XRCC6 | 0.83 |
| P46782 | RPS5 | 1.46 | P13995 | MTHFD2 | -1.53 |
| P46940 | IQGAP1 | -0.87 | P14735 | IDE | 1.18 |
| P51858 | HDGF | 1.07 | P21291 | CSRP1 | 1.18 |
| P55145 | MANF | 1.07 | P22694-4 | PRKACB | -1.65 |
| P62899 | RPL31 | 1.60 | P24539 | ATP5F1 | -0.90 |
| P62910 | RPL32 | 1.75 | P25705 | ATP5A1 | -1.68 |
| P83731 | RPL24 | 2.02 | P30086 | PEBP1 | 1.35 |
| Q00535 | CDK5 | -1.61 | P31937 | HIBADH | -1.92 |
| Q01130 | SRSF2 | 1.80 | P35580 | MYH10 | 0.54 |
| Q01780-2 | EXOSC10 | -1.30 | P39656 | DDOST | -0.67 |
| Q02543 | RPL18A | 1.60 | P40939 | HADHA | -1.28 |
| Q13907 | IDI1 | -1.39 | P45880 | VDAC2 | -1.13 |
| Q16629-3 | SRSF7 | 1.55 | P47985 | UQCRFS1;UQCRFS1P1 | -0.83 |
| Q3LXA3 | DAK | -0.77 | P51858 | HDGF | 1.74 |
| Q5VWZ2 | LYPLAL1 | 0.30 | P53582 | METAP1 | 1.28 |
| Q6P2E9 | EDC4 | -2.26 | P55060-3 | CSE1L | 0.96 |
| Q6P587 | FAHD1 | -1.39 | P56181-2 |  | -1.51 |
| Q6PI48 | DARS2 | -1.49 | P61313 | RPL15 | 1.32 |
| Q8N163-2 | KIAA1967 | -1.64 | P61586 | RHOA | 1.89 |
| Q92841 | DDX17 | 1.08 | P61916 | NPC2 | -1.82 |
| Q92973-2 | TNPO1 | -2.33 | P62333 | PSMC6 | 1.37 |
| Q96N66 | MBOAT7 | -0.65 | P63010 | AP2B1 | -1.07 |
| Q96PK6 | RBM14 | 1.67 | Q04760-2 | GLO1 | 1.71 |
| Q99460-2 | PSMD1 | -1.48 | Q05639 | EEF1A2 | 0.63 |
| Q99805 | TM9SF2 | -1.52 | Q13155 | AIMP2 | -0.77 |
| Q9BSJ8 | ESYT1 | -1.77 | Q13185 | CBX3 | -1.10 |
| Q9H8H3 | METTL7A | -1.29 | Q14739 | LBR | -0.79 |
| Q9H9J2 | MRPL44 | -1.75 | Q14789 | GOLGB1 | -1.86 |
| Q9NX58 | LYAR | 1.66 | Q15185 | PTGES3 | 1.58 |
| Q9NZL9 | MAT2B | -1.06 | Q16891-2 | IMMT | -1.29 |
| Q9UHE8 | STEAP1 | 1.71 | Q5BKZ1 | ZNF326 | 1.49 |
| Q9Y6Q5 | AP1M2 | -1.18 | Q5JRA6 | MIA3 | -1.11 |
|  |  |  | Q8IX12-2 | CCAR1 | -2.30 |
|  |  |  | Q8TCS8 | PNPT1 | -1.69 |
|  |  |  | Q8TCT9-5 | HM13 | -1.04 |
|  |  |  | Q92841 | DDX17 | -1.33 |
|  |  |  | Q92900-2 | UPF1 | 1.18 |
|  |  |  | Q96KB5 | PBK | 1.49 |
|  |  |  | Q96PK6 | RBM14 | -1.15 |
|  |  |  | Q99653 | CHP1 | 0.84 |
|  |  |  | Q9BSJ8 | ESYT1 | -1.87 |
|  |  |  | Q9BWS9-3 | CHID1 | -2.00 |
|  |  |  | Q9H2P0 | ADNP | -1.70 |
|  |  |  | Q9HB71 | CACYBP | 1.61 |
|  |  |  | Q9UPQ0-10 | LIMCH1 | -1.10 |
|  |  |  | Q9Y5S9 | RBM8A | 1.39 |
|  |  |  | Q9Y6E2 | BZW2 | 2.32 |
|  |  |  | Q9Y6Y8 | SEC23IP | 0.84 |
| Abl | | | | | |
| O14929 | HAT1 | 2.28 | O14818 | PSMA7 | 1.00 |
| O15212 | PFDN6 | -1.13 | O75083 | WDR1 | 2.50 |
| O60313 | OPA1 | -0.84 | O75347 | TBCA | 1.78 |
| O75746 | SLC25A12 | -1.75 | O76003 | GLRX3 | 1.03 |
| O95197-3 | RTN3 | 0.38 | O95140 | MFN2 | -1.10 |
| O95298-2 | NDUFC2;NDUFC2-KCTD14 | 0.75 | O95347 | SMC2 | 2.34 |
| P00505 | GOT2 | -2.25 | O95573 | ACSL3 | -0.25 |
| P02786 | TFRC | -1.47 | P00338 | LDHA | 1.00 |
| P02787 | TF | 2.01 | P00558 | PGK1 | 1.10 |
| P02794 | FTH1 | 0.91 | P02787 | TF | 1.33 |
| P05388 | RPLP0;RPLP0P6 | -0.85 | P05026-2 | ATP1B1 | -1.12 |
| P06744 | GPI | 1.45 | P06744 | GPI | 0.97 |
| P08238 | HSP90AB1 | 1.04 | P07195 | LDHB | 0.28 |
| P08754 | GNAI3 | 0.87 | P07602 | PSAP | -1.90 |
| P10515 | DLAT | -1.13 | P08758 | ANXA5 | 0.98 |
| P11142 | HSPA8 | 0.93 | P10644 | PRKAR1A | 2.25 |
| P12081-4 | HARS | 0.75 | P11233 | RALA | -2.47 |
| P17987 | TCP1 | 1.18 | P12004 | PCNA | 1.44 |
| P20645 | M6PR | -0.66 | P13639 | EEF2 | 1.24 |
| P25787 | PSMA2 | -0.92 | P13693 | TPT1 | 1.54 |
| P25788-2 | PSMA3 | 0.48 | P17987 | TCP1 | 0.80 |
| P26599 | PTBP1 | -0.98 | P18124 | RPL7 | 0.80 |
| P26639 | TARS | 0.90 | P20340-2 | RAB6A | -1.74 |
| P31942-2 | HNRNPH3 | 1.52 | P23381 | WARS | 1.41 |
| P31948 | STIP1 | 1.12 | P25205 | MCM3 | 1.07 |
| P33316-2 | DUT | 1.19 | P26599 | PTBP1 | -0.94 |
| P35232 | PHB | -1.13 | P26639 | TARS | 1.32 |
| P49790 | NUP153 | 1.08 | P30050 | RPL12 | -1.77 |
| P50570-3 | DNM2 | 1.13 | P31948 | STIP1 | 1.33 |
| P51149 | RAB7A | -1.37 | P33121 | ACSL1 | -0.29 |
| P51970 | NDUFA8 | -0.53 | P35221 | CTNNA1 | -1.50 |
| P61160 | ACTR2 | 1.10 | P35606 | COPB2 | 1.61 |
| P61916 | NPC2 | -1.78 | P35659 | DEK | 1.82 |
| P78330 | PSPH | 0.46 | P43487 | RANBP1 | 1.43 |
| P83731 | RPL24 | 1.63 | P45973 | CBX5 | 1.59 |
| Q01813 | PFKP | 1.11 | P46781 | RPS9 | 1.69 |
| Q15031 | LARS2 | -1.16 | P49748 | ACADVL | -0.72 |
| Q15691 | MAPRE1 | 1.17 | P52565 | ARHGDIA | 0.75 |
| Q53H12 | AGK | -1.66 | P53007 | SLC25A1 | -1.87 |
| Q7L5N1 | COPS6 | 1.45 | P54136 | RARS | 1.64 |
| Q8IYQ7 | THNSL1 | -1.27 | P61289 | PSME3 | 1.81 |
| Q8TDD1 | DDX54 | 1.49 | P61916 | NPC2 | -1.95 |
| Q8TEM1 | NUP210 | -0.56 | P62249 | RPS16 | 2.30 |
| Q8WVJ2 | NUDCD2 | 1.37 | Q06830 | PRDX1 | 0.70 |
| Q99615-2 | DNAJC7 | 1.25 | Q08945 | SSRP1 | 1.43 |
| Q99733 | NAP1L4 | 1.56 | Q13813 | SPTAN1 | -0.85 |
| Q9BQ52-4 | ELAC2 | 0.95 | Q15102 | PAFAH1B3 | 1.81 |
| Q9HDC9 | APMAP | 0.73 | Q15181 | PPA1 | 1.23 |
| Q9NR56 | MBNL1 | 1.68 | Q16222-3 | UAP1 | 0.63 |
| Q9NZ45 | CISD1 | 1.00 | Q5BKZ1 | ZNF326 | 1.41 |
| Q9UHB9 | SRP68 | 1.27 | Q5JTV8 | TOR1AIP1 | -1.70 |
| Q9UHE8 | STEAP1 | 1.95 | Q92598-2 | HSPH1 | 1.55 |
| Q9UII2 | ATPIF1 | 1.02 | Q92804-2 | TAF15 | 1.66 |
| Q9UL25 | RAB21 | -1.51 | Q9BXJ9 | NAA15 | 2.20 |
| Q9ULV4 | CORO1C | 1.55 | Q9BYC9 | MRPL20 | -1.85 |
| Q9Y277 | VDAC3 | -1.22 | Q9NX63 | CHCHD3 | -0.84 |
| Q9Y320-2 | TMX2 | -0.80 | Q9P2E9-2 | RRBP1 | -0.67 |
|  |  |  | Q9UHD1-2 | CHORDC1 | 1.55 |
|  |  |  | Q9UII2 | ATPIF1 | 1.78 |
|  |  |  | Q9Y617 | PSAT1 | 1.67 |
| Hof | | | | | |
| O00231 | PSMD11 | -0.71 | Q99613 | EIF3C;EIF3CL | 1.00 |
| O15372 | EIF3H | 0.70 | O00232 | PSMD12 | 2.07 |
| O43396 | TXNL1 | 1.30 | O00264 | PGRMC1 | -1.49 |
| O43818 | RRP9 | 0.86 | O00330 | PDHX | -0.98 |
| O60763 | USO1 | 0.98 | O14737 | PDCD5 | 1.52 |
| P02787 | TF | 2.08 | O14980 | XPO1 | 0.67 |
| P10155-3 | TROVE2 | -2.10 | O15372 | EIF3H | 1.99 |
| P10644 | PRKAR1A | -2.04 | O43615 | TIMM44 | -1.37 |
| P11233 | RALA | -2.52 | O43676 | NDUFB3 | -1.44 |
| P12277 | CKB | -0.69 | O60716-21 | CTNND1 | -1.14 |
| P28074 | PSMB5 | -0.94 | O75369-2 | FLNB | 0.50 |
| P29084 | GTF2E2 | -0.62 | O75534-2 | CSDE1 | 0.90 |
| P33991 | MCM4 | 1.09 | O75947 | ATP5H | -1.70 |
| P40938 | RFC3 | 1.30 | P00387-2 | CYB5R3 | 1.26 |
| P42677 | RPS27 | 1.71 | P00491 | PNP | 1.21 |
| P52292 | KPNA2 | 1.52 | P02786 | TFRC | -1.31 |
| P52434 | POLR2H | 0.67 | P02787 | TF | 1.55 |
| P53992 | SEC24C | 1.11 | P05141 | SLC25A5 | -2.33 |
| P55072 | VCP | -0.98 | P06753-2 |  | 1.79 |
| P55786 | NPEPPS | -1.05 | P07099 | EPHX1 | -0.45 |
| P61513 | RPL37A | 0.93 | P07195 | LDHB | 0.30 |
| P62249 | RPS16 | 1.19 | P07602 | PSAP | -2.13 |
| P68400 | CSNK2A1;CSNK2A3 | -1.94 | P07741-2 | APRT | 1.38 |
| P82979 | SARNP | 1.45 | P07910-2 | HNRNPC | -1.67 |
| Q13501 | SQSTM1 | -0.49 | P10644 | PRKAR1A | 1.51 |
| Q14671-4 | PUM1 | 2.48 | P11021 | HSPA5 | 1.50 |
| Q14914-2 | PTGR1 | 1.31 | P11717 | IGF2R | -2.17 |
| Q8TCT9-5 | HM13 | 0.88 | P11766 | ADH5 | 1.95 |
| Q99615-2 | DNAJC7 | 0.95 | P11940 | PABPC1;PABPC3 | 1.40 |
| Q9GZR7 | DDX24 | 1.92 | P12004 | PCNA | 1.45 |
| Q9Y281-3 | CFL2 | 0.59 | P13995 | MTHFD2 | 0.99 |
| Q9Y6Q5 | AP1M2 | -1.38 | P14866 | HNRNPL | -0.89 |
|  |  |  | P15170-2 | GSPT1;GSPT2 | 0.98 |
|  |  |  | P15586 | GNS | -1.30 |
|  |  |  | P19367-4 | HK1 | -1.32 |
|  |  |  | P20042 | EIF2S2 | 1.33 |
|  |  |  | P22570 | FDXR | -0.48 |
|  |  |  | P22695 | UQCRC2 | -0.76 |
|  |  |  | P23381 | WARS | 2.08 |
|  |  |  | P23588 | EIF4B | 1.31 |
|  |  |  | P24752 | ACAT1 | -0.96 |
|  |  |  | P26599 | PTBP1 | -1.46 |
|  |  |  | P26639 | TARS | 1.10 |
|  |  |  | P26640 | VARS | 0.79 |
|  |  |  | P30536 | TSPO | -1.56 |
|  |  |  | P31040 | SDHA | 0.47 |
|  |  |  | P36542-2 | ATP5C1 | -1.28 |
|  |  |  | P36957 | DLST | -0.98 |
|  |  |  | P38606 | ATP6V1A | -1.72 |
|  |  |  | P42704 | LRPPRC | -0.73 |
|  |  |  | P43487 | RANBP1 | 2.14 |
|  |  |  | P46940 | IQGAP1 | -0.41 |
|  |  |  | P49327 | FASN | 1.01 |
|  |  |  | P49588 | AARS | 0.46 |
|  |  |  | P49748 | ACADVL | -1.26 |
|  |  |  | P49790 | NUP153 | -0.85 |
|  |  |  | P50995 | ANXA11 | -1.19 |
|  |  |  | P52565 | ARHGDIA | 1.57 |
|  |  |  | P55010 | EIF5 | 2.40 |
|  |  |  | P55060-3 | CSE1L | 0.83 |
|  |  |  | P55809 | OXCT1 | -1.07 |
|  |  |  | P55884 | EIF3B | 0.75 |
|  |  |  | P60174-1 | TPI1 | 0.78 |
|  |  |  | P61221 | ABCE1 | 1.11 |
|  |  |  | P61586 | RHOA | 1.46 |
|  |  |  | P62888 | RPL30 | 1.55 |
|  |  |  | Q5JPE7-2 | NOMO3;NOMO2 | 1.00 |
|  |  |  | Q12849 | GRSF1 | -1.10 |
|  |  |  | Q13242 | SRSF9 | 0.76 |
|  |  |  | Q13263 | TRIM28 | -1.01 |
|  |  |  | Q15046 | KARS | 1.06 |
|  |  |  | Q16222-3 | UAP1 | 0.22 |
|  |  |  | Q8N684-2 | CPSF7 | -1.60 |
|  |  |  | Q8TEM1 | NUP210 | -0.72 |
|  |  |  | Q8WXF1-2 | PSPC1 | 0.89 |
|  |  |  | Q92598-2 | HSPH1 | 1.59 |
|  |  |  | Q969V3-2 | NCLN | 0.66 |
|  |  |  | Q96KP4 | CNDP2 | 1.04 |
|  |  |  | Q99623 | PHB2 | -1.70 |
|  |  |  | Q9BPW8 | NIPSNAP1 | -1.77 |
|  |  |  | Q9C0B1 | FTO | 0.80 |
|  |  |  | Q9NYU2-2 | UGGT1 | -1.15 |
|  |  |  | Q9P2J5 | LARS | 0.49 |
|  |  |  | Q9UBQ5 | EIF3K | 2.20 |
|  |  |  | Q9UM00-2 | TMCO1 | -1.15 |
|  |  |  | Q9Y266 | NUDC | 0.92 |
|  |  |  | Q9Y2X3 | NOP58 | 1.49 |
|  |  |  | Q9Y3F4 | STRAP | 1.60 |

**Table S.2. Common Significantly Changing Proteins Between Androgen Sensitive and Androgen Independent Cell Lines**

| **Common ANOVA Significant** | | | **24 Hours** | | | | | | **48 Hours** | | | | | |
| --- | --- | --- | --- | --- | --- | --- | --- | --- | --- | --- | --- | --- | --- | --- |
| **Protein ID** | **Gene name** | **KEGG name** | **Av Abl 24 C** | **Av Abl 24 LG** | **Av Hof 24 C** | **Av Hof 24 LG** | **Av LNCaP 24 C** | **Av LNCaP 24 LG** | **AV Abl 48 C** | **AV Abl 48 LG** | **AV Hof 48 C** | **AV Hof 48 LG** | **AV LNCaP 48 C** | **AV LNCaP 48 LG** |
| O00159-3 | MYO1C |  | -0.61 | -0.69 | -0.51 | -0.29 | 1.81 | 1.07 | -0.44 | -0.57 | -0.48 | -0.08 | 1.23 | 1.66 |
| O00264 | PGRMC1 |  | -0.67 | -0.89 | -0.85 | -0.76 | 1.60 | 0.78 | -0.79 | -0.87 | -1.21 | 0.28 | 0.96 | 1.47 |
| O15394 | NCAM2 |  | -1.62 | -1.31 | 1.23 | 1.08 | -0.12 | 0.20 | -0.44 | -0.94 | -0.39 | -1.07 | 1.49 | 0.62 |
| O43399 | TPD52L2 |  | -0.71 | -0.62 | -0.59 | -0.41 | 0.74 | 1.98 | -0.39 | -0.57 | -1.09 | -0.59 | 0.83 | 1.87 |
| O60313 | OPA1 |  | -1.09 | -0.24 | -0.94 | -0.57 | 1.18 | 1.31 | -0.53 | -0.68 | -0.21 | -0.76 | 1.50 | 1.18 |
| O75152 | ZC3H11A | Alzheimer's disease | -0.82 | -1.22 | -0.19 | -0.15 | 1.25 | 1.10 | -0.80 | -0.53 | -0.70 | -0.86 | 1.37 | 1.46 |
| O95168-2 | NDUFB4 | Adipocytokine signaling pathway | -0.76 | -0.68 | -0.80 | -0.42 | 1.24 | 1.32 | -1.02 | -0.77 | -0.70 | -0.53 | 1.66 | 1.50 |
| O95573 | ACSL3 | Cysteine and methionine metabolism | -1.31 | -1.04 | -0.51 | -0.56 | 1.26 | 1.39 | -0.45 | -1.45 | 0.06 | -0.56 | 1.52 | -0.07 |
| P00338 | LDHA | Alanine, aspartate and glutamate metabolism | -0.53 | -1.07 | -0.68 | -0.77 | 1.77 | 0.90 | -0.86 | -0.66 | -0.58 | -0.61 | 1.21 | 1.82 |
| P00367 | GLUD1 | Endocytosis | -0.93 | -1.32 | -0.48 | -0.54 | 1.43 | 1.02 | -0.99 | -0.10 | -1.03 | 0.28 | 0.09 | 1.99 |
| P02786 | TFRC | Mineral absorption | -1.61 | -0.15 | -0.46 | 0.11 | 0.22 | 1.67 | 1.00 | -0.33 | 0.73 | -0.82 | 0.70 | -1.99 |
| P02787 | TF |  | 1.00 | -1.01 | 1.12 | -0.96 | 0.86 | -1.32 | -1.59 | -0.90 | -0.12 | 0.44 | 0.73 | 0.85 |
| P05783 | KRT18 | Cysteine and methionine metabolism | -1.68 | -1.15 | 0.35 | 0.42 | 1.02 | 0.52 | -0.32 | -0.61 | -1.16 | -1.45 | 1.34 | 1.17 |
| P07195 | LDHB | Alzheimer's disease | -0.43 | -0.60 | -1.19 | -1.26 | 1.39 | 1.35 | 0.37 | 0.16 | -1.23 | -1.14 | 0.60 | 1.25 |
| P08473 | MME | Base excision repair | -0.10 | 0.58 | -1.50 | -1.22 | 0.51 | 1.32 | -1.19 | -1.35 | -0.11 | -0.02 | 1.47 | 1.15 |
| P09874 | PARP1 | Citrate cycle (TCA cycle) | -1.03 | -1.42 | 0.14 | -0.53 | 1.33 | 0.83 | -0.83 | -0.40 | -0.94 | -0.31 | 0.56 | 2.03 |
| P10515 | DLAT | Drug metabolism - other enzymes | -1.11 | 0.02 | -0.32 | -0.80 | 1.17 | 1.36 | -0.33 | -1.50 | -0.22 | -0.66 | 1.44 | 0.98 |
| P12268 | IMPDH2 | Non-homologous end-joining | -0.72 | -0.67 | -0.66 | -0.78 | 1.34 | 1.62 | -0.61 | -1.29 | -0.35 | -0.66 | 1.44 | 0.61 |
| P12956 | XRCC6 | Non-homologous end-joining | -0.32 | -1.03 | -0.89 | -0.90 | 1.43 | 0.99 | -0.91 | -0.68 | -0.56 | -0.95 | 1.61 | 1.12 |
| P13010 | XRCC5 | Focal adhesion | -0.49 | -1.01 | -0.35 | -0.71 | 1.34 | 1.61 | -0.31 | -0.44 | -0.64 | -1.12 | 1.34 | 1.14 |
| P21333-2 | FLNA |  | -0.23 | -0.45 | -0.77 | -1.05 | 1.13 | 1.50 | -0.94 | -0.28 | -0.98 | -0.50 | 1.63 | 1.09 |
| P22570 | FDXR | Alzheimer's disease | -0.83 | -1.12 | -0.68 | -0.65 | 1.35 | 1.41 | 0.02 | -0.60 | -1.01 | -0.25 | 1.64 | 1.41 |
| P22695 | UQCRC2 | Porphyrin and chlorophyll metabolism | -1.11 | -0.43 | -0.90 | -0.66 | 1.13 | 1.12 | -0.40 | -0.09 | -1.37 | -1.02 | 1.35 | 1.38 |
| P22830 | FECH | Alzheimer's disease | -0.04 | 0.05 | -1.29 | -1.31 | 1.16 | 1.29 | -0.87 | -1.18 | 0.00 | -0.43 | 1.28 | 1.01 |
| P28331 | NDUFS1 | Biosynthesis of ansamycins | -0.77 | -1.32 | -0.31 | -0.50 | 1.27 | 1.15 | -0.58 | -0.85 | -0.79 | -0.90 | 1.24 | 1.55 |
| P29401 | TKT | Methane metabolism | -0.45 | -0.57 | -0.69 | -1.12 | 1.35 | 1.59 | 0.10 | -1.13 | -0.24 | -1.16 | 1.29 | 0.11 |
| P30041 | PRDX6 | Arginine and proline metabolism | 0.34 | -0.09 | -1.30 | -1.08 | 1.08 | 1.37 | -0.67 | -1.13 | -0.28 | -0.98 | 1.36 | 1.52 |
| P30837 | ALDH1B1 | Alzheimer's disease | -1.17 | -1.00 | -0.34 | -0.23 | 1.45 | 1.35 | -0.24 | 0.02 | -0.79 | -1.26 | 1.62 | 1.25 |
| P31040 | SDHA | Alzheimer's disease | -0.54 | -0.92 | -0.44 | -1.21 | 1.45 | 0.68 | -0.68 | -0.96 | -0.49 | -0.49 | 1.46 | 1.39 |
| P31930 | UQCRC1 | Arginine and proline metabolism | -1.24 | -0.61 | -0.51 | -0.55 | 1.57 | 1.17 | -0.47 | -0.41 | -0.89 | -0.98 | 1.35 | 1.05 |
| P32322 | PYCR1 |  | -1.12 | -0.80 | -0.37 | -0.83 | 1.48 | 0.93 | -0.05 | -0.70 | -0.84 | -0.76 | 0.77 | 1.58 |
| P35232 | PHB | Tight junction | -1.18 | -0.06 | -0.68 | -1.03 | 1.29 | 1.21 | -1.06 | -0.79 | -0.27 | -0.89 | 1.70 | 1.16 |
| P35580 | MYH10 | Citrate cycle (TCA cycle) | -0.81 | -0.68 | -0.55 | -0.78 | 1.25 | 1.89 | -0.14 | 0.08 | -1.65 | -0.67 | 0.55 | 1.67 |
| P36957 | DLST |  | -0.68 | -0.01 | -1.38 | -0.65 | 1.00 | 1.60 | -0.67 | -0.79 | -1.10 | -0.37 | 1.17 | 1.81 |
| P42704 | LRPPRC | Nicotinate and nicotinamide metabolism | -1.23 | -0.67 | -0.65 | -0.66 | 1.23 | 1.45 | 0.18 | -0.33 | -0.81 | -1.62 | 1.28 | 1.02 |
| P43490 | NAMPT | Glutathione metabolism | 0.29 | 0.63 | -1.06 | -1.45 | 0.76 | 1.37 | -0.42 | -1.45 | -0.72 | -0.50 | 0.92 | 0.33 |
| P48637 | GSS |  | -0.16 | -0.64 | -1.06 | -0.88 | 1.15 | 1.22 | -0.45 | -1.56 | -0.29 | -0.67 | 1.38 | 1.09 |
| P49321 | NASP | Fatty acid metabolism | -0.32 | -0.84 | -0.70 | -0.96 | 1.36 | 1.53 | -0.46 | 0.26 | -1.73 | -0.47 | 0.63 | 1.31 |
| P49748 | ACADVL | Alzheimer's disease | -0.14 | 0.16 | -1.39 | -1.14 | 1.02 | 1.33 | -0.47 | 0.04 | -0.92 | -0.45 | 1.06 | 1.65 |
| P49821-2 | NDUFV1 | Drug metabolism - other enzymes | -0.77 | -1.01 | -0.63 | -0.28 | 1.04 | 1.58 | -0.60 | -0.84 | -0.30 | -0.78 | 1.79 | 0.73 |
| P49915 | GMPS | Alzheimer's disease | -0.27 | -0.50 | -1.06 | -0.90 | 1.44 | 1.43 | -0.63 | -1.25 | -0.79 | -0.13 | 1.28 | 1.26 |
| P51970 | NDUFA8 |  | -0.85 | -0.32 | -0.94 | -0.40 | 1.14 | 1.45 | -0.35 | -1.42 | 0.46 | -0.38 | 1.65 | 0.69 |
| P55060-3 | CSE1L | Butanoate metabolism | -0.33 | -0.69 | -0.66 | -0.78 | 1.21 | 1.70 | -0.65 | 0.22 | -1.50 | -0.43 | 1.04 | 1.64 |
| P55809 | OXCT1 | Antigen processing and presentation | -0.81 | -0.34 | -1.02 | -1.04 | 1.16 | 1.35 | 0.30 | -1.51 | -0.53 | -0.10 | 1.34 | 0.78 |
| P61289 | PSME3 |  | -0.27 | -0.58 | -1.02 | -0.67 | 1.54 | 1.25 | -1.25 | -0.57 | -0.72 | -0.32 | 0.35 | 1.41 |
| P61604 | HSPE1 |  | -1.19 | -1.11 | 0.06 | -0.83 | 1.07 | 0.70 | -0.78 | -0.99 | -0.50 | -0.18 | 1.38 | 1.50 |
| Q12931 | TRAP1 |  | -0.90 | -1.31 | -0.08 | -0.77 | 1.33 | 1.11 | -0.98 | -0.72 | -0.81 | 0.19 | 1.62 | 1.14 |
| Q13263 | TRIM28 | Arrhythmogenic right ventricular cardiomyopathy (ARVC) | -0.77 | -0.92 | -0.76 | -0.44 | 1.26 | 1.69 | -0.87 | -1.34 | 0.03 | -0.52 | 1.54 | 1.27 |
| Q14126 | DSG2 |  | -1.18 | -1.61 | -0.21 | 0.01 | 1.15 | 1.31 | -0.44 | -0.04 | 0.03 | -0.63 | 1.51 | 1.10 |
| Q15691 | MAPRE1 | Amino sugar and nucleotide sugar metabolism | 0.66 | -0.51 | -0.50 | -1.43 | 1.22 | 0.86 | -0.34 | -0.96 | -1.00 | -1.22 | 1.44 | 1.20 |
| Q16222-3 | UAP1 |  | -0.38 | -0.51 | -1.14 | -1.24 | 1.43 | 1.39 | -0.08 | -0.20 | -0.95 | -1.15 | 0.65 | 1.94 |
| Q16891-2 | IMMT |  | -1.02 | -0.72 | -0.15 | -0.87 | 1.31 | 1.33 | -0.90 | -0.67 | -0.85 | -0.55 | 1.65 | 1.24 |
| Q5VWZ2 | LYPLAL1 | Pentose and glucuronate interconversions | -1.10 | -0.62 | -0.49 | -0.72 | 1.56 | 1.26 | -0.85 | 0.39 | -1.30 | -0.73 | 0.77 | 1.63 |
| Q7Z4W1 | DCXR |  | -0.78 | -0.30 | -1.05 | -0.64 | 1.02 | 1.45 | -0.09 | -0.95 | -0.92 | -0.36 | 1.70 | 1.02 |
| Q8NCW5-2 | APOA1BP |  | -0.49 | -0.18 | -1.27 | -0.79 | 1.25 | 1.30 | -0.71 | -0.66 | -0.96 | -0.64 | 1.25 | 1.31 |
| Q8NFV4 | ABHD11 | Protein processing in endoplasmic reticulum | -0.76 | -0.95 | -0.61 | -0.99 | 1.50 | 1.25 | 0.64 | -0.92 | 0.09 | -1.50 | 1.35 | -0.32 |
| Q92598-2 | HSPH1 |  | 0.48 | -0.18 | -1.11 | -1.23 | 1.26 | 1.23 | -0.61 | -0.49 | -1.23 | -0.33 | 1.07 | 1.49 |
| Q96ER9 | CCDC51 |  | -0.57 | -0.54 | -0.57 | -1.25 | 1.40 | 1.14 | -0.49 | -1.18 | -0.46 | -0.72 | 1.22 | 1.45 |
| Q96HS1 | PGAM5 |  | -0.61 | -0.74 | -0.80 | -0.96 | 1.35 | 1.28 | -0.06 | -0.01 | -1.80 | -0.10 | 0.87 | 1.13 |
| Q99623 | PHB2 |  | -0.96 | -0.25 | -0.79 | -1.02 | 0.89 | 1.36 | -0.61 | -0.87 | 0.01 | -0.82 | 1.30 | 1.23 |
| Q9BS26 | ERP44 | Ribosome biogenesis in eukaryotes | -0.38 | -0.22 | 0.11 | -1.04 | 0.84 | 1.66 | -1.34 | -1.08 | -0.30 | -0.25 | 0.84 | 1.33 |
| Q9BVP2-2 | GNL3 |  | -1.36 | -0.73 | 0.21 | -0.70 | 1.01 | 1.40 | -0.84 | -0.93 | -0.27 | -0.50 | 0.91 | 1.52 |
| Q9GZR7 | DDX24 | RNA degradation | -0.87 | -0.65 | 0.93 | -0.99 | 1.51 | 0.68 | -0.71 | -0.86 | -1.02 | -0.96 | 1.47 | 1.31 |
| Q9H2U1 | DHX36 |  | -0.54 | -1.11 | -0.94 | -0.80 | 1.30 | 1.40 | -0.77 | 0.05 | -0.77 | -1.14 | 1.05 | 1.41 |
| Q9HAV7 | GRPEL1 |  | -0.78 | -0.98 | -0.70 | -0.69 | 1.43 | 1.19 | -0.85 | -0.70 | -0.84 | -0.90 | 1.29 | 1.70 |
| Q9UJZ1 | STOML2 |  | -0.80 | -0.80 | -0.60 | -0.99 | 1.33 | 1.57 | 0.02 | -0.58 | 0.11 | -1.67 | 1.19 | 0.49 |
| Q9UK76 | HN1 |  | -0.06 | -0.69 | -0.41 | -1.42 | 1.01 | 1.39 | -0.30 | -0.10 | -1.25 | -1.02 | 0.68 | 1.78 |
| Q9UPQ0-10 | LIMCH1 |  | -0.34 | -0.32 | -0.90 | -0.94 | 1.07 | 1.54 | -0.51 | -0.46 | -0.99 | -0.69 | 0.89 | 1.37 |
| Q9Y2S7 | POLDIP2 |  | -0.80 | 0.15 | -1.00 | -1.11 | 0.95 | 1.32 | -1.15 | 0.05 | -0.23 | -0.15 | 0.84 | 1.67 |
| Q9Y305-3 | ACOT9 | Protein export | -1.18 | -0.95 | 0.09 | -0.38 | 0.77 | 1.39 | -1.02 | -1.21 | 0.08 | -0.62 | 1.05 | 1.20 |
| Q9Y5M8 | SRPRB | Ether lipid metabolism | -1.67 | -0.81 | 0.71 | -0.01 | 1.00 | 0.44 | 0.65 | 0.32 | 1.00 | 0.80 | -1.56 | -1.38 |
| O00116 | AGPS |  | 0.56 | 0.53 | 1.01 | 0.94 | -1.24 | -1.55 | 0.55 | 0.17 | 0.95 | 0.49 | -1.06 | -1.89 |
| O00299 | CLIC1 |  | 0.73 | 0.81 | 0.74 | 0.76 | -1.60 | -1.43 | 0.78 | 0.91 | 0.49 | 0.88 | -1.50 | -1.35 |
| O15020-2 | SPTBN2 | Cell adhesion molecules (CAMs) | 0.51 | 0.72 | 0.77 | 0.86 | -1.77 | -1.19 | -1.14 | -1.43 | 0.96 | 1.30 | -0.39 | 0.56 |
| O43175 | PHGDH | Glycine, serine and threonine metabolism | 0.77 | 0.74 | 0.70 | 0.52 | -1.71 | -1.58 | 0.72 | 0.58 | 0.79 | 0.65 | -1.50 | -1.71 |
| O43747 | AP1G1 | Lysosome | 0.72 | 0.99 | 0.44 | 0.60 | -1.58 | -1.03 | 0.70 | 1.09 | 0.51 | 0.51 | -1.29 | -1.38 |
| O75369-2 | FLNB | Focal adhesion | 0.89 | 0.64 | 0.80 | 0.39 | -1.62 | -1.57 | 0.86 | 0.57 | 0.91 | 0.41 | -1.76 | -1.48 |
| O75795 | UGT2B17 | Ascorbate and aldarate metabolism | 1.07 | 0.80 | 0.53 | 0.03 | -1.44 | -1.60 | 1.02 | 0.95 | 0.32 | 0.20 | -1.81 | -0.99 |
| P00441 | SOD1 | Amyotrophic lateral sclerosis (ALS) | 1.39 | 0.68 | 0.31 | 0.09 | -1.45 | -0.82 | 0.93 | 0.08 | 0.45 | 0.54 | -1.04 | -1.76 |
| P04075 | ALDOA | Carbon fixation in photosynthetic organisms | 0.97 | 0.73 | 0.65 | 0.06 | -1.14 | -1.48 | 0.62 | 0.72 | 0.91 | 0.42 | -1.27 | -1.76 |
| P04792 | HSPB1 | Amoebiasis | 1.17 | 0.66 | 0.09 | 0.41 | -1.23 | -1.63 | 0.87 | -0.27 | 0.95 | -0.14 | -0.59 | -1.81 |
| P07099 | EPHX1 | Bile secretion | 0.97 | 1.03 | 0.09 | 0.39 | -1.56 | -1.49 | 1.30 | 1.00 | -0.04 | 0.41 | -1.44 | -1.48 |
| P07237 | P4HB | Protein processing in endoplasmic reticulum | 0.53 | 0.56 | 0.56 | 0.31 | -1.19 | -1.80 | 1.22 | 0.51 | 0.01 | 0.52 | -1.59 | -1.25 |
| P07339 | CTSD | ko05152 | 0.32 | 0.84 | 0.97 | 0.64 | -1.60 | -1.46 | 0.74 | 0.46 | 0.39 | 0.83 | -1.52 | -1.20 |
| P08133 | ANXA6 |  | 0.54 | 0.75 | 0.69 | 0.76 | -1.58 | -1.64 | 0.70 | 0.40 | 0.58 | 0.49 | -1.00 | -1.90 |
| P08758 | ANXA5 |  | 1.03 | 1.55 | -0.41 | 0.17 | -1.02 | -1.15 | 1.46 | 0.48 | -1.12 | -0.19 | -0.50 | -1.29 |
| P11413 | G6PD | Glutathione metabolism | 0.53 | 0.54 | 0.32 | 1.07 | -1.34 | -1.47 | 0.45 | 0.13 | 0.76 | 0.84 | -1.01 | -1.79 |
| P13667 | PDIA4 | Protein processing in endoplasmic reticulum | 0.27 | 0.55 | 1.05 | 0.74 | -1.33 | -1.78 | 0.72 | 0.50 | 1.10 | 0.42 | -1.69 | -1.33 |
| P14625 | HSP90B1 | NOD-like receptor signaling pathway | 0.03 | 0.06 | 1.04 | 0.87 | -0.97 | -1.69 | 0.49 | -0.82 | 1.23 | 0.81 | -1.08 | -1.09 |
| P16403 | HIST1H1C |  | 0.33 | 0.48 | 0.82 | 0.20 | -1.05 | -1.83 | 0.66 | 1.16 | 0.08 | 0.54 | -1.49 | -1.40 |
| P20073-2 | ANXA7 |  | 0.19 | 1.07 | 0.41 | 0.87 | -1.52 | -1.18 | 0.20 | 0.78 | 0.26 | 0.92 | -1.54 | -1.40 |
| P20700 | LMNB1 |  | 0.72 | 0.49 | 0.63 | 0.32 | -1.14 | -1.83 | 0.78 | 0.69 | 0.28 | 0.68 | -1.79 | -1.01 |
| P21291 | CSRP1 |  | 0.56 | 0.49 | 0.82 | 0.72 | -1.41 | -1.56 | 0.73 | 0.10 | 0.92 | 0.70 | -0.84 | -2.01 |
| P23246 | SFPQ |  | 0.36 | -0.86 | 0.81 | 0.84 | -1.03 | -1.09 | 0.70 | 0.36 | 0.47 | 0.98 | -1.26 | -1.24 |
| P26639 | TARS | Aminoacyl-tRNA biosynthesis | 1.28 | 0.38 | 0.79 | 0.25 | -1.30 | -1.51 | 1.32 | 0.00 | 1.21 | 0.11 | -1.04 | -1.57 |
| P30101 | PDIA3 | Antigen processing and presentation | 0.51 | -0.14 | 1.09 | 0.75 | -1.25 | -1.69 | 0.82 | 0.44 | 0.35 | 0.47 | -1.47 | -1.33 |
| P30533 | LRPAP1 |  | 0.37 | 0.49 | 0.92 | 0.91 | -1.47 | -1.54 | 0.59 | 0.50 | 0.75 | 0.88 | -1.39 | -1.78 |
| P33121 | ACSL1 | Adipocytokine signaling pathway | 0.35 | 0.59 | 0.63 | 0.84 | -1.32 | -1.82 | 0.48 | 0.77 | 0.64 | 0.89 | -1.32 | -1.75 |
| P35659 | DEK |  | 0.65 | 0.76 | 0.59 | 0.72 | -1.22 | -1.78 | 1.08 | -0.74 | 1.19 | 0.60 | -1.02 | -0.90 |
| P41250 | GARS | Aminoacyl-tRNA biosynthesis | 0.07 | -0.82 | 1.41 | 1.30 | -0.84 | -1.07 | -0.29 | -0.07 | 1.35 | 0.98 | -1.42 | -1.01 |
| P46940 | IQGAP1 | Regulation of actin cytoskeleton | 0.31 | 0.73 | 1.19 | 0.67 | -1.81 | -0.94 | 0.35 | 0.25 | 0.77 | 1.18 | -1.78 | -1.08 |
| P46977 | STT3A | N-Glycan biosynthesis | 0.45 | 0.13 | 0.89 | 1.00 | -1.30 | -1.28 | 0.76 | -0.22 | 1.16 | 0.71 | -1.18 | -1.17 |
| P49189 | ALDH9A1 | Arginine and proline metabolism | 1.15 | 0.83 | 0.14 | 0.24 | -1.30 | -1.18 | 0.47 | 1.21 | -0.21 | 0.28 | -1.84 | -0.90 |
| P49257 | LMAN1 | Protein processing in endoplasmic reticulum | 0.16 | -0.55 | 0.69 | 1.26 | -1.11 | -1.13 | 0.64 | -0.57 | 1.35 | 0.81 | -0.84 | -1.22 |
| P49588 | AARS | Aminoacyl-tRNA biosynthesis | 0.66 | 0.45 | 1.00 | 0.63 | -1.44 | -1.63 | 0.59 | 0.23 | 1.19 | 0.72 | -1.23 | -1.76 |
| P52209 | PGD | Glutathione metabolism | 0.84 | 0.54 | 0.75 | 0.82 | -1.64 | -1.24 | 0.50 | 0.01 | 0.88 | 0.85 | -0.33 | -1.80 |
| P63010 | AP2B1 | Endocrine and other factor-regulated calcium reabsorption | 0.39 | 1.17 | 0.72 | 0.16 | -1.28 | -1.34 | -0.22 | 1.36 | 0.41 | 0.93 | -1.54 | -0.47 |
| Q5JPE7-2 | NOMO3 |  | 0.26 | 0.85 | 1.14 | 0.52 | -1.57 | -1.23 | 0.33 | 0.70 | 1.16 | 0.16 | -2.04 | -0.60 |
| P78527 | PRKDC | Cell cycle | 0.32 | 0.44 | 0.95 | 1.12 | -1.60 | -1.26 | 0.31 | -0.07 | 1.07 | 1.50 | -0.95 | -1.01 |
| P78540 | ARG2 | Amoebiasis | -0.10 | -0.27 | 1.16 | 0.92 | -1.30 | -1.17 | -0.43 | 0.03 | 1.06 | 1.10 | -1.17 | -1.15 |
| P80303-2 | NUCB2 |  | 0.61 | 0.94 | 0.58 | 0.62 | -1.52 | -1.70 | 0.78 | 0.82 | 0.60 | 0.68 | -1.46 | -1.70 |
| P98179 | RBM3 |  | 0.73 | 0.12 | 0.22 | 0.56 | -1.16 | -1.74 | 0.76 | 1.16 | 0.24 | 0.18 | -0.88 | -1.64 |
| Q00341 | HDLBP |  | 0.74 | 0.58 | 1.03 | 0.60 | -1.63 | -1.44 | 1.06 | 0.81 | 0.82 | 0.01 | -1.77 | -1.14 |
| Q13228 | SELENBP1 |  | 1.02 | 0.98 | 0.39 | 0.52 | -1.60 | -1.47 | 0.80 | 0.49 | 0.54 | 0.49 | -1.20 | -1.80 |
| Q13425 | SNTB2 |  | 0.52 | 0.68 | 1.07 | 0.61 | -1.53 | -1.16 | 0.30 | 1.07 | 0.53 | 0.87 | -1.25 | -0.94 |
| Q13501 | SQSTM1 | Osteoclast differentiation | 0.66 | 0.84 | 0.52 | 1.01 | -1.53 | -1.51 | 0.39 | 0.62 | 0.52 | 0.67 | -1.76 | -1.36 |
| Q14739 | LBR |  | 0.82 | 0.75 | 0.72 | 0.62 | -1.61 | -1.43 | 0.91 | 0.83 | 0.40 | 0.73 | -1.85 | -1.06 |
| Q14938-5 | NFIX |  | 0.73 | 0.64 | 0.84 | 0.55 | -1.51 | -1.71 | 0.74 | 0.64 | 0.80 | 0.44 | -1.75 | -0.79 |
| Q15046 | KARS | Aminoacyl-tRNA biosynthesis | 0.97 | 0.32 | 0.69 | 0.23 | -1.32 | -1.44 | 0.46 | 0.04 | 1.12 | 0.06 | -1.01 | -1.61 |
| Q6DD88 | ATL3 |  | 0.83 | 0.82 | 0.56 | 0.51 | -1.60 | -1.66 | 0.78 | 0.72 | 0.70 | 0.70 | -1.88 | -1.14 |
| Q6NVY1 | HIBCH | beta-Alanine metabolism | 0.61 | 0.53 | 0.77 | 0.80 | -1.80 | -1.39 | 0.50 | 0.45 | 0.57 | 0.92 | -1.54 | -1.48 |
| Q6UB35 | MTHFD1L | One carbon pool by folate | 0.21 | 0.14 | 1.36 | 0.81 | -1.25 | -1.62 | 0.17 | 0.46 | 1.11 | 0.48 | -1.66 | -0.93 |
| Q7KZF4 | SND1 |  | 0.16 | 0.12 | 1.18 | 0.94 | -1.42 | -1.47 | 0.34 | 0.41 | 1.12 | 1.07 | -1.68 | -0.86 |
| Q8NBS9 | TXNDC5 | Protein processing in endoplasmic reticulum | 0.48 | 0.47 | 1.02 | 0.68 | -1.58 | -1.63 | 0.74 | 0.49 | 0.77 | 0.93 | -1.67 | -1.35 |
| Q8TEM1 | NUP210 | RNA transport | 0.29 | 0.85 | 0.94 | 0.68 | -1.71 | -1.28 | 0.35 | 1.02 | 0.49 | 1.21 | -1.77 | -0.87 |
| Q8WUY1 | THEM6 |  | -0.55 | -0.53 | 1.01 | 1.63 | -0.92 | -0.90 | -0.55 | 0.32 | 1.30 | 1.06 | -1.23 | -0.64 |
| Q9H2U2 | PPA2 | Oxidative phosphorylation | 0.52 | 0.67 | 0.78 | 0.73 | -1.68 | -1.35 | 0.53 | 0.77 | 0.77 | 0.90 | -1.80 | -1.19 |
| Q9NUJ1 | ABHD10 |  | 0.78 | 1.08 | 0.00 | 0.29 | -1.40 | -1.31 | 0.77 | 0.72 | 0.67 | 0.37 | -1.69 | -0.78 |
| Q9P2E9-2 | RRBP1 | Protein processing in endoplasmic reticulum | 0.75 | 0.48 | 1.01 | 0.60 | -1.63 | -1.40 | 0.60 | 1.27 | 0.53 | 0.56 | -1.77 | -1.23 |
| Q9P2J5 | LARS | Aminoacyl-tRNA biosynthesis | 0.55 | 0.21 | 1.07 | 1.08 | -1.36 | -1.31 | 0.36 | -0.56 | 1.31 | 0.82 | -0.75 | -1.59 |
| Q9UM54-6 |  |  | 0.98 | 0.78 | 0.61 | 0.58 | -1.67 | -1.42 | 0.88 | 0.45 | 0.52 | 0.61 | -1.53 | -1.70 |
| Q9Y4L1 | HYOU1 | Protein processing in endoplasmic reticulum | 0.12 | -0.03 | 1.40 | 0.91 | -1.38 | -1.46 | 0.46 | 0.02 | 1.12 | 0.99 | -1.63 | -1.33 |
| Q9Y5P6 | GMPPB | Amino sugar and nucleotide sugar metabolism | 0.69 | 0.76 | 0.76 | 0.69 | -1.70 | -0.96 | 0.67 | -0.14 | 1.40 | 0.25 | -1.12 | -1.27 |

**Table S.3. Transition List for Hx Panel**

| **Compound Group** | **Compound Name** | **ISTD?** | **Precursor Ion** | **MS1 Res** | **Product Ion** | **MS2 Res** | **Dwell** | **Fragmentor** | **Collision Energy** | **Cell Accelerator Voltage** | **Polarity** |
| --- | --- | --- | --- | --- | --- | --- | --- | --- | --- | --- | --- |
| sp\|P38606\|VATA_HUMAN | LAEMPADSGYPAYLGAR.light | FALSE | 891.43 | Unit | 1169.56 | Unit | 10 | 130 | 28.6 | 4 | Positive |
| sp\|P38606\|VATA_HUMAN | LAEMPADSGYPAYLGAR.light | FALSE | 891.43 | Unit | 1054.53 | Unit | 10 | 130 | 28.6 | 4 | Positive |
| sp\|P38606\|VATA_HUMAN | LAEMPADSGYPAYLGAR.light | FALSE | 891.43 | Unit | 967.50 | Unit | 10 | 130 | 28.6 | 4 | Positive |
| sp\|P38606\|VATA_HUMAN | LAEMPADSGYPAYLGAR.light | FALSE | 891.43 | Unit | 747.41 | Unit | 10 | 130 | 28.6 | 4 | Positive |
| sp\|P38606\|VATA_HUMAN | EHMGDILYK.light | FALSE | 553.27 | Unit | 976.49 | Unit | 10 | 130 | 18.2 | 4 | Positive |
| sp\|P38606\|VATA_HUMAN | EHMGDILYK.light | FALSE | 553.27 | Unit | 839.43 | Unit | 10 | 130 | 18.2 | 4 | Positive |
| sp\|P38606\|VATA_HUMAN | EHMGDILYK.light | FALSE | 553.27 | Unit | 310.18 | Unit | 10 | 130 | 18.2 | 4 | Positive |
| sp\|P38606\|VATA_HUMAN | EHMGDILYK.light | FALSE | 553.27 | Unit | 147.11 | Unit | 10 | 130 | 18.2 | 4 | Positive |
| sp\|P04040\|CATA_HUMAN | ADVLTTGAGNPVGDK.light | FALSE | 707.86 | Unit | 1016.50 | Unit | 10 | 130 | 22.9 | 4 | Positive |
| sp\|P04040\|CATA_HUMAN | ADVLTTGAGNPVGDK.light | FALSE | 707.86 | Unit | 915.45 | Unit | 10 | 130 | 22.9 | 4 | Positive |
| sp\|P04040\|CATA_HUMAN | ADVLTTGAGNPVGDK.light | FALSE | 707.86 | Unit | 814.41 | Unit | 10 | 130 | 22.9 | 4 | Positive |
| sp\|P04040\|CATA_HUMAN | ADVLTTGAGNPVGDK.light | FALSE | 707.86 | Unit | 515.28 | Unit | 10 | 130 | 22.9 | 4 | Positive |
| sp\|P04040\|CATA_HUMAN | LNVITVGPR.light | FALSE | 484.80 | Unit | 855.50 | Unit | 10 | 130 | 16 | 4 | Positive |
| sp\|P04040\|CATA_HUMAN | LNVITVGPR.light | FALSE | 484.80 | Unit | 741.46 | Unit | 10 | 130 | 16 | 4 | Positive |
| sp\|P04040\|CATA_HUMAN | LNVITVGPR.light | FALSE | 484.80 | Unit | 642.39 | Unit | 10 | 130 | 16 | 4 | Positive |
| sp\|P04040\|CATA_HUMAN | LNVITVGPR.light | FALSE | 484.80 | Unit | 529.31 | Unit | 10 | 130 | 16 | 4 | Positive |
| sp\|P12830\|CADH1_HUMAN | VGTDGVITVK.light | FALSE | 494.79 | Unit | 889.50 | Unit | 10 | 130 | 16.3 | 4 | Positive |
| sp\|P12830\|CADH1_HUMAN | VGTDGVITVK.light | FALSE | 494.79 | Unit | 616.40 | Unit | 10 | 130 | 16.3 | 4 | Positive |
| sp\|P12830\|CADH1_HUMAN | VGTDGVITVK.light | FALSE | 494.79 | Unit | 347.23 | Unit | 10 | 130 | 16.3 | 4 | Positive |
| sp\|P12830\|CADH1_HUMAN | VGTDGVITVK.light | FALSE | 494.79 | Unit | 147.11 | Unit | 10 | 130 | 16.3 | 4 | Positive |
| sp\|P12830\|CADH1_HUMAN | DTANWLEINPDTGAISTR.light | FALSE | 987.48 | Unit | 1144.60 | Unit | 10 | 130 | 31.6 | 4 | Positive |
| sp\|P12830\|CADH1_HUMAN | DTANWLEINPDTGAISTR.light | FALSE | 987.48 | Unit | 1031.51 | Unit | 10 | 130 | 31.6 | 4 | Positive |
| sp\|P12830\|CADH1_HUMAN | DTANWLEINPDTGAISTR.light | FALSE | 987.48 | Unit | 917.47 | Unit | 10 | 130 | 31.6 | 4 | Positive |
| sp\|P12830\|CADH1_HUMAN | DTANWLEINPDTGAISTR.light | FALSE | 987.48 | Unit | 705.39 | Unit | 10 | 130 | 31.6 | 4 | Positive |
| sp\|P33316\|DUT_HUMAN | LSEHATAPTR.light | FALSE | 541.78 | Unit | 969.47 | Unit | 10 | 130 | 17.8 | 4 | Positive |
| sp\|P33316\|DUT_HUMAN | LSEHATAPTR.light | FALSE | 541.78 | Unit | 753.40 | Unit | 10 | 130 | 17.8 | 4 | Positive |
| sp\|P33316\|DUT_HUMAN | LSEHATAPTR.light | FALSE | 541.78 | Unit | 616.34 | Unit | 10 | 130 | 17.8 | 4 | Positive |
| sp\|P33316\|DUT_HUMAN | LSEHATAPTR.light | FALSE | 541.78 | Unit | 545.30 | Unit | 10 | 130 | 17.8 | 4 | Positive |
| sp\|P33316\|DUT_HUMAN | TDIQIALPSGC[+57.0]YGR.light | FALSE | 775.89 | Unit | 1093.55 | Unit | 10 | 130 | 25.1 | 4 | Positive |
| sp\|P33316\|DUT_HUMAN | TDIQIALPSGC[+57.0]YGR.light | FALSE | 775.89 | Unit | 980.46 | Unit | 10 | 130 | 25.1 | 4 | Positive |
| sp\|P33316\|DUT_HUMAN | TDIQIALPSGC[+57.0]YGR.light | FALSE | 775.89 | Unit | 909.42 | Unit | 10 | 130 | 25.1 | 4 | Positive |
| sp\|P33316\|DUT_HUMAN | TDIQIALPSGC[+57.0]YGR.light | FALSE | 775.89 | Unit | 796.34 | Unit | 10 | 130 | 25.1 | 4 | Positive |
| sp\|P23588\|IF4B_HUMAN | EDDSSASTSQSTR.light | FALSE | 685.79 | Unit | 924.44 | Unit | 10 | 130 | 22.3 | 4 | Positive |
| sp\|P23588\|IF4B_HUMAN | EDDSSASTSQSTR.light | FALSE | 685.79 | Unit | 837.41 | Unit | 10 | 130 | 22.3 | 4 | Positive |
| sp\|P23588\|IF4B_HUMAN | EDDSSASTSQSTR.light | FALSE | 685.79 | Unit | 766.37 | Unit | 10 | 130 | 22.3 | 4 | Positive |
| sp\|P23588\|IF4B_HUMAN | EDDSSASTSQSTR.light | FALSE | 685.79 | Unit | 679.34 | Unit | 10 | 130 | 22.3 | 4 | Positive |
| sp\|P23588\|IF4B_HUMAN | TGSESSQTGTSTTSSR.light | FALSE | 787.35 | Unit | 1199.55 | Unit | 10 | 130 | 25.4 | 4 | Positive |
| sp\|P23588\|IF4B_HUMAN | TGSESSQTGTSTTSSR.light | FALSE | 787.35 | Unit | 1112.52 | Unit | 10 | 130 | 25.4 | 4 | Positive |
| sp\|P23588\|IF4B_HUMAN | TGSESSQTGTSTTSSR.light | FALSE | 787.35 | Unit | 897.43 | Unit | 10 | 130 | 25.4 | 4 | Positive |
| sp\|P23588\|IF4B_HUMAN | TGSESSQTGTSTTSSR.light | FALSE | 787.35 | Unit | 796.38 | Unit | 10 | 130 | 25.4 | 4 | Positive |
| sp\|P49327\|FAS_HUMAN | LGMLSPEGTC[+57.0]K.light | FALSE | 596.79 | Unit | 1079.49 | Unit | 10 | 130 | 19.5 | 4 | Positive |
| sp\|P49327\|FAS_HUMAN | LGMLSPEGTC[+57.0]K.light | FALSE | 596.79 | Unit | 891.42 | Unit | 10 | 130 | 19.5 | 4 | Positive |
| sp\|P49327\|FAS_HUMAN | LGMLSPEGTC[+57.0]K.light | FALSE | 596.79 | Unit | 778.34 | Unit | 10 | 130 | 19.5 | 4 | Positive |
| sp\|P49327\|FAS_HUMAN | LGMLSPEGTC[+57.0]K.light | FALSE | 596.79 | Unit | 691.31 | Unit | 10 | 130 | 19.5 | 4 | Positive |
| sp\|P49327\|FAS_HUMAN | SEGVVAVLLTK.light | FALSE | 558.34 | Unit | 899.59 | Unit | 10 | 130 | 18.3 | 4 | Positive |
| sp\|P49327\|FAS_HUMAN | SEGVVAVLLTK.light | FALSE | 558.34 | Unit | 743.50 | Unit | 10 | 130 | 18.3 | 4 | Positive |
| sp\|P49327\|FAS_HUMAN | SEGVVAVLLTK.light | FALSE | 558.34 | Unit | 644.43 | Unit | 10 | 130 | 18.3 | 4 | Positive |
| sp\|P49327\|FAS_HUMAN | SEGVVAVLLTK.light | FALSE | 558.34 | Unit | 147.11 | Unit | 10 | 130 | 18.3 | 4 | Positive |
| sp\|P02794\|FRIH_HUMAN | QNYHQDSEAAINR.light | FALSE | 773.36 | Unit | 1140.54 | Unit | 10 | 130 | 25 | 4 | Positive |
| sp\|P02794\|FRIH_HUMAN | QNYHQDSEAAINR.light | FALSE | 773.36 | Unit | 1003.48 | Unit | 10 | 130 | 25 | 4 | Positive |
| sp\|P02794\|FRIH_HUMAN | QNYHQDSEAAINR.light | FALSE | 773.36 | Unit | 875.42 | Unit | 10 | 130 | 25 | 4 | Positive |
| sp\|P02794\|FRIH_HUMAN | QNYHQDSEAAINR.light | FALSE | 773.36 | Unit | 760.39 | Unit | 10 | 130 | 25 | 4 | Positive |
| sp\|P02794\|FRIH_HUMAN | YFLHQSHEER.light | FALSE | 673.32 | Unit | 922.41 | Unit | 10 | 130 | 21.9 | 4 | Positive |
| sp\|P02794\|FRIH_HUMAN | YFLHQSHEER.light | FALSE | 673.32 | Unit | 785.35 | Unit | 10 | 130 | 21.9 | 4 | Positive |
| sp\|P02794\|FRIH_HUMAN | YFLHQSHEER.light | FALSE | 673.32 | Unit | 657.30 | Unit | 10 | 130 | 21.9 | 4 | Positive |
| sp\|P02794\|FRIH_HUMAN | YFLHQSHEER.light | FALSE | 673.32 | Unit | 175.12 | Unit | 10 | 130 | 21.9 | 4 | Positive |
| sp\|P40939\|ECHA_HUMAN | TLQEVTQLSQEAQR.light | FALSE | 815.92 | Unit | 1159.61 | Unit | 10 | 130 | 26.3 | 4 | Positive |
| sp\|P40939\|ECHA_HUMAN | TLQEVTQLSQEAQR.light | FALSE | 815.92 | Unit | 1060.54 | Unit | 10 | 130 | 26.3 | 4 | Positive |
| sp\|P40939\|ECHA_HUMAN | TLQEVTQLSQEAQR.light | FALSE | 815.92 | Unit | 831.43 | Unit | 10 | 130 | 26.3 | 4 | Positive |
| sp\|P40939\|ECHA_HUMAN | TLQEVTQLSQEAQR.light | FALSE | 815.92 | Unit | 718.35 | Unit | 10 | 130 | 26.3 | 4 | Positive |
| sp\|P40939\|ECHA_HUMAN | LTAYAMTIPFVR.light | FALSE | 691.88 | Unit | 934.52 | Unit | 10 | 130 | 22.4 | 4 | Positive |
| sp\|P40939\|ECHA_HUMAN | LTAYAMTIPFVR.light | FALSE | 691.88 | Unit | 863.48 | Unit | 10 | 130 | 22.4 | 4 | Positive |
| sp\|P40939\|ECHA_HUMAN | LTAYAMTIPFVR.light | FALSE | 691.88 | Unit | 732.44 | Unit | 10 | 130 | 22.4 | 4 | Positive |
| sp\|P40939\|ECHA_HUMAN | LTAYAMTIPFVR.light | FALSE | 691.88 | Unit | 518.31 | Unit | 10 | 130 | 22.4 | 4 | Positive |
| sp\|P08238\|HS90B_HUMAN | ADHGEPIGR.light | FALSE | 476.24 | Unit | 880.43 | Unit | 10 | 130 | 15.8 | 4 | Positive |
| sp\|P08238\|HS90B_HUMAN | ADHGEPIGR.light | FALSE | 476.24 | Unit | 765.40 | Unit | 10 | 130 | 15.8 | 4 | Positive |
| sp\|P08238\|HS90B_HUMAN | ADHGEPIGR.light | FALSE | 476.24 | Unit | 628.34 | Unit | 10 | 130 | 15.8 | 4 | Positive |
| sp\|P08238\|HS90B_HUMAN | ADHGEPIGR.light | FALSE | 476.24 | Unit | 442.28 | Unit | 10 | 130 | 15.8 | 4 | Positive |
| sp\|P08238\|HS90B_HUMAN | EISDDEAEEEK.light | FALSE | 647.27 | Unit | 1051.41 | Unit | 10 | 130 | 21.1 | 4 | Positive |
| sp\|P08238\|HS90B_HUMAN | EISDDEAEEEK.light | FALSE | 647.27 | Unit | 964.37 | Unit | 10 | 130 | 21.1 | 4 | Positive |
| sp\|P08238\|HS90B_HUMAN | EISDDEAEEEK.light | FALSE | 647.27 | Unit | 849.35 | Unit | 10 | 130 | 21.1 | 4 | Positive |
| sp\|P08238\|HS90B_HUMAN | EISDDEAEEEK.light | FALSE | 647.27 | Unit | 605.28 | Unit | 10 | 130 | 21.1 | 4 | Positive |
| sp\|P11021\|GRP78_HUMAN | ALSSQHQAR.light | FALSE | 499.26 | Unit | 813.40 | Unit | 10 | 130 | 16.5 | 4 | Positive |
| sp\|P11021\|GRP78_HUMAN | ALSSQHQAR.light | FALSE | 499.26 | Unit | 726.36 | Unit | 10 | 130 | 16.5 | 4 | Positive |
| sp\|P11021\|GRP78_HUMAN | ALSSQHQAR.light | FALSE | 499.26 | Unit | 511.27 | Unit | 10 | 130 | 16.5 | 4 | Positive |
| sp\|P11021\|GRP78_HUMAN | ALSSQHQAR.light | FALSE | 499.26 | Unit | 175.12 | Unit | 10 | 130 | 16.5 | 4 | Positive |
| sp\|P11021\|GRP78_HUMAN | SDIDEIVLVGGSTR.light | FALSE | 730.88 | Unit | 788.46 | Unit | 10 | 130 | 23.7 | 4 | Positive |
| sp\|P11021\|GRP78_HUMAN | SDIDEIVLVGGSTR.light | FALSE | 730.88 | Unit | 689.39 | Unit | 10 | 130 | 23.7 | 4 | Positive |
| sp\|P11021\|GRP78_HUMAN | SDIDEIVLVGGSTR.light | FALSE | 730.88 | Unit | 576.31 | Unit | 10 | 130 | 23.7 | 4 | Positive |
| sp\|P11021\|GRP78_HUMAN | SDIDEIVLVGGSTR.light | FALSE | 730.88 | Unit | 477.24 | Unit | 10 | 130 | 23.7 | 4 | Positive |
| sp\|P10809\|CH60_HUMAN | VGGTSDVEVNEK.light | FALSE | 617.30 | Unit | 1134.53 | Unit | 10 | 130 | 20.1 | 4 | Positive |
| sp\|P10809\|CH60_HUMAN | VGGTSDVEVNEK.light | FALSE | 617.30 | Unit | 919.44 | Unit | 10 | 130 | 20.1 | 4 | Positive |
| sp\|P10809\|CH60_HUMAN | VGGTSDVEVNEK.light | FALSE | 617.30 | Unit | 618.31 | Unit | 10 | 130 | 20.1 | 4 | Positive |
| sp\|P10809\|CH60_HUMAN | VGGTSDVEVNEK.light | FALSE | 617.30 | Unit | 390.20 | Unit | 10 | 130 | 20.1 | 4 | Positive |
| sp\|P10809\|CH60_HUMAN | VTDALNATR.light | FALSE | 480.76 | Unit | 861.44 | Unit | 10 | 130 | 15.9 | 4 | Positive |
| sp\|P10809\|CH60_HUMAN | VTDALNATR.light | FALSE | 480.76 | Unit | 760.39 | Unit | 10 | 130 | 15.9 | 4 | Positive |
| sp\|P10809\|CH60_HUMAN | VTDALNATR.light | FALSE | 480.76 | Unit | 574.33 | Unit | 10 | 130 | 15.9 | 4 | Positive |
| sp\|P10809\|CH60_HUMAN | VTDALNATR.light | FALSE | 480.76 | Unit | 461.25 | Unit | 10 | 130 | 15.9 | 4 | Positive |
| sp\|P14735\|IDE_HUMAN | VLLISDPTTDK.light | FALSE | 601.34 | Unit | 989.51 | Unit | 10 | 130 | 19.6 | 4 | Positive |
| sp\|P14735\|IDE_HUMAN | VLLISDPTTDK.light | FALSE | 601.34 | Unit | 876.43 | Unit | 10 | 130 | 19.6 | 4 | Positive |
| sp\|P14735\|IDE_HUMAN | VLLISDPTTDK.light | FALSE | 601.34 | Unit | 763.35 | Unit | 10 | 130 | 19.6 | 4 | Positive |
| sp\|P14735\|IDE_HUMAN | VLLISDPTTDK.light | FALSE | 601.34 | Unit | 561.29 | Unit | 10 | 130 | 19.6 | 4 | Positive |
| sp\|P14735\|IDE_HUMAN | ESLDDLTNLVVK.light | FALSE | 673.36 | Unit | 1016.56 | Unit | 10 | 130 | 21.9 | 4 | Positive |
| sp\|P14735\|IDE_HUMAN | ESLDDLTNLVVK.light | FALSE | 673.36 | Unit | 901.54 | Unit | 10 | 130 | 21.9 | 4 | Positive |
| sp\|P14735\|IDE_HUMAN | ESLDDLTNLVVK.light | FALSE | 673.36 | Unit | 673.42 | Unit | 10 | 130 | 21.9 | 4 | Positive |
| sp\|P14735\|IDE_HUMAN | ESLDDLTNLVVK.light | FALSE | 673.36 | Unit | 147.11 | Unit | 10 | 130 | 21.9 | 4 | Positive |
| sp\|P52292\|IMA1_HUMAN | TGVVPQLVK.light | FALSE | 470.79 | Unit | 782.51 | Unit | 10 | 130 | 15.6 | 4 | Positive |
| sp\|P52292\|IMA1_HUMAN | TGVVPQLVK.light | FALSE | 470.79 | Unit | 683.45 | Unit | 10 | 130 | 15.6 | 4 | Positive |
| sp\|P52292\|IMA1_HUMAN | TGVVPQLVK.light | FALSE | 470.79 | Unit | 584.38 | Unit | 10 | 130 | 15.6 | 4 | Positive |
| sp\|P52292\|IMA1_HUMAN | TGVVPQLVK.light | FALSE | 470.79 | Unit | 147.11 | Unit | 10 | 130 | 15.6 | 4 | Positive |
| sp\|P52292\|IMA1_HUMAN | IEALQNHENESVYK.light | FALSE | 837.41 | Unit | 1119.51 | Unit | 10 | 130 | 27 | 4 | Positive |
| sp\|P52292\|IMA1_HUMAN | IEALQNHENESVYK.light | FALSE | 837.41 | Unit | 1005.46 | Unit | 10 | 130 | 27 | 4 | Positive |
| sp\|P52292\|IMA1_HUMAN | IEALQNHENESVYK.light | FALSE | 837.41 | Unit | 310.18 | Unit | 10 | 130 | 27 | 4 | Positive |
| sp\|P52292\|IMA1_HUMAN | IEALQNHENESVYK.light | FALSE | 837.41 | Unit | 147.11 | Unit | 10 | 130 | 27 | 4 | Positive |
| sp\|P28838\|AMPL_HUMAN | AAGIDEQENWHEGK.light | FALSE | 792.36 | Unit | 899.40 | Unit | 10 | 130 | 25.6 | 4 | Positive |
| sp\|P28838\|AMPL_HUMAN | AAGIDEQENWHEGK.light | FALSE | 792.36 | Unit | 770.36 | Unit | 10 | 130 | 25.6 | 4 | Positive |
| sp\|P28838\|AMPL_HUMAN | AAGIDEQENWHEGK.light | FALSE | 792.36 | Unit | 470.24 | Unit | 10 | 130 | 25.6 | 4 | Positive |
| sp\|P28838\|AMPL_HUMAN | AAGIDEQENWHEGK.light | FALSE | 792.36 | Unit | 204.13 | Unit | 10 | 130 | 25.6 | 4 | Positive |
| sp\|P28838\|AMPL_HUMAN | GVLFASGQNLAR.light | FALSE | 616.84 | Unit | 963.50 | Unit | 10 | 130 | 20.1 | 4 | Positive |
| sp\|P28838\|AMPL_HUMAN | GVLFASGQNLAR.light | FALSE | 616.84 | Unit | 816.43 | Unit | 10 | 130 | 20.1 | 4 | Positive |
| sp\|P28838\|AMPL_HUMAN | GVLFASGQNLAR.light | FALSE | 616.84 | Unit | 745.40 | Unit | 10 | 130 | 20.1 | 4 | Positive |
| sp\|P28838\|AMPL_HUMAN | GVLFASGQNLAR.light | FALSE | 616.84 | Unit | 658.36 | Unit | 10 | 130 | 20.1 | 4 | Positive |
| sp\|P33991\|MCM4_HUMAN | LVIWGTDVNVAAC[+57.0]K.light | FALSE | 773.41 | Unit | 1034.49 | Unit | 10 | 130 | 25 | 4 | Positive |
| sp\|P33991\|MCM4_HUMAN | LVIWGTDVNVAAC[+57.0]K.light | FALSE | 773.41 | Unit | 662.33 | Unit | 10 | 130 | 25 | 4 | Positive |
| sp\|P33991\|MCM4_HUMAN | LVIWGTDVNVAAC[+57.0]K.light | FALSE | 773.41 | Unit | 449.22 | Unit | 10 | 130 | 25 | 4 | Positive |
| sp\|P33991\|MCM4_HUMAN | LVIWGTDVNVAAC[+57.0]K.light | FALSE | 773.41 | Unit | 147.11 | Unit | 10 | 130 | 25 | 4 | Positive |
| sp\|P33991\|MCM4_HUMAN | SQLLQYVYNLVPR.light | FALSE | 796.94 | Unit | 1151.62 | Unit | 10 | 130 | 25.7 | 4 | Positive |
| sp\|P33991\|MCM4_HUMAN | SQLLQYVYNLVPR.light | FALSE | 796.94 | Unit | 1023.56 | Unit | 10 | 130 | 25.7 | 4 | Positive |
| sp\|P33991\|MCM4_HUMAN | SQLLQYVYNLVPR.light | FALSE | 796.94 | Unit | 860.50 | Unit | 10 | 130 | 25.7 | 4 | Positive |
| sp\|P33991\|MCM4_HUMAN | SQLLQYVYNLVPR.light | FALSE | 796.94 | Unit | 272.17 | Unit | 10 | 130 | 25.7 | 4 | Positive |
| sp\|P55786\|PSA_HUMAN | DYFNVPYPLPK.light | FALSE | 676.85 | Unit | 927.53 | Unit | 10 | 130 | 22 | 4 | Positive |
| sp\|P55786\|PSA_HUMAN | DYFNVPYPLPK.light | FALSE | 676.85 | Unit | 714.42 | Unit | 10 | 130 | 22 | 4 | Positive |
| sp\|P55786\|PSA_HUMAN | DYFNVPYPLPK.light | FALSE | 676.85 | Unit | 454.30 | Unit | 10 | 130 | 22 | 4 | Positive |
| sp\|P55786\|PSA_HUMAN | DYFNVPYPLPK.light | FALSE | 676.85 | Unit | 244.17 | Unit | 10 | 130 | 22 | 4 | Positive |
| sp\|P55786\|PSA_HUMAN | DNWEELYNR.light | FALSE | 619.78 | Unit | 823.39 | Unit | 10 | 130 | 20.2 | 4 | Positive |
| sp\|P55786\|PSA_HUMAN | DNWEELYNR.light | FALSE | 619.78 | Unit | 694.35 | Unit | 10 | 130 | 20.2 | 4 | Positive |
| sp\|P55786\|PSA_HUMAN | DNWEELYNR.light | FALSE | 619.78 | Unit | 452.23 | Unit | 10 | 130 | 20.2 | 4 | Positive |
| sp\|P55786\|PSA_HUMAN | DNWEELYNR.light | FALSE | 619.78 | Unit | 175.12 | Unit | 10 | 130 | 20.2 | 4 | Positive |
| sp\|P06748\|NPM_HUMAN | VDNDENEHQLSLR.light | FALSE | 784.87 | Unit | 996.52 | Unit | 10 | 130 | 25.3 | 4 | Positive |
| sp\|P06748\|NPM_HUMAN | VDNDENEHQLSLR.light | FALSE | 784.87 | Unit | 753.44 | Unit | 10 | 130 | 25.3 | 4 | Positive |
| sp\|P06748\|NPM_HUMAN | VDNDENEHQLSLR.light | FALSE | 784.87 | Unit | 616.38 | Unit | 10 | 130 | 25.3 | 4 | Positive |
| sp\|P06748\|NPM_HUMAN | VDNDENEHQLSLR.light | FALSE | 784.87 | Unit | 375.24 | Unit | 10 | 130 | 25.3 | 4 | Positive |
| sp\|P06748\|NPM_HUMAN | MTDQEAIQDLWQWR.light | FALSE | 910.43 | Unit | 1144.59 | Unit | 10 | 130 | 29.2 | 4 | Positive |
| sp\|P06748\|NPM_HUMAN | MTDQEAIQDLWQWR.light | FALSE | 910.43 | Unit | 1031.51 | Unit | 10 | 130 | 29.2 | 4 | Positive |
| sp\|P06748\|NPM_HUMAN | MTDQEAIQDLWQWR.light | FALSE | 910.43 | Unit | 903.45 | Unit | 10 | 130 | 29.2 | 4 | Positive |
| sp\|P06748\|NPM_HUMAN | MTDQEAIQDLWQWR.light | FALSE | 910.43 | Unit | 489.26 | Unit | 10 | 130 | 29.2 | 4 | Positive |
| sp\|P12004\|PCNA_HUMAN | DLSHIGDAVVISC[+57.0]AK.light | FALSE | 792.91 | Unit | 1132.60 | Unit | 10 | 130 | 25.6 | 4 | Positive |
| sp\|P12004\|PCNA_HUMAN | DLSHIGDAVVISC[+57.0]AK.light | FALSE | 792.91 | Unit | 1019.52 | Unit | 10 | 130 | 25.6 | 4 | Positive |
| sp\|P12004\|PCNA_HUMAN | DLSHIGDAVVISC[+57.0]AK.light | FALSE | 792.91 | Unit | 578.30 | Unit | 10 | 130 | 25.6 | 4 | Positive |
| sp\|P12004\|PCNA_HUMAN | DLSHIGDAVVISC[+57.0]AK.light | FALSE | 792.91 | Unit | 465.21 | Unit | 10 | 130 | 25.6 | 4 | Positive |
| sp\|P12004\|PCNA_HUMAN | FSASGELGNGNIK.light | FALSE | 647.33 | Unit | 988.51 | Unit | 10 | 130 | 21.1 | 4 | Positive |
| sp\|P12004\|PCNA_HUMAN | FSASGELGNGNIK.light | FALSE | 647.33 | Unit | 901.47 | Unit | 10 | 130 | 21.1 | 4 | Positive |
| sp\|P12004\|PCNA_HUMAN | FSASGELGNGNIK.light | FALSE | 647.33 | Unit | 715.41 | Unit | 10 | 130 | 21.1 | 4 | Positive |
| sp\|P12004\|PCNA_HUMAN | FSASGELGNGNIK.light | FALSE | 647.33 | Unit | 602.33 | Unit | 10 | 130 | 21.1 | 4 | Positive |
| sp\|P30086\|PEBP1_HUMAN | LYTLVLTDPDAPSR.light | FALSE | 780.92 | Unit | 971.48 | Unit | 10 | 130 | 25.2 | 4 | Positive |
| sp\|P30086\|PEBP1_HUMAN | LYTLVLTDPDAPSR.light | FALSE | 780.92 | Unit | 858.40 | Unit | 10 | 130 | 25.2 | 4 | Positive |
| sp\|P30086\|PEBP1_HUMAN | LYTLVLTDPDAPSR.light | FALSE | 780.92 | Unit | 757.35 | Unit | 10 | 130 | 25.2 | 4 | Positive |
| sp\|P30086\|PEBP1_HUMAN | LYTLVLTDPDAPSR.light | FALSE | 780.92 | Unit | 642.32 | Unit | 10 | 130 | 25.2 | 4 | Positive |
| sp\|P30086\|PEBP1_HUMAN | YVWLVYEQDRPLK.light | FALSE | 854.96 | Unit | 1147.61 | Unit | 10 | 130 | 27.5 | 4 | Positive |
| sp\|P30086\|PEBP1_HUMAN | YVWLVYEQDRPLK.light | FALSE | 854.96 | Unit | 1048.54 | Unit | 10 | 130 | 27.5 | 4 | Positive |
| sp\|P30086\|PEBP1_HUMAN | YVWLVYEQDRPLK.light | FALSE | 854.96 | Unit | 885.48 | Unit | 10 | 130 | 27.5 | 4 | Positive |
| sp\|P30086\|PEBP1_HUMAN | YVWLVYEQDRPLK.light | FALSE | 854.96 | Unit | 147.11 | Unit | 10 | 130 | 27.5 | 4 | Positive |
| sp\|P35232\|PHB_HUMAN | NVPVITGSK.light | FALSE | 457.77 | Unit | 701.42 | Unit | 10 | 130 | 15.2 | 4 | Positive |
| sp\|P35232\|PHB_HUMAN | NVPVITGSK.light | FALSE | 457.77 | Unit | 505.30 | Unit | 10 | 130 | 15.2 | 4 | Positive |
| sp\|P35232\|PHB_HUMAN | NVPVITGSK.light | FALSE | 457.77 | Unit | 392.21 | Unit | 10 | 130 | 15.2 | 4 | Positive |
| sp\|P35232\|PHB_HUMAN | NVPVITGSK.light | FALSE | 457.77 | Unit | 147.11 | Unit | 10 | 130 | 15.2 | 4 | Positive |
| sp\|P35232\|PHB_HUMAN | AAIISAEGDSK.light | FALSE | 531.28 | Unit | 806.39 | Unit | 10 | 130 | 17.5 | 4 | Positive |
| sp\|P35232\|PHB_HUMAN | AAIISAEGDSK.light | FALSE | 531.28 | Unit | 693.30 | Unit | 10 | 130 | 17.5 | 4 | Positive |
| sp\|P35232\|PHB_HUMAN | AAIISAEGDSK.light | FALSE | 531.28 | Unit | 606.27 | Unit | 10 | 130 | 17.5 | 4 | Positive |
| sp\|P35232\|PHB_HUMAN | AAIISAEGDSK.light | FALSE | 531.28 | Unit | 234.14 | Unit | 10 | 130 | 17.5 | 4 | Positive |
| sp\|P00491\|PNPH_HUMAN | NTAEWLLSHTK.light | FALSE | 650.34 | Unit | 1084.58 | Unit | 10 | 130 | 21.2 | 4 | Positive |
| sp\|P00491\|PNPH_HUMAN | NTAEWLLSHTK.light | FALSE | 650.34 | Unit | 1013.54 | Unit | 10 | 130 | 21.2 | 4 | Positive |
| sp\|P00491\|PNPH_HUMAN | NTAEWLLSHTK.light | FALSE | 650.34 | Unit | 884.50 | Unit | 10 | 130 | 21.2 | 4 | Positive |
| sp\|P00491\|PNPH_HUMAN | NTAEWLLSHTK.light | FALSE | 650.34 | Unit | 698.42 | Unit | 10 | 130 | 21.2 | 4 | Positive |
| sp\|P00491\|PNPH_HUMAN | ANHEEVLAAGK.light | FALSE | 569.80 | Unit | 953.51 | Unit | 10 | 130 | 18.7 | 4 | Positive |
| sp\|P00491\|PNPH_HUMAN | ANHEEVLAAGK.light | FALSE | 569.80 | Unit | 275.17 | Unit | 10 | 130 | 18.7 | 4 | Positive |
| sp\|P00491\|PNPH_HUMAN | ANHEEVLAAGK.light | FALSE | 569.80 | Unit | 204.13 | Unit | 10 | 130 | 18.7 | 4 | Positive |
| sp\|P00491\|PNPH_HUMAN | ANHEEVLAAGK.light | FALSE | 569.80 | Unit | 147.11 | Unit | 10 | 130 | 18.7 | 4 | Positive |
| sp\|Q99460\|PSMD1_HUMAN | QAIGIALETR.light | FALSE | 536.31 | Unit | 872.52 | Unit | 10 | 130 | 17.6 | 4 | Positive |
| sp\|Q99460\|PSMD1_HUMAN | QAIGIALETR.light | FALSE | 536.31 | Unit | 759.44 | Unit | 10 | 130 | 17.6 | 4 | Positive |
| sp\|Q99460\|PSMD1_HUMAN | QAIGIALETR.light | FALSE | 536.31 | Unit | 589.33 | Unit | 10 | 130 | 17.6 | 4 | Positive |
| sp\|Q99460\|PSMD1_HUMAN | QAIGIALETR.light | FALSE | 536.31 | Unit | 276.17 | Unit | 10 | 130 | 17.6 | 4 | Positive |
| sp\|Q99460\|PSMD1_HUMAN | FTATASLGVIHK.light | FALSE | 622.85 | Unit | 996.58 | Unit | 10 | 130 | 20.3 | 4 | Positive |
| sp\|Q99460\|PSMD1_HUMAN | FTATASLGVIHK.light | FALSE | 622.85 | Unit | 925.55 | Unit | 10 | 130 | 20.3 | 4 | Positive |
| sp\|Q99460\|PSMD1_HUMAN | FTATASLGVIHK.light | FALSE | 622.85 | Unit | 824.50 | Unit | 10 | 130 | 20.3 | 4 | Positive |
| sp\|Q99460\|PSMD1_HUMAN | FTATASLGVIHK.light | FALSE | 622.85 | Unit | 753.46 | Unit | 10 | 130 | 20.3 | 4 | Positive |
| sp\|P43487\|RANG_HUMAN | FASENDLPEWK.light | FALSE | 668.31 | Unit | 1117.52 | Unit | 10 | 130 | 21.7 | 4 | Positive |
| sp\|P43487\|RANG_HUMAN | FASENDLPEWK.light | FALSE | 668.31 | Unit | 901.44 | Unit | 10 | 130 | 21.7 | 4 | Positive |
| sp\|P43487\|RANG_HUMAN | FASENDLPEWK.light | FALSE | 668.31 | Unit | 559.29 | Unit | 10 | 130 | 21.7 | 4 | Positive |
| sp\|P43487\|RANG_HUMAN | FASENDLPEWK.light | FALSE | 668.31 | Unit | 333.19 | Unit | 10 | 130 | 21.7 | 4 | Positive |
| sp\|P42677\|RS27_HUMAN | DLLHPSPEEEK.light | FALSE | 647.32 | Unit | 952.44 | Unit | 10 | 130 | 21.1 | 4 | Positive |
| sp\|P42677\|RS27_HUMAN | DLLHPSPEEEK.light | FALSE | 647.32 | Unit | 815.38 | Unit | 10 | 130 | 21.1 | 4 | Positive |
| sp\|P42677\|RS27_HUMAN | DLLHPSPEEEK.light | FALSE | 647.32 | Unit | 631.29 | Unit | 10 | 130 | 21.1 | 4 | Positive |
| sp\|P42677\|RS27_HUMAN | DLLHPSPEEEK.light | FALSE | 647.32 | Unit | 147.11 | Unit | 10 | 130 | 21.1 | 4 | Positive |
| sp\|P42677\|RS27_HUMAN | LVQSPNSYFMDVK.light | FALSE | 764.38 | Unit | 1187.54 | Unit | 10 | 130 | 24.7 | 4 | Positive |
| sp\|P42677\|RS27_HUMAN | LVQSPNSYFMDVK.light | FALSE | 764.38 | Unit | 1100.51 | Unit | 10 | 130 | 24.7 | 4 | Positive |
| sp\|P42677\|RS27_HUMAN | LVQSPNSYFMDVK.light | FALSE | 764.38 | Unit | 246.18 | Unit | 10 | 130 | 24.7 | 4 | Positive |
| sp\|P42677\|RS27_HUMAN | LVQSPNSYFMDVK.light | FALSE | 764.38 | Unit | 147.11 | Unit | 10 | 130 | 24.7 | 4 | Positive |
| sp\|P82979\|SARNP_HUMAN | FNVPVSLESK.light | FALSE | 560.31 | Unit | 858.49 | Unit | 10 | 130 | 18.4 | 4 | Positive |
| sp\|P82979\|SARNP_HUMAN | FNVPVSLESK.light | FALSE | 560.31 | Unit | 759.42 | Unit | 10 | 130 | 18.4 | 4 | Positive |
| sp\|P82979\|SARNP_HUMAN | FNVPVSLESK.light | FALSE | 560.31 | Unit | 563.30 | Unit | 10 | 130 | 18.4 | 4 | Positive |
| sp\|P82979\|SARNP_HUMAN | FNVPVSLESK.light | FALSE | 560.31 | Unit | 234.14 | Unit | 10 | 130 | 18.4 | 4 | Positive |
| sp\|P82979\|SARNP_HUMAN | FGLNVSSISR.light | FALSE | 540.30 | Unit | 762.41 | Unit | 10 | 130 | 17.7 | 4 | Positive |
| sp\|P82979\|SARNP_HUMAN | FGLNVSSISR.light | FALSE | 540.30 | Unit | 648.37 | Unit | 10 | 130 | 17.7 | 4 | Positive |
| sp\|P82979\|SARNP_HUMAN | FGLNVSSISR.light | FALSE | 540.30 | Unit | 549.30 | Unit | 10 | 130 | 17.7 | 4 | Positive |
| sp\|P82979\|SARNP_HUMAN | FGLNVSSISR.light | FALSE | 540.30 | Unit | 462.27 | Unit | 10 | 130 | 17.7 | 4 | Positive |
| sp\|P05141\|ADT2_HUMAN | GNLANVIR.light | FALSE | 428.75 | Unit | 685.44 | Unit | 10 | 130 | 14.3 | 4 | Positive |
| sp\|P05141\|ADT2_HUMAN | GNLANVIR.light | FALSE | 428.75 | Unit | 572.35 | Unit | 10 | 130 | 14.3 | 4 | Positive |
| sp\|P05141\|ADT2_HUMAN | GNLANVIR.light | FALSE | 428.75 | Unit | 501.31 | Unit | 10 | 130 | 14.3 | 4 | Positive |
| sp\|P05141\|ADT2_HUMAN | GNLANVIR.light | FALSE | 428.75 | Unit | 175.12 | Unit | 10 | 130 | 14.3 | 4 | Positive |
| sp\|P05141\|ADT2_HUMAN | QIFLGGVDK.light | FALSE | 488.78 | Unit | 735.40 | Unit | 10 | 130 | 16.2 | 4 | Positive |
| sp\|P05141\|ADT2_HUMAN | QIFLGGVDK.light | FALSE | 488.78 | Unit | 588.34 | Unit | 10 | 130 | 16.2 | 4 | Positive |
| sp\|P05141\|ADT2_HUMAN | QIFLGGVDK.light | FALSE | 488.78 | Unit | 475.25 | Unit | 10 | 130 | 16.2 | 4 | Positive |
| sp\|P05141\|ADT2_HUMAN | QIFLGGVDK.light | FALSE | 488.78 | Unit | 262.14 | Unit | 10 | 130 | 16.2 | 4 | Positive |
| sp\|P31948\|STIP1_HUMAN | EGLQNMEAR.light | FALSE | 524.25 | Unit | 748.34 | Unit | 10 | 130 | 17.3 | 4 | Positive |
| sp\|P31948\|STIP1_HUMAN | EGLQNMEAR.light | FALSE | 524.25 | Unit | 620.28 | Unit | 10 | 130 | 17.3 | 4 | Positive |
| sp\|P31948\|STIP1_HUMAN | EGLQNMEAR.light | FALSE | 524.25 | Unit | 246.16 | Unit | 10 | 130 | 17.3 | 4 | Positive |
| sp\|P31948\|STIP1_HUMAN | EGLQNMEAR.light | FALSE | 524.25 | Unit | 175.12 | Unit | 10 | 130 | 17.3 | 4 | Positive |
| sp\|P31948\|STIP1_HUMAN | DAIHFYNK.light | FALSE | 504.25 | Unit | 708.35 | Unit | 10 | 130 | 16.6 | 4 | Positive |
| sp\|P31948\|STIP1_HUMAN | DAIHFYNK.light | FALSE | 504.25 | Unit | 571.29 | Unit | 10 | 130 | 16.6 | 4 | Positive |
| sp\|P31948\|STIP1_HUMAN | DAIHFYNK.light | FALSE | 504.25 | Unit | 261.16 | Unit | 10 | 130 | 16.6 | 4 | Positive |
| sp\|P31948\|STIP1_HUMAN | DAIHFYNK.light | FALSE | 504.25 | Unit | 147.11 | Unit | 10 | 130 | 16.6 | 4 | Positive |
| sp\|P02787\|TRFE_HUMAN | EFQLFSSPHGK.light | FALSE | 638.82 | Unit | 872.46 | Unit | 10 | 130 | 20.8 | 4 | Positive |
| sp\|P02787\|TRFE_HUMAN | EFQLFSSPHGK.light | FALSE | 638.82 | Unit | 759.38 | Unit | 10 | 130 | 20.8 | 4 | Positive |
| sp\|P02787\|TRFE_HUMAN | EFQLFSSPHGK.light | FALSE | 638.82 | Unit | 612.31 | Unit | 10 | 130 | 20.8 | 4 | Positive |
| sp\|P02787\|TRFE_HUMAN | EFQLFSSPHGK.light | FALSE | 638.82 | Unit | 438.25 | Unit | 10 | 130 | 20.8 | 4 | Positive |
| sp\|P02787\|TRFE_HUMAN | APNHAVVTR.light | FALSE | 482.77 | Unit | 893.50 | Unit | 10 | 130 | 16 | 4 | Positive |
| sp\|P02787\|TRFE_HUMAN | APNHAVVTR.light | FALSE | 482.77 | Unit | 796.44 | Unit | 10 | 130 | 16 | 4 | Positive |
| sp\|P02787\|TRFE_HUMAN | APNHAVVTR.light | FALSE | 482.77 | Unit | 682.40 | Unit | 10 | 130 | 16 | 4 | Positive |
| sp\|P02787\|TRFE_HUMAN | APNHAVVTR.light | FALSE | 482.77 | Unit | 545.34 | Unit | 10 | 130 | 16 | 4 | Positive |
| sp\|P02786\|TFR1_HUMAN | ILNIFGVIK.light | FALSE | 508.83 | Unit | 903.57 | Unit | 10 | 130 | 16.8 | 4 | Positive |
| sp\|P02786\|TFR1_HUMAN | ILNIFGVIK.light | FALSE | 508.83 | Unit | 790.48 | Unit | 10 | 130 | 16.8 | 4 | Positive |
| sp\|P02786\|TFR1_HUMAN | ILNIFGVIK.light | FALSE | 508.83 | Unit | 563.36 | Unit | 10 | 130 | 16.8 | 4 | Positive |
| sp\|P02786\|TFR1_HUMAN | ILNIFGVIK.light | FALSE | 508.83 | Unit | 147.11 | Unit | 10 | 130 | 16.8 | 4 | Positive |
| sp\|P02786\|TFR1_HUMAN | AFTYINLDK.light | FALSE | 542.79 | Unit | 866.46 | Unit | 10 | 130 | 17.8 | 4 | Positive |
| sp\|P02786\|TFR1_HUMAN | AFTYINLDK.light | FALSE | 542.79 | Unit | 765.41 | Unit | 10 | 130 | 17.8 | 4 | Positive |
| sp\|P02786\|TFR1_HUMAN | AFTYINLDK.light | FALSE | 542.79 | Unit | 602.35 | Unit | 10 | 130 | 17.8 | 4 | Positive |
| sp\|P02786\|TFR1_HUMAN | AFTYINLDK.light | FALSE | 542.79 | Unit | 489.27 | Unit | 10 | 130 | 17.8 | 4 | Positive |
| sp\|P06753\|TPM3_HUMAN | QLEDELAAMQK.light | FALSE | 638.32 | Unit | 1034.48 | Unit | 10 | 130 | 20.8 | 4 | Positive |
| sp\|P06753\|TPM3_HUMAN | QLEDELAAMQK.light | FALSE | 638.32 | Unit | 661.37 | Unit | 10 | 130 | 20.8 | 4 | Positive |
| sp\|P06753\|TPM3_HUMAN | QLEDELAAMQK.light | FALSE | 638.32 | Unit | 548.29 | Unit | 10 | 130 | 20.8 | 4 | Positive |
| sp\|P06753\|TPM3_HUMAN | QLEDELAAMQK.light | FALSE | 638.32 | Unit | 477.25 | Unit | 10 | 130 | 20.8 | 4 | Positive |
| sp\|P30536\|TSPO_HUMAN | FVHGEGLR.light | FALSE | 457.75 | Unit | 767.42 | Unit | 10 | 130 | 15.2 | 4 | Positive |
| sp\|P30536\|TSPO_HUMAN | FVHGEGLR.light | FALSE | 457.75 | Unit | 668.35 | Unit | 10 | 130 | 15.2 | 4 | Positive |
| sp\|P30536\|TSPO_HUMAN | FVHGEGLR.light | FALSE | 457.75 | Unit | 531.29 | Unit | 10 | 130 | 15.2 | 4 | Positive |
| sp\|P30536\|TSPO_HUMAN | FVHGEGLR.light | FALSE | 457.75 | Unit | 175.12 | Unit | 10 | 130 | 15.2 | 4 | Positive |
| sp\|P30536\|TSPO_HUMAN | ELGGFTEK.light | FALSE | 440.72 | Unit | 638.31 | Unit | 10 | 130 | 14.7 | 4 | Positive |
| sp\|P30536\|TSPO_HUMAN | ELGGFTEK.light | FALSE | 440.72 | Unit | 524.27 | Unit | 10 | 130 | 14.7 | 4 | Positive |
| sp\|P30536\|TSPO_HUMAN | ELGGFTEK.light | FALSE | 440.72 | Unit | 377.20 | Unit | 10 | 130 | 14.7 | 4 | Positive |
| sp\|P30536\|TSPO_HUMAN | ELGGFTEK.light | FALSE | 440.72 | Unit | 147.11 | Unit | 10 | 130 | 14.7 | 4 | Positive |

**Table S.4. Transition List for AS Panel**

| **Compound Group** | **Compound Name** | **ISTD?** | **Precursor Ion** | **MS1 Res** | **Product Ion** | **MS2 Res** | **Dwell** | **Fragmentor** | **Collision Energy** | **Cell Accelerator Voltage** | **Polarity** |
| --- | --- | --- | --- | --- | --- | --- | --- | --- | --- | --- | --- |
| sp\|P04792\|HSPB1_HUMAN | QLSSGVSEIR.light | FALSE | 538.29 | Unit | 834.43 | Unit | 10 | 130 | 17.7 | 4 | Positive |
| sp\|P04792\|HSPB1_HUMAN | QLSSGVSEIR.light | FALSE | 538.29 | Unit | 660.37 | Unit | 10 | 130 | 17.7 | 4 | Positive |
| sp\|P04792\|HSPB1_HUMAN | QLSSGVSEIR.light | FALSE | 538.29 | Unit | 504.28 | Unit | 10 | 130 | 17.7 | 4 | Positive |
| sp\|P04792\|HSPB1_HUMAN | QLSSGVSEIR.light | FALSE | 538.29 | Unit | 288.20 | Unit | 10 | 130 | 17.7 | 4 | Positive |
| sp\|P04792\|HSPB1_HUMAN | DGVVEITGK.light | FALSE | 459.25 | Unit | 646.38 | Unit | 10 | 130 | 15.2 | 4 | Positive |
| sp\|P04792\|HSPB1_HUMAN | DGVVEITGK.light | FALSE | 459.25 | Unit | 547.31 | Unit | 10 | 130 | 15.2 | 4 | Positive |
| sp\|P04792\|HSPB1_HUMAN | DGVVEITGK.light | FALSE | 459.25 | Unit | 305.18 | Unit | 10 | 130 | 15.2 | 4 | Positive |
| sp\|P04792\|HSPB1_HUMAN | DGVVEITGK.light | FALSE | 459.25 | Unit | 204.13 | Unit | 10 | 130 | 15.2 | 4 | Positive |
| sp\|P07099\|HYEP_HUMAN | DVELLYPVK.light | FALSE | 538.31 | Unit | 861.51 | Unit | 10 | 130 | 17.7 | 4 | Positive |
| sp\|P07099\|HYEP_HUMAN | DVELLYPVK.light | FALSE | 538.31 | Unit | 506.30 | Unit | 10 | 130 | 17.7 | 4 | Positive |
| sp\|P07099\|HYEP_HUMAN | DVELLYPVK.light | FALSE | 538.31 | Unit | 343.23 | Unit | 10 | 130 | 17.7 | 4 | Positive |
| sp\|P07099\|HYEP_HUMAN | DVELLYPVK.light | FALSE | 538.31 | Unit | 147.11 | Unit | 10 | 130 | 17.7 | 4 | Positive |
| sp\|P07099\|HYEP_HUMAN | YLEDGGLER.light | FALSE | 526.26 | Unit | 888.44 | Unit | 10 | 130 | 17.3 | 4 | Positive |
| sp\|P07099\|HYEP_HUMAN | YLEDGGLER.light | FALSE | 526.26 | Unit | 775.36 | Unit | 10 | 130 | 17.3 | 4 | Positive |
| sp\|P07099\|HYEP_HUMAN | YLEDGGLER.light | FALSE | 526.26 | Unit | 646.32 | Unit | 10 | 130 | 17.3 | 4 | Positive |
| sp\|P07099\|HYEP_HUMAN | YLEDGGLER.light | FALSE | 526.26 | Unit | 531.29 | Unit | 10 | 130 | 17.3 | 4 | Positive |
| sp\|P07339\|CATD_HUMAN | QVFGEATK.light | FALSE | 440.23 | Unit | 652.33 | Unit | 10 | 130 | 14.6 | 4 | Positive |
| sp\|P07339\|CATD_HUMAN | QVFGEATK.light | FALSE | 440.23 | Unit | 505.26 | Unit | 10 | 130 | 14.6 | 4 | Positive |
| sp\|P07339\|CATD_HUMAN | QVFGEATK.light | FALSE | 440.23 | Unit | 248.16 | Unit | 10 | 130 | 14.6 | 4 | Positive |
| sp\|P07339\|CATD_HUMAN | QVFGEATK.light | FALSE | 440.23 | Unit | 147.11 | Unit | 10 | 130 | 14.6 | 4 | Positive |
| sp\|P07339\|CATD_HUMAN | VGFAEAAR.light | FALSE | 410.72 | Unit | 721.36 | Unit | 10 | 130 | 13.7 | 4 | Positive |
| sp\|P07339\|CATD_HUMAN | VGFAEAAR.light | FALSE | 410.72 | Unit | 664.34 | Unit | 10 | 130 | 13.7 | 4 | Positive |
| sp\|P07339\|CATD_HUMAN | VGFAEAAR.light | FALSE | 410.72 | Unit | 517.27 | Unit | 10 | 130 | 13.7 | 4 | Positive |
| sp\|P07339\|CATD_HUMAN | VGFAEAAR.light | FALSE | 410.72 | Unit | 446.24 | Unit | 10 | 130 | 13.7 | 4 | Positive |
| sp\|O75369\|FLNB_HUMAN | VLPTYDASK.light | FALSE | 497.27 | Unit | 781.37 | Unit | 10 | 130 | 16.4 | 4 | Positive |
| sp\|O75369\|FLNB_HUMAN | VLPTYDASK.light | FALSE | 497.27 | Unit | 684.32 | Unit | 10 | 130 | 16.4 | 4 | Positive |
| sp\|O75369\|FLNB_HUMAN | VLPTYDASK.light | FALSE | 497.27 | Unit | 583.27 | Unit | 10 | 130 | 16.4 | 4 | Positive |
| sp\|O75369\|FLNB_HUMAN | VLPTYDASK.light | FALSE | 497.27 | Unit | 147.11 | Unit | 10 | 130 | 16.4 | 4 | Positive |
| sp\|O75369\|FLNB_HUMAN | LVSPGSANETSSILVESVTR.light | FALSE | 1023.54 | Unit | 1191.66 | Unit | 10 | 130 | 32.7 | 4 | Positive |
| sp\|O75369\|FLNB_HUMAN | LVSPGSANETSSILVESVTR.light | FALSE | 1023.54 | Unit | 1090.61 | Unit | 10 | 130 | 32.7 | 4 | Positive |
| sp\|O75369\|FLNB_HUMAN | LVSPGSANETSSILVESVTR.light | FALSE | 1023.54 | Unit | 591.31 | Unit | 10 | 130 | 32.7 | 4 | Positive |
| sp\|O75369\|FLNB_HUMAN | LVSPGSANETSSILVESVTR.light | FALSE | 1023.54 | Unit | 462.27 | Unit | 10 | 130 | 32.7 | 4 | Positive |
| sp\|P02794\|FRIH_HUMAN | QNYHQDSEAAINR.light | FALSE | 773.36 | Unit | 1140.54 | Unit | 10 | 130 | 25 | 4 | Positive |
| sp\|P02794\|FRIH_HUMAN | QNYHQDSEAAINR.light | FALSE | 773.36 | Unit | 1003.48 | Unit | 10 | 130 | 25 | 4 | Positive |
| sp\|P02794\|FRIH_HUMAN | QNYHQDSEAAINR.light | FALSE | 773.36 | Unit | 875.42 | Unit | 10 | 130 | 25 | 4 | Positive |
| sp\|P02794\|FRIH_HUMAN | QNYHQDSEAAINR.light | FALSE | 773.36 | Unit | 760.39 | Unit | 10 | 130 | 25 | 4 | Positive |
| sp\|P02794\|FRIH_HUMAN | NVNQSLLELHK.light | FALSE | 647.86 | Unit | 1081.60 | Unit | 10 | 130 | 21.1 | 4 | Positive |
| sp\|P02794\|FRIH_HUMAN | NVNQSLLELHK.light | FALSE | 647.86 | Unit | 839.50 | Unit | 10 | 130 | 21.1 | 4 | Positive |
| sp\|P02794\|FRIH_HUMAN | NVNQSLLELHK.light | FALSE | 647.86 | Unit | 639.38 | Unit | 10 | 130 | 21.1 | 4 | Positive |
| sp\|P02794\|FRIH_HUMAN | NVNQSLLELHK.light | FALSE | 647.86 | Unit | 284.17 | Unit | 10 | 130 | 21.1 | 4 | Positive |
| sp\|P04080\|CYTB_HUMAN | SQVVAGTNYFIK.light | FALSE | 663.86 | Unit | 1111.61 | Unit | 10 | 130 | 21.6 | 4 | Positive |
| sp\|P04080\|CYTB_HUMAN | SQVVAGTNYFIK.light | FALSE | 663.86 | Unit | 1012.55 | Unit | 10 | 130 | 21.6 | 4 | Positive |
| sp\|P04080\|CYTB_HUMAN | SQVVAGTNYFIK.light | FALSE | 663.86 | Unit | 913.48 | Unit | 10 | 130 | 21.6 | 4 | Positive |
| sp\|P04080\|CYTB_HUMAN | SQVVAGTNYFIK.light | FALSE | 663.86 | Unit | 842.44 | Unit | 10 | 130 | 21.6 | 4 | Positive |
| sp\|P04080\|CYTB_HUMAN | VHVGDEDFVHLR.light | FALSE | 711.86 | Unit | 1186.59 | Unit | 10 | 130 | 23.1 | 4 | Positive |
| sp\|P04080\|CYTB_HUMAN | VHVGDEDFVHLR.light | FALSE | 711.86 | Unit | 1087.52 | Unit | 10 | 130 | 23.1 | 4 | Positive |
| sp\|P04080\|CYTB_HUMAN | VHVGDEDFVHLR.light | FALSE | 711.86 | Unit | 915.47 | Unit | 10 | 130 | 23.1 | 4 | Positive |
| sp\|P04080\|CYTB_HUMAN | VHVGDEDFVHLR.light | FALSE | 711.86 | Unit | 671.40 | Unit | 10 | 130 | 23.1 | 4 | Positive |
| sp\|Q7KZF4\|SND1_HUMAN | DYVAPTANLDQK.light | FALSE | 667.83 | Unit | 957.50 | Unit | 10 | 130 | 21.7 | 4 | Positive |
| sp\|Q7KZF4\|SND1_HUMAN | DYVAPTANLDQK.light | FALSE | 667.83 | Unit | 886.46 | Unit | 10 | 130 | 21.7 | 4 | Positive |
| sp\|Q7KZF4\|SND1_HUMAN | DYVAPTANLDQK.light | FALSE | 667.83 | Unit | 688.36 | Unit | 10 | 130 | 21.7 | 4 | Positive |
| sp\|Q7KZF4\|SND1_HUMAN | DYVAPTANLDQK.light | FALSE | 667.83 | Unit | 275.17 | Unit | 10 | 130 | 21.7 | 4 | Positive |
| sp\|Q7KZF4\|SND1_HUMAN | EVLPSTR.light | FALSE | 401.23 | Unit | 573.34 | Unit | 10 | 130 | 13.4 | 4 | Positive |
| sp\|Q7KZF4\|SND1_HUMAN | EVLPSTR.light | FALSE | 401.23 | Unit | 460.25 | Unit | 10 | 130 | 13.4 | 4 | Positive |
| sp\|Q7KZF4\|SND1_HUMAN | EVLPSTR.light | FALSE | 401.23 | Unit | 363.20 | Unit | 10 | 130 | 13.4 | 4 | Positive |
| sp\|Q7KZF4\|SND1_HUMAN | EVLPSTR.light | FALSE | 401.23 | Unit | 175.12 | Unit | 10 | 130 | 13.4 | 4 | Positive |
| sp\|P13667\|PDIA4_HUMAN | DGDDVIIIGVFK.light | FALSE | 645.85 | Unit | 789.52 | Unit | 10 | 130 | 21 | 4 | Positive |
| sp\|P13667\|PDIA4_HUMAN | DGDDVIIIGVFK.light | FALSE | 645.85 | Unit | 676.44 | Unit | 10 | 130 | 21 | 4 | Positive |
| sp\|P13667\|PDIA4_HUMAN | DGDDVIIIGVFK.light | FALSE | 645.85 | Unit | 294.18 | Unit | 10 | 130 | 21 | 4 | Positive |
| sp\|P13667\|PDIA4_HUMAN | DGDDVIIIGVFK.light | FALSE | 645.85 | Unit | 147.11 | Unit | 10 | 130 | 21 | 4 | Positive |
| sp\|P13667\|PDIA4_HUMAN | QLEPVYNSLAK.light | FALSE | 631.34 | Unit | 1020.54 | Unit | 10 | 130 | 20.6 | 4 | Positive |
| sp\|P13667\|PDIA4_HUMAN | QLEPVYNSLAK.light | FALSE | 631.34 | Unit | 891.49 | Unit | 10 | 130 | 20.6 | 4 | Positive |
| sp\|P13667\|PDIA4_HUMAN | QLEPVYNSLAK.light | FALSE | 631.34 | Unit | 695.37 | Unit | 10 | 130 | 20.6 | 4 | Positive |
| sp\|P13667\|PDIA4_HUMAN | QLEPVYNSLAK.light | FALSE | 631.34 | Unit | 532.31 | Unit | 10 | 130 | 20.6 | 4 | Positive |
| sp\|O75874\|IDHC_HUMAN | SIEDFAHSSFQMALSK.light | FALSE | 899.43 | Unit | 1135.56 | Unit | 10 | 130 | 28.9 | 4 | Positive |
| sp\|O75874\|IDHC_HUMAN | SIEDFAHSSFQMALSK.light | FALSE | 899.43 | Unit | 998.50 | Unit | 10 | 130 | 28.9 | 4 | Positive |
| sp\|O75874\|IDHC_HUMAN | SIEDFAHSSFQMALSK.light | FALSE | 899.43 | Unit | 234.14 | Unit | 10 | 130 | 28.9 | 4 | Positive |
| sp\|O75874\|IDHC_HUMAN | SIEDFAHSSFQMALSK.light | FALSE | 899.43 | Unit | 147.11 | Unit | 10 | 130 | 28.9 | 4 | Positive |
| sp\|O75874\|IDHC_HUMAN | TVEAEAAHGTVTR.light | FALSE | 671.34 | Unit | 1141.56 | Unit | 10 | 130 | 21.8 | 4 | Positive |
| sp\|O75874\|IDHC_HUMAN | TVEAEAAHGTVTR.light | FALSE | 671.34 | Unit | 1012.52 | Unit | 10 | 130 | 21.8 | 4 | Positive |
| sp\|O75874\|IDHC_HUMAN | TVEAEAAHGTVTR.light | FALSE | 671.34 | Unit | 812.44 | Unit | 10 | 130 | 21.8 | 4 | Positive |
| sp\|O75874\|IDHC_HUMAN | TVEAEAAHGTVTR.light | FALSE | 671.34 | Unit | 670.36 | Unit | 10 | 130 | 21.8 | 4 | Positive |
| sp\|P07195\|LDHB_HUMAN | LIAPVAEEEATVPNNK.light | FALSE | 847.95 | Unit | 1001.49 | Unit | 10 | 130 | 27.3 | 4 | Positive |
| sp\|P07195\|LDHB_HUMAN | LIAPVAEEEATVPNNK.light | FALSE | 847.95 | Unit | 743.40 | Unit | 10 | 130 | 27.3 | 4 | Positive |
| sp\|P07195\|LDHB_HUMAN | LIAPVAEEEATVPNNK.light | FALSE | 847.95 | Unit | 571.32 | Unit | 10 | 130 | 27.3 | 4 | Positive |
| sp\|P07195\|LDHB_HUMAN | LIAPVAEEEATVPNNK.light | FALSE | 847.95 | Unit | 472.25 | Unit | 10 | 130 | 27.3 | 4 | Positive |
| sp\|P07195\|LDHB_HUMAN | DYSVTANSK.light | FALSE | 492.74 | Unit | 706.37 | Unit | 10 | 130 | 16.3 | 4 | Positive |
| sp\|P07195\|LDHB_HUMAN | DYSVTANSK.light | FALSE | 492.74 | Unit | 520.27 | Unit | 10 | 130 | 16.3 | 4 | Positive |
| sp\|P07195\|LDHB_HUMAN | DYSVTANSK.light | FALSE | 492.74 | Unit | 348.19 | Unit | 10 | 130 | 16.3 | 4 | Positive |
| sp\|P07195\|LDHB_HUMAN | DYSVTANSK.light | FALSE | 492.74 | Unit | 147.11 | Unit | 10 | 130 | 16.3 | 4 | Positive |
| sp\|P13010\|XRCC5_HUMAN | LTIGSNLSIR.light | FALSE | 537.32 | Unit | 859.50 | Unit | 10 | 130 | 17.7 | 4 | Positive |
| sp\|P13010\|XRCC5_HUMAN | LTIGSNLSIR.light | FALSE | 537.32 | Unit | 746.42 | Unit | 10 | 130 | 17.7 | 4 | Positive |
| sp\|P13010\|XRCC5_HUMAN | LTIGSNLSIR.light | FALSE | 537.32 | Unit | 689.39 | Unit | 10 | 130 | 17.7 | 4 | Positive |
| sp\|P13010\|XRCC5_HUMAN | LTIGSNLSIR.light | FALSE | 537.32 | Unit | 175.12 | Unit | 10 | 130 | 17.7 | 4 | Positive |
| sp\|P13010\|XRCC5_HUMAN | EDIIQGFR.light | FALSE | 489.26 | Unit | 733.44 | Unit | 10 | 130 | 16.2 | 4 | Positive |
| sp\|P13010\|XRCC5_HUMAN | EDIIQGFR.light | FALSE | 489.26 | Unit | 620.35 | Unit | 10 | 130 | 16.2 | 4 | Positive |
| sp\|P13010\|XRCC5_HUMAN | EDIIQGFR.light | FALSE | 489.26 | Unit | 507.27 | Unit | 10 | 130 | 16.2 | 4 | Positive |
| sp\|P13010\|XRCC5_HUMAN | EDIIQGFR.light | FALSE | 489.26 | Unit | 379.21 | Unit | 10 | 130 | 16.2 | 4 | Positive |
| sp\|P21333\|FLNA_HUMAN | LDVQFSGLTK.light | FALSE | 554.31 | Unit | 994.52 | Unit | 10 | 130 | 18.2 | 4 | Positive |
| sp\|P21333\|FLNA_HUMAN | LDVQFSGLTK.light | FALSE | 554.31 | Unit | 879.49 | Unit | 10 | 130 | 18.2 | 4 | Positive |
| sp\|P21333\|FLNA_HUMAN | LDVQFSGLTK.light | FALSE | 554.31 | Unit | 780.43 | Unit | 10 | 130 | 18.2 | 4 | Positive |
| sp\|P21333\|FLNA_HUMAN | LDVQFSGLTK.light | FALSE | 554.31 | Unit | 652.37 | Unit | 10 | 130 | 18.2 | 4 | Positive |
| sp\|P21333\|FLNA_HUMAN | DAGEGLLAVQITDPEGKPK.light | FALSE | 969.51 | Unit | 1112.59 | Unit | 10 | 130 | 31.1 | 4 | Positive |
| sp\|P21333\|FLNA_HUMAN | DAGEGLLAVQITDPEGKPK.light | FALSE | 969.51 | Unit | 871.45 | Unit | 10 | 130 | 31.1 | 4 | Positive |
| sp\|P21333\|FLNA_HUMAN | DAGEGLLAVQITDPEGKPK.light | FALSE | 969.51 | Unit | 655.38 | Unit | 10 | 130 | 31.1 | 4 | Positive |
| sp\|P21333\|FLNA_HUMAN | DAGEGLLAVQITDPEGKPK.light | FALSE | 969.51 | Unit | 244.17 | Unit | 10 | 130 | 31.1 | 4 | Positive |
| sp\|P35232\|PHB_HUMAN | DLQNVNITLR.light | FALSE | 593.33 | Unit | 829.49 | Unit | 10 | 130 | 19.4 | 4 | Positive |
| sp\|P35232\|PHB_HUMAN | DLQNVNITLR.light | FALSE | 593.33 | Unit | 715.45 | Unit | 10 | 130 | 19.4 | 4 | Positive |
| sp\|P35232\|PHB_HUMAN | DLQNVNITLR.light | FALSE | 593.33 | Unit | 616.38 | Unit | 10 | 130 | 19.4 | 4 | Positive |
| sp\|P35232\|PHB_HUMAN | DLQNVNITLR.light | FALSE | 593.33 | Unit | 389.25 | Unit | 10 | 130 | 19.4 | 4 | Positive |
| sp\|P35232\|PHB_HUMAN | VLPSITTEILK.light | FALSE | 607.37 | Unit | 1001.59 | Unit | 10 | 130 | 19.8 | 4 | Positive |
| sp\|P35232\|PHB_HUMAN | VLPSITTEILK.light | FALSE | 607.37 | Unit | 904.53 | Unit | 10 | 130 | 19.8 | 4 | Positive |
| sp\|P35232\|PHB_HUMAN | VLPSITTEILK.light | FALSE | 607.37 | Unit | 704.42 | Unit | 10 | 130 | 19.8 | 4 | Positive |
| sp\|P35232\|PHB_HUMAN | VLPSITTEILK.light | FALSE | 607.37 | Unit | 502.32 | Unit | 10 | 130 | 19.8 | 4 | Positive |
| sp\|P02786\|TFR1_HUMAN | LDSTDFTGTIK.light | FALSE | 599.30 | Unit | 1084.52 | Unit | 10 | 130 | 19.6 | 4 | Positive |
| sp\|P02786\|TFR1_HUMAN | LDSTDFTGTIK.light | FALSE | 599.30 | Unit | 969.49 | Unit | 10 | 130 | 19.6 | 4 | Positive |
| sp\|P02786\|TFR1_HUMAN | LDSTDFTGTIK.light | FALSE | 599.30 | Unit | 781.41 | Unit | 10 | 130 | 19.6 | 4 | Positive |
| sp\|P02786\|TFR1_HUMAN | LDSTDFTGTIK.light | FALSE | 599.30 | Unit | 666.38 | Unit | 10 | 130 | 19.6 | 4 | Positive |
| sp\|P02786\|TFR1_HUMAN | DSAQNSVIIVDK.light | FALSE | 644.84 | Unit | 887.52 | Unit | 10 | 130 | 21 | 4 | Positive |
| sp\|P02786\|TFR1_HUMAN | DSAQNSVIIVDK.light | FALSE | 644.84 | Unit | 474.29 | Unit | 10 | 130 | 21 | 4 | Positive |
| sp\|P02786\|TFR1_HUMAN | DSAQNSVIIVDK.light | FALSE | 644.84 | Unit | 361.21 | Unit | 10 | 130 | 21 | 4 | Positive |
| sp\|P02786\|TFR1_HUMAN | DSAQNSVIIVDK.light | FALSE | 644.84 | Unit | 262.14 | Unit | 10 | 130 | 21 | 4 | Positive |
| sp\|Q8NCW5\|NNRE_HUMAN | LFGYEPTIYYPK.light | FALSE | 745.88 | Unit | 1010.52 | Unit | 10 | 130 | 24.1 | 4 | Positive |
| sp\|Q8NCW5\|NNRE_HUMAN | LFGYEPTIYYPK.light | FALSE | 745.88 | Unit | 881.48 | Unit | 10 | 130 | 24.1 | 4 | Positive |
| sp\|Q8NCW5\|NNRE_HUMAN | LFGYEPTIYYPK.light | FALSE | 745.88 | Unit | 407.23 | Unit | 10 | 130 | 24.1 | 4 | Positive |
| sp\|Q8NCW5\|NNRE_HUMAN | LFGYEPTIYYPK.light | FALSE | 745.88 | Unit | 244.17 | Unit | 10 | 130 | 24.1 | 4 | Positive |
| sp\|Q8NCW5\|NNRE_HUMAN | YHYLGGR.light | FALSE | 433.22 | Unit | 702.37 | Unit | 10 | 130 | 14.4 | 4 | Positive |
| sp\|Q8NCW5\|NNRE_HUMAN | YHYLGGR.light | FALSE | 433.22 | Unit | 565.31 | Unit | 10 | 130 | 14.4 | 4 | Positive |
| sp\|Q8NCW5\|NNRE_HUMAN | YHYLGGR.light | FALSE | 433.22 | Unit | 402.25 | Unit | 10 | 130 | 14.4 | 4 | Positive |
| sp\|Q8NCW5\|NNRE_HUMAN | YHYLGGR.light | FALSE | 433.22 | Unit | 175.12 | Unit | 10 | 130 | 14.4 | 4 | Positive |
| sp\|O15439\|MRP4_HUMAN | VIQPIFLGK.light | FALSE | 507.82 | Unit | 674.42 | Unit | 10 | 130 | 16.7 | 4 | Positive |
| sp\|O15439\|MRP4_HUMAN | VIQPIFLGK.light | FALSE | 507.82 | Unit | 464.29 | Unit | 10 | 130 | 16.7 | 4 | Positive |
| sp\|O15439\|MRP4_HUMAN | VIQPIFLGK.light | FALSE | 507.82 | Unit | 317.22 | Unit | 10 | 130 | 16.7 | 4 | Positive |
| sp\|O15439\|MRP4_HUMAN | VIQPIFLGK.light | FALSE | 507.82 | Unit | 204.13 | Unit | 10 | 130 | 16.7 | 4 | Positive |
| sp\|O15439\|MRP4_HUMAN | SGIDFGSLLK.light | FALSE | 518.79 | Unit | 779.43 | Unit | 10 | 130 | 17.1 | 4 | Positive |
| sp\|O15439\|MRP4_HUMAN | SGIDFGSLLK.light | FALSE | 518.79 | Unit | 664.40 | Unit | 10 | 130 | 17.1 | 4 | Positive |
| sp\|O15439\|MRP4_HUMAN | SGIDFGSLLK.light | FALSE | 518.79 | Unit | 517.33 | Unit | 10 | 130 | 17.1 | 4 | Positive |
| sp\|O15439\|MRP4_HUMAN | SGIDFGSLLK.light | FALSE | 518.79 | Unit | 147.11 | Unit | 10 | 130 | 17.1 | 4 | Positive |
| sp\|Q01105\|SET_HUMAN | EQQEAIEHIDEVQNEIDR.light | FALSE | 1098.01 | Unit | 1117.51 | Unit | 10 | 130 | 35 | 4 | Positive |
| sp\|Q01105\|SET_HUMAN | EQQEAIEHIDEVQNEIDR.light | FALSE | 1098.01 | Unit | 1002.49 | Unit | 10 | 130 | 35 | 4 | Positive |
| sp\|Q01105\|SET_HUMAN | EQQEAIEHIDEVQNEIDR.light | FALSE | 1098.01 | Unit | 646.32 | Unit | 10 | 130 | 35 | 4 | Positive |
| sp\|Q01105\|SET_HUMAN | EQQEAIEHIDEVQNEIDR.light | FALSE | 1098.01 | Unit | 175.12 | Unit | 10 | 130 | 35 | 4 | Positive |
| sp\|Q01105\|SET_HUMAN | VEVTEFEDIK.light | FALSE | 604.81 | Unit | 980.49 | Unit | 10 | 130 | 19.7 | 4 | Positive |
| sp\|Q01105\|SET_HUMAN | VEVTEFEDIK.light | FALSE | 604.81 | Unit | 881.43 | Unit | 10 | 130 | 19.7 | 4 | Positive |
| sp\|Q01105\|SET_HUMAN | VEVTEFEDIK.light | FALSE | 604.81 | Unit | 260.20 | Unit | 10 | 130 | 19.7 | 4 | Positive |
| sp\|Q01105\|SET_HUMAN | VEVTEFEDIK.light | FALSE | 604.81 | Unit | 147.11 | Unit | 10 | 130 | 19.7 | 4 | Positive |
| sp\|Q9Y4K1\|AIM1_HUMAN | TLPIQAQSQGSR.light | FALSE | 643.35 | Unit | 1071.55 | Unit | 10 | 130 | 20.9 | 4 | Positive |
| sp\|Q9Y4K1\|AIM1_HUMAN | TLPIQAQSQGSR.light | FALSE | 643.35 | Unit | 861.42 | Unit | 10 | 130 | 20.9 | 4 | Positive |
| sp\|Q9Y4K1\|AIM1_HUMAN | TLPIQAQSQGSR.light | FALSE | 643.35 | Unit | 733.36 | Unit | 10 | 130 | 20.9 | 4 | Positive |
| sp\|Q9Y4K1\|AIM1_HUMAN | TLPIQAQSQGSR.light | FALSE | 643.35 | Unit | 534.26 | Unit | 10 | 130 | 20.9 | 4 | Positive |
| sp\|Q9Y4K1\|AIM1_HUMAN | VFTFGLGK.light | FALSE | 434.75 | Unit | 769.42 | Unit | 10 | 130 | 14.5 | 4 | Positive |
| sp\|Q9Y4K1\|AIM1_HUMAN | VFTFGLGK.light | FALSE | 434.75 | Unit | 622.36 | Unit | 10 | 130 | 14.5 | 4 | Positive |
| sp\|Q9Y4K1\|AIM1_HUMAN | VFTFGLGK.light | FALSE | 434.75 | Unit | 374.24 | Unit | 10 | 130 | 14.5 | 4 | Positive |
| sp\|Q9Y4K1\|AIM1_HUMAN | VFTFGLGK.light | FALSE | 434.75 | Unit | 204.13 | Unit | 10 | 130 | 14.5 | 4 | Positive |
| sp\|Q02818\|NUCB1_HUMAN | LQAANAEDIK.light | FALSE | 536.79 | Unit | 831.42 | Unit | 10 | 130 | 17.6 | 4 | Positive |
| sp\|Q02818\|NUCB1_HUMAN | LQAANAEDIK.light | FALSE | 536.79 | Unit | 760.38 | Unit | 10 | 130 | 17.6 | 4 | Positive |
| sp\|Q02818\|NUCB1_HUMAN | LQAANAEDIK.light | FALSE | 536.79 | Unit | 260.20 | Unit | 10 | 130 | 17.6 | 4 | Positive |
| sp\|Q02818\|NUCB1_HUMAN | LQAANAEDIK.light | FALSE | 536.79 | Unit | 147.11 | Unit | 10 | 130 | 17.6 | 4 | Positive |
| sp\|Q02818\|NUCB1_HUMAN | DLAQYDAAHHEEFK.light | FALSE | 837.38 | Unit | 1083.49 | Unit | 10 | 130 | 27 | 4 | Positive |
| sp\|Q02818\|NUCB1_HUMAN | DLAQYDAAHHEEFK.light | FALSE | 837.38 | Unit | 689.33 | Unit | 10 | 130 | 27 | 4 | Positive |
| sp\|Q02818\|NUCB1_HUMAN | DLAQYDAAHHEEFK.light | FALSE | 837.38 | Unit | 294.18 | Unit | 10 | 130 | 27 | 4 | Positive |
| sp\|Q02818\|NUCB1_HUMAN | DLAQYDAAHHEEFK.light | FALSE | 837.38 | Unit | 147.11 | Unit | 10 | 130 | 27 | 4 | Positive |
| sp\|P04843\|RPN1_HUMAN | ATSFLLALEPELEAR.light | FALSE | 830.45 | Unit | 1140.63 | Unit | 10 | 130 | 26.7 | 4 | Positive |
| sp\|P04843\|RPN1_HUMAN | ATSFLLALEPELEAR.light | FALSE | 830.45 | Unit | 1027.54 | Unit | 10 | 130 | 26.7 | 4 | Positive |
| sp\|P04843\|RPN1_HUMAN | ATSFLLALEPELEAR.light | FALSE | 830.45 | Unit | 956.50 | Unit | 10 | 130 | 26.7 | 4 | Positive |
| sp\|P04843\|RPN1_HUMAN | ATSFLLALEPELEAR.light | FALSE | 830.45 | Unit | 714.38 | Unit | 10 | 130 | 26.7 | 4 | Positive |
| sp\|P04843\|RPN1_HUMAN | LAHLGVQVK.light | FALSE | 482.80 | Unit | 780.47 | Unit | 10 | 130 | 16 | 4 | Positive |
| sp\|P04843\|RPN1_HUMAN | LAHLGVQVK.light | FALSE | 482.80 | Unit | 643.41 | Unit | 10 | 130 | 16 | 4 | Positive |
| sp\|P04843\|RPN1_HUMAN | LAHLGVQVK.light | FALSE | 482.80 | Unit | 530.33 | Unit | 10 | 130 | 16 | 4 | Positive |
| sp\|P04843\|RPN1_HUMAN | LAHLGVQVK.light | FALSE | 482.80 | Unit | 147.11 | Unit | 10 | 130 | 16 | 4 | Positive |
| sp\|P49755\|TMEDA_HUMAN | ITDSAGHILYSK.light | FALSE | 652.85 | Unit | 1191.60 | Unit | 10 | 130 | 21.2 | 4 | Positive |
| sp\|P49755\|TMEDA_HUMAN | ITDSAGHILYSK.light | FALSE | 652.85 | Unit | 1090.55 | Unit | 10 | 130 | 21.2 | 4 | Positive |
| sp\|P49755\|TMEDA_HUMAN | ITDSAGHILYSK.light | FALSE | 652.85 | Unit | 234.14 | Unit | 10 | 130 | 21.2 | 4 | Positive |
| sp\|P49755\|TMEDA_HUMAN | ITDSAGHILYSK.light | FALSE | 652.85 | Unit | 147.11 | Unit | 10 | 130 | 21.2 | 4 | Positive |
| sp\|P49755\|TMEDA_HUMAN | IPDQLVILDMK.light | FALSE | 642.87 | Unit | 1171.64 | Unit | 10 | 130 | 20.9 | 4 | Positive |
| sp\|P49755\|TMEDA_HUMAN | IPDQLVILDMK.light | FALSE | 642.87 | Unit | 1074.59 | Unit | 10 | 130 | 20.9 | 4 | Positive |
| sp\|P49755\|TMEDA_HUMAN | IPDQLVILDMK.light | FALSE | 642.87 | Unit | 278.15 | Unit | 10 | 130 | 20.9 | 4 | Positive |
| sp\|P49755\|TMEDA_HUMAN | IPDQLVILDMK.light | FALSE | 642.87 | Unit | 147.11 | Unit | 10 | 130 | 20.9 | 4 | Positive |
| sp\|Q16891\|MIC60_HUMAN | YSTSGSSGLTTGK.light | FALSE | 623.30 | Unit | 1082.53 | Unit | 10 | 130 | 20.3 | 4 | Positive |
| sp\|Q16891\|MIC60_HUMAN | YSTSGSSGLTTGK.light | FALSE | 623.30 | Unit | 995.50 | Unit | 10 | 130 | 20.3 | 4 | Positive |
| sp\|Q16891\|MIC60_HUMAN | YSTSGSSGLTTGK.light | FALSE | 623.30 | Unit | 894.45 | Unit | 10 | 130 | 20.3 | 4 | Positive |
| sp\|Q16891\|MIC60_HUMAN | YSTSGSSGLTTGK.light | FALSE | 623.30 | Unit | 807.42 | Unit | 10 | 130 | 20.3 | 4 | Positive |
| sp\|Q16891\|MIC60_HUMAN | ELDSITPEVLPGWK.light | FALSE | 792.42 | Unit | 1026.56 | Unit | 10 | 130 | 25.6 | 4 | Positive |
| sp\|Q16891\|MIC60_HUMAN | ELDSITPEVLPGWK.light | FALSE | 792.42 | Unit | 925.51 | Unit | 10 | 130 | 25.6 | 4 | Positive |
| sp\|Q16891\|MIC60_HUMAN | ELDSITPEVLPGWK.light | FALSE | 792.42 | Unit | 487.27 | Unit | 10 | 130 | 25.6 | 4 | Positive |
| sp\|Q16891\|MIC60_HUMAN | ELDSITPEVLPGWK.light | FALSE | 792.42 | Unit | 147.11 | Unit | 10 | 130 | 25.6 | 4 | Positive |
| sp\|Q13011\|ECH1_HUMAN | AVVISGAGK.light | FALSE | 401.25 | Unit | 631.38 | Unit | 10 | 130 | 13.4 | 4 | Positive |
| sp\|Q13011\|ECH1_HUMAN | AVVISGAGK.light | FALSE | 401.25 | Unit | 532.31 | Unit | 10 | 130 | 13.4 | 4 | Positive |
| sp\|Q13011\|ECH1_HUMAN | AVVISGAGK.light | FALSE | 401.25 | Unit | 419.22 | Unit | 10 | 130 | 13.4 | 4 | Positive |
| sp\|Q13011\|ECH1_HUMAN | AVVISGAGK.light | FALSE | 401.25 | Unit | 204.13 | Unit | 10 | 130 | 13.4 | 4 | Positive |
| sp\|Q13011\|ECH1_HUMAN | YQETFNVIER.light | FALSE | 649.82 | Unit | 1007.52 | Unit | 10 | 130 | 21.1 | 4 | Positive |
| sp\|Q13011\|ECH1_HUMAN | YQETFNVIER.light | FALSE | 649.82 | Unit | 878.47 | Unit | 10 | 130 | 21.1 | 4 | Positive |
| sp\|Q13011\|ECH1_HUMAN | YQETFNVIER.light | FALSE | 649.82 | Unit | 777.43 | Unit | 10 | 130 | 21.1 | 4 | Positive |
| sp\|Q13011\|ECH1_HUMAN | YQETFNVIER.light | FALSE | 649.82 | Unit | 175.12 | Unit | 10 | 130 | 21.1 | 4 | Positive |
| sp\|Q5JPE7\|NOMO2_HUMAN | HHVLGTITTDK.light | FALSE | 611.33 | Unit | 1084.60 | Unit | 10 | 130 | 20 | 4 | Positive |
| sp\|Q5JPE7\|NOMO2_HUMAN | HHVLGTITTDK.light | FALSE | 611.33 | Unit | 947.54 | Unit | 10 | 130 | 20 | 4 | Positive |
| sp\|Q5JPE7\|NOMO2_HUMAN | HHVLGTITTDK.light | FALSE | 611.33 | Unit | 848.47 | Unit | 10 | 130 | 20 | 4 | Positive |
| sp\|Q5JPE7\|NOMO2_HUMAN | HHVLGTITTDK.light | FALSE | 611.33 | Unit | 147.11 | Unit | 10 | 130 | 20 | 4 | Positive |
| sp\|Q5JPE7\|NOMO2_HUMAN | AEGNDHIER.light | FALSE | 520.74 | Unit | 969.44 | Unit | 10 | 130 | 17.1 | 4 | Positive |
| sp\|Q5JPE7\|NOMO2_HUMAN | AEGNDHIER.light | FALSE | 520.74 | Unit | 840.40 | Unit | 10 | 130 | 17.1 | 4 | Positive |
| sp\|Q5JPE7\|NOMO2_HUMAN | AEGNDHIER.light | FALSE | 520.74 | Unit | 554.30 | Unit | 10 | 130 | 17.1 | 4 | Positive |
| sp\|Q5JPE7\|NOMO2_HUMAN | AEGNDHIER.light | FALSE | 520.74 | Unit | 175.12 | Unit | 10 | 130 | 17.1 | 4 | Positive |
| sp\|Q99832\|TCPH_HUMAN | ATISNDGATILK.light | FALSE | 602.33 | Unit | 918.49 | Unit | 10 | 130 | 19.7 | 4 | Positive |
| sp\|Q99832\|TCPH_HUMAN | ATISNDGATILK.light | FALSE | 602.33 | Unit | 831.46 | Unit | 10 | 130 | 19.7 | 4 | Positive |
| sp\|Q99832\|TCPH_HUMAN | ATISNDGATILK.light | FALSE | 602.33 | Unit | 602.39 | Unit | 10 | 130 | 19.7 | 4 | Positive |
| sp\|Q99832\|TCPH_HUMAN | ATISNDGATILK.light | FALSE | 602.33 | Unit | 147.11 | Unit | 10 | 130 | 19.7 | 4 | Positive |
| sp\|Q99832\|TCPH_HUMAN | TATQLAVNK.light | FALSE | 473.27 | Unit | 773.45 | Unit | 10 | 130 | 15.7 | 4 | Positive |
| sp\|Q99832\|TCPH_HUMAN | TATQLAVNK.light | FALSE | 473.27 | Unit | 544.35 | Unit | 10 | 130 | 15.7 | 4 | Positive |
| sp\|Q99832\|TCPH_HUMAN | TATQLAVNK.light | FALSE | 473.27 | Unit | 431.26 | Unit | 10 | 130 | 15.7 | 4 | Positive |
| sp\|Q99832\|TCPH_HUMAN | TATQLAVNK.light | FALSE | 473.27 | Unit | 261.16 | Unit | 10 | 130 | 15.7 | 4 | Positive |
| sp\|P27797\|CALR_HUMAN | EQFLDGDGWTSR.light | FALSE | 705.82 | Unit | 1006.46 | Unit | 10 | 130 | 22.9 | 4 | Positive |
| sp\|P27797\|CALR_HUMAN | EQFLDGDGWTSR.light | FALSE | 705.82 | Unit | 893.37 | Unit | 10 | 130 | 22.9 | 4 | Positive |
| sp\|P27797\|CALR_HUMAN | EQFLDGDGWTSR.light | FALSE | 705.82 | Unit | 778.35 | Unit | 10 | 130 | 22.9 | 4 | Positive |
| sp\|P27797\|CALR_HUMAN | EQFLDGDGWTSR.light | FALSE | 705.82 | Unit | 606.30 | Unit | 10 | 130 | 22.9 | 4 | Positive |
| sp\|P27797\|CALR_HUMAN | GLQTSQDAR.light | FALSE | 488.25 | Unit | 677.32 | Unit | 10 | 130 | 16.1 | 4 | Positive |
| sp\|P27797\|CALR_HUMAN | GLQTSQDAR.light | FALSE | 488.25 | Unit | 489.24 | Unit | 10 | 130 | 16.1 | 4 | Positive |
| sp\|P27797\|CALR_HUMAN | GLQTSQDAR.light | FALSE | 488.25 | Unit | 246.16 | Unit | 10 | 130 | 16.1 | 4 | Positive |
| sp\|P27797\|CALR_HUMAN | GLQTSQDAR.light | FALSE | 488.25 | Unit | 175.12 | Unit | 10 | 130 | 16.1 | 4 | Positive |
| sp\|P33316\|DUT_HUMAN | TDIQIALPSGC[+57.0]YGR.light | FALSE | 775.89 | Unit | 1093.55 | Unit | 10 | 130 | 25.1 | 4 | Positive |
| sp\|P33316\|DUT_HUMAN | TDIQIALPSGC[+57.0]YGR.light | FALSE | 775.89 | Unit | 980.46 | Unit | 10 | 130 | 25.1 | 4 | Positive |
| sp\|P33316\|DUT_HUMAN | TDIQIALPSGC[+57.0]YGR.light | FALSE | 775.89 | Unit | 909.42 | Unit | 10 | 130 | 25.1 | 4 | Positive |
| sp\|P33316\|DUT_HUMAN | TDIQIALPSGC[+57.0]YGR.light | FALSE | 775.89 | Unit | 796.34 | Unit | 10 | 130 | 25.1 | 4 | Positive |
| sp\|P23786\|CPT2_HUMAN | ELHEQLVALDK.light | FALSE | 647.85 | Unit | 1052.57 | Unit | 10 | 130 | 21.1 | 4 | Positive |
| sp\|P23786\|CPT2_HUMAN | ELHEQLVALDK.light | FALSE | 647.85 | Unit | 915.51 | Unit | 10 | 130 | 21.1 | 4 | Positive |
| sp\|P23786\|CPT2_HUMAN | ELHEQLVALDK.light | FALSE | 647.85 | Unit | 262.14 | Unit | 10 | 130 | 21.1 | 4 | Positive |
| sp\|P23786\|CPT2_HUMAN | ELHEQLVALDK.light | FALSE | 647.85 | Unit | 147.11 | Unit | 10 | 130 | 21.1 | 4 | Positive |
| sp\|P23786\|CPT2_HUMAN | SEYNDQLTR.light | FALSE | 563.26 | Unit | 909.44 | Unit | 10 | 130 | 18.5 | 4 | Positive |
| sp\|P23786\|CPT2_HUMAN | SEYNDQLTR.light | FALSE | 563.26 | Unit | 746.38 | Unit | 10 | 130 | 18.5 | 4 | Positive |
| sp\|P23786\|CPT2_HUMAN | SEYNDQLTR.light | FALSE | 563.26 | Unit | 632.34 | Unit | 10 | 130 | 18.5 | 4 | Positive |
| sp\|P23786\|CPT2_HUMAN | SEYNDQLTR.light | FALSE | 563.26 | Unit | 175.12 | Unit | 10 | 130 | 18.5 | 4 | Positive |
| sp\|P12268\|IMDH2_HUMAN | YEQGFITDPVVLSPK.light | FALSE | 846.95 | Unit | 1068.63 | Unit | 10 | 130 | 27.3 | 4 | Positive |
| sp\|P12268\|IMDH2_HUMAN | YEQGFITDPVVLSPK.light | FALSE | 846.95 | Unit | 955.55 | Unit | 10 | 130 | 27.3 | 4 | Positive |
| sp\|P12268\|IMDH2_HUMAN | YEQGFITDPVVLSPK.light | FALSE | 846.95 | Unit | 739.47 | Unit | 10 | 130 | 27.3 | 4 | Positive |
| sp\|P12268\|IMDH2_HUMAN | YEQGFITDPVVLSPK.light | FALSE | 846.95 | Unit | 331.20 | Unit | 10 | 130 | 27.3 | 4 | Positive |
| sp\|P12268\|IMDH2_HUMAN | EANEILQR.light | FALSE | 486.76 | Unit | 772.43 | Unit | 10 | 130 | 16.1 | 4 | Positive |
| sp\|P12268\|IMDH2_HUMAN | EANEILQR.light | FALSE | 486.76 | Unit | 529.35 | Unit | 10 | 130 | 16.1 | 4 | Positive |
| sp\|P12268\|IMDH2_HUMAN | EANEILQR.light | FALSE | 486.76 | Unit | 416.26 | Unit | 10 | 130 | 16.1 | 4 | Positive |
| sp\|P12268\|IMDH2_HUMAN | EANEILQR.light | FALSE | 486.76 | Unit | 175.12 | Unit | 10 | 130 | 16.1 | 4 | Positive |
| sp\|P02751\|FINC_HUMAN | HYQINQQWER.light | FALSE | 701.34 | Unit | 1101.54 | Unit | 10 | 130 | 22.7 | 4 | Positive |
| sp\|P02751\|FINC_HUMAN | HYQINQQWER.light | FALSE | 701.34 | Unit | 973.49 | Unit | 10 | 130 | 22.7 | 4 | Positive |
| sp\|P02751\|FINC_HUMAN | HYQINQQWER.light | FALSE | 701.34 | Unit | 860.40 | Unit | 10 | 130 | 22.7 | 4 | Positive |
| sp\|P02751\|FINC_HUMAN | HYQINQQWER.light | FALSE | 701.34 | Unit | 175.12 | Unit | 10 | 130 | 22.7 | 4 | Positive |
| sp\|P02751\|FINC_HUMAN | TYHVGEQWQK.light | FALSE | 638.31 | Unit | 874.44 | Unit | 10 | 130 | 20.8 | 4 | Positive |
| sp\|P02751\|FINC_HUMAN | TYHVGEQWQK.light | FALSE | 638.31 | Unit | 775.37 | Unit | 10 | 130 | 20.8 | 4 | Positive |
| sp\|P02751\|FINC_HUMAN | TYHVGEQWQK.light | FALSE | 638.31 | Unit | 275.17 | Unit | 10 | 130 | 20.8 | 4 | Positive |
| sp\|P02751\|FINC_HUMAN | TYHVGEQWQK.light | FALSE | 638.31 | Unit | 147.11 | Unit | 10 | 130 | 20.8 | 4 | Positive |
| sp\|Q14938\|NFIX_HUMAN | AFSYTWFNLQAR.light | FALSE | 752.37 | Unit | 1035.54 | Unit | 10 | 130 | 24.3 | 4 | Positive |
| sp\|Q14938\|NFIX_HUMAN | AFSYTWFNLQAR.light | FALSE | 752.37 | Unit | 934.49 | Unit | 10 | 130 | 24.3 | 4 | Positive |
| sp\|Q14938\|NFIX_HUMAN | AFSYTWFNLQAR.light | FALSE | 752.37 | Unit | 748.41 | Unit | 10 | 130 | 24.3 | 4 | Positive |
| sp\|Q14938\|NFIX_HUMAN | AFSYTWFNLQAR.light | FALSE | 752.37 | Unit | 175.12 | Unit | 10 | 130 | 24.3 | 4 | Positive |
| sp\|Q14938\|NFIX_HUMAN | SITSPPSTSTTK.light | FALSE | 603.81 | Unit | 1006.51 | Unit | 10 | 130 | 19.7 | 4 | Positive |
| sp\|Q14938\|NFIX_HUMAN | SITSPPSTSTTK.light | FALSE | 603.81 | Unit | 818.43 | Unit | 10 | 130 | 19.7 | 4 | Positive |
| sp\|Q14938\|NFIX_HUMAN | SITSPPSTSTTK.light | FALSE | 603.81 | Unit | 721.37 | Unit | 10 | 130 | 19.7 | 4 | Positive |
| sp\|Q14938\|NFIX_HUMAN | SITSPPSTSTTK.light | FALSE | 603.81 | Unit | 147.11 | Unit | 10 | 130 | 19.7 | 4 | Positive |
| sp\|P80303\|NUCB2_HUMAN | AATSDLEHYDK.light | FALSE | 625.29 | Unit | 1107.50 | Unit | 10 | 130 | 20.4 | 4 | Positive |
| sp\|P80303\|NUCB2_HUMAN | AATSDLEHYDK.light | FALSE | 625.29 | Unit | 1006.45 | Unit | 10 | 130 | 20.4 | 4 | Positive |
| sp\|P80303\|NUCB2_HUMAN | AATSDLEHYDK.light | FALSE | 625.29 | Unit | 562.26 | Unit | 10 | 130 | 20.4 | 4 | Positive |
| sp\|P80303\|NUCB2_HUMAN | AATSDLEHYDK.light | FALSE | 625.29 | Unit | 147.11 | Unit | 10 | 130 | 20.4 | 4 | Positive |
| sp\|P80303\|NUCB2_HUMAN | LVTLEEFLK.light | FALSE | 546.32 | Unit | 879.48 | Unit | 10 | 130 | 17.9 | 4 | Positive |
| sp\|P80303\|NUCB2_HUMAN | LVTLEEFLK.light | FALSE | 546.32 | Unit | 778.43 | Unit | 10 | 130 | 17.9 | 4 | Positive |
| sp\|P80303\|NUCB2_HUMAN | LVTLEEFLK.light | FALSE | 546.32 | Unit | 665.35 | Unit | 10 | 130 | 17.9 | 4 | Positive |
| sp\|P80303\|NUCB2_HUMAN | LVTLEEFLK.light | FALSE | 546.32 | Unit | 147.11 | Unit | 10 | 130 | 17.9 | 4 | Positive |
| sp\|P17987\|TCPA_HUMAN | SSLGPVGLDK.light | FALSE | 486.77 | Unit | 685.39 | Unit | 10 | 130 | 16.1 | 4 | Positive |
| sp\|P17987\|TCPA_HUMAN | SSLGPVGLDK.light | FALSE | 486.77 | Unit | 628.37 | Unit | 10 | 130 | 16.1 | 4 | Positive |
| sp\|P17987\|TCPA_HUMAN | SSLGPVGLDK.light | FALSE | 486.77 | Unit | 432.25 | Unit | 10 | 130 | 16.1 | 4 | Positive |
| sp\|P17987\|TCPA_HUMAN | SSLGPVGLDK.light | FALSE | 486.77 | Unit | 147.11 | Unit | 10 | 130 | 16.1 | 4 | Positive |
| sp\|P17987\|TCPA_HUMAN | LLEVEHPAAK.light | FALSE | 553.81 | Unit | 880.45 | Unit | 10 | 130 | 18.2 | 4 | Positive |
| sp\|P17987\|TCPA_HUMAN | LLEVEHPAAK.light | FALSE | 553.81 | Unit | 751.41 | Unit | 10 | 130 | 18.2 | 4 | Positive |
| sp\|P17987\|TCPA_HUMAN | LLEVEHPAAK.light | FALSE | 553.81 | Unit | 386.24 | Unit | 10 | 130 | 18.2 | 4 | Positive |
| sp\|P17987\|TCPA_HUMAN | LLEVEHPAAK.light | FALSE | 553.81 | Unit | 147.11 | Unit | 10 | 130 | 18.2 | 4 | Positive |
| sp\|Q92598\|HS105_HUMAN | ENLSYDLVPLK.light | FALSE | 645.85 | Unit | 934.52 | Unit | 10 | 130 | 21 | 4 | Positive |
| sp\|Q92598\|HS105_HUMAN | ENLSYDLVPLK.light | FALSE | 645.85 | Unit | 357.25 | Unit | 10 | 130 | 21 | 4 | Positive |
| sp\|Q92598\|HS105_HUMAN | ENLSYDLVPLK.light | FALSE | 645.85 | Unit | 260.20 | Unit | 10 | 130 | 21 | 4 | Positive |
| sp\|Q92598\|HS105_HUMAN | ENLSYDLVPLK.light | FALSE | 645.85 | Unit | 147.11 | Unit | 10 | 130 | 21 | 4 | Positive |
| sp\|O15355\|PPM1G_HUMAN | ALEDAFLAIDAK.light | FALSE | 638.84 | Unit | 1092.56 | Unit | 10 | 130 | 20.8 | 4 | Positive |
| sp\|O15355\|PPM1G_HUMAN | ALEDAFLAIDAK.light | FALSE | 638.84 | Unit | 777.45 | Unit | 10 | 130 | 20.8 | 4 | Positive |
| sp\|O15355\|PPM1G_HUMAN | ALEDAFLAIDAK.light | FALSE | 638.84 | Unit | 630.38 | Unit | 10 | 130 | 20.8 | 4 | Positive |
| sp\|O15355\|PPM1G_HUMAN | ALEDAFLAIDAK.light | FALSE | 638.84 | Unit | 517.30 | Unit | 10 | 130 | 20.8 | 4 | Positive |
| sp\|O15355\|PPM1G_HUMAN | QLIVANAGDSR.light | FALSE | 572.31 | Unit | 902.47 | Unit | 10 | 130 | 18.7 | 4 | Positive |
| sp\|O15355\|PPM1G_HUMAN | QLIVANAGDSR.light | FALSE | 572.31 | Unit | 789.38 | Unit | 10 | 130 | 18.7 | 4 | Positive |
| sp\|O15355\|PPM1G_HUMAN | QLIVANAGDSR.light | FALSE | 572.31 | Unit | 690.32 | Unit | 10 | 130 | 18.7 | 4 | Positive |
| sp\|O15355\|PPM1G_HUMAN | QLIVANAGDSR.light | FALSE | 572.31 | Unit | 262.15 | Unit | 10 | 130 | 18.7 | 4 | Positive |
